# Application of a novel metaheuristic algorithm inspired by stadium spectators in global optimization problems

**DOI:** 10.1038/s41598-024-53602-2

**Published:** 2024-02-06

**Authors:** Mehrdad Nemati, Yousef Zandi, Alireza Sadighi Agdas

**Affiliations:** Department of Civil Engineering, Tabriz Branch, Islamic Azad University, Tabriz, Iran

**Keywords:** Engineering, Civil engineering

## Abstract

This paper presents a novel metaheuristic algorithm inspired by the actions of stadium spectators affecting behavior of players during a match which will be called stadium spectators optimizer (SSO) algorithm. The mathematical model of the SSO algorithm is presented and the performance and efficiency of the presented method is tested on some of the well-known mathematical test functions and also CEC-BC-2017 functions. The SSO algorithm is a parameter-free optimization method since it doesn't require any additional parameter setup at any point throughout the optimization process. It seems urgently necessary to design a novel metaheuristic algorithm that is parameter-free and capable of solving any optimization problem without taking into account extra parameters, as the majority of metaheuristic algorithms rely on the configuration of extra parameters to solve different problems efficiently. A positive point for the SSO algorithm can be seen in the results of the suggested technique, which indicate a partial improvement in performance. The results are compared with those of golf optimization algorithm (GOA), Tiki taka optimization algorithm (TTA), Harris Hawks optimization algorithm (HHO), the arithmetic optimization algorithm (AOA), CMA-ES and EBOwithCMAR algorithms. The statistical tests are carried out for the obtained results and the tests reveal the capability of the presented method in solving different optimization problems with different dimensions. SSO algorithm performs comparably and robustly with the state-of-the-art optimization techniques in 14 of the mathematical test functions. For CEC-BC-2017 functions with ten dimensions, EBOwithCMAR performs better than the proposed method. However, for most functions of CEC-BC-2017 with ten dimensions, the SSO algorithm ranks second after EBOwithCMAR, which is an advantage of the SSO since the proposed method performs better than the well-known CMA-ES optimization algorithm. The overall performance of the SSO algorithm in CEC-BC-2017 functions with 10 dimensions was acceptable, in dimension of 30, 50 and 100, the performance of the proposed method in some functions decreased.

## Introduction

There is an increasing need for solving optimization problems and optimum design in engineering and science^1–3^. It can be clearly perceived that in order to solve new problems in optimization field, we need to find novel methods if the existing algorithms perform weak on those problems^4^. There are traditional methods such as linear and non-linear programming^5^, as well as new methods inspired by nature which are called metaheuristics^6,7^. Traditional optimization algorithms need to have information on a promising initial starting point and gradient in order to successfully optimize a function. On the other side, metaheuristic algorithms can be successful in solving a set of optimization problems and unable to provide satisfactory solutions for some other optimization problems, which makes metaheuristic algorithms highly problem dependent.

Optimum design and global optimization problems are quite difficult to deal with. In order to increase the efficiency of systems and human resources, various problems of everyday life need to be carefully considered and tackled. These problems can be classified into constrained and unconstrained problems. Novel methods have been introduced by researchers for solving optimization problems with a great performance in different fields. These methods offer a high accuracy and speed for solving different optimization problems in an efficient way.

These days, metaheuristic algorithms are used to optimize complex real-world scientific and engineering problems; they all aim to favor the search for the global optimum solution rather than local ones and can deal with constraints much more easily than traditional methods because they do not require complex mathematical expressions. In recent years, academics have employed metaheuristic algorithms to optimize and solve a wide range of complex engineering problems.

The process of optimizing a system's parameters to maximize or decrease its output involves selecting the best values from all potential values for that system. Because the optimization problem has prompted the creation of optimization techniques and the growth of fascinating research topics, it may be seen in many fields of study. Metaheuristic algorithms view a problem as a black box, therefore they are widely used. Metaheuristic algorithms are referred to be "nature inspired" because its primary foundation is found in the rules of living things. Metaheuristic algorithms fall into four categories based on the type of simulated phenomenon they are used for: physics-based, swarm intelligence, evolutionary, and behavior-inspired.

Artificial intelligence experts have proposed to solve complex optimization problems with metaheuristics, which was mentioned before and refers to higher-level strategies implemented to do search strategies in optimization problems. Metaheuristic optimization algorithms use different search strategies in solving optimization problems and an optimizer algorithm is properly designed for exploring search space of an optimization problem to find an optimum solution. Several innovative metaheuristic algorithms have been proposed in recent years, including: SCMWOA^8^, Immune Plasma Algorithm^9^, The Solar System Algorithm^10^, Ebola Optimization Search Algorithm^11^, War Strategy Optimization Algorithm^12^, Crystal Structure Algorithm^13^, Ali Baba and the forty thieves^14^, MoSSE^15^, ARSH-FATI^16^, Wingsuit Flying search^17^, The Archerfish Hunting Optimizer^18^, Chaos Game optimization^19^, Archimedes optimization algorithm^20^, Fire Hawk Optimizer^21^, GOZDE^22^, bold eagle search optimization algorithm^23^, bear smell search algorithm^24^, Black Widow optimization algorithm^25^, Dung beetle optimizer algorithm^26^, Artificial ecosystem-based optimization^27^, Artificial lizard search optimization^28^, Golden ratio optimization method^29^, tree optimization algorithm^30^, Sea-Horse optimizer^31^, Gaining-sharing knowledge based algorithm^32^, Honey Badger Algorithm^33^, Atomic Orbital search^34^, Spider wasp optimizer^35^, Group teaching optimization algorithm^36^, Human urbanization algorithm^37^, Woodpecker Mating Algorithm^38,39^, Hybrid SCA-WMA Algorithm^40^, HWMWOA^41^, GWMA^42^, hierarchical multi-leadership sine cosine algorithm^43^.

Population-based metaheuristic algorithms have two characteristics: exploration and exploitation. An algorithm should have specific mechanisms in place during the exploration phase to enable it to search the whole search space. Actually, it is during this phase that the search space's potential regions are found. The goal of exploration is to find specific regions of the search space that have the best overall answer and to examine the problem from a global perspective. Following exploration is the exploitation stage. It is the process of examining a potentially promising location that was discovered during the exploration stage. These Two opposed turning points are exploration and exploitation. Encouraging the outcomes of one stage devalues the outcomes of the other. By employing population-based algorithms, an accurate prediction of the global optimum may be ensured by maintaining the right balance between these two turning points.

According to the “No Free Lunch” theorem, there is no single algorithm capable of providing efficient solutions for all optimization problems^44^. Therefore, investigating and modifying existing algorithms for solving optimization problems is a highly-active research field since more information is needed on the efficiency of metaheuristic algorithms and their capability of providing good solutions to various real-life optimization problems^45–47^. Improving and inventing metaheuristic algorithms over the past few decades was extremely important for researchers of the field and since the real-world optimization problems are becoming more complex and their dimensions are expanding, developing novel methods capable of solving extremely complex optimization problems is in the center of the attention of optimization algorithm development and research^48^.

Since metaheuristic algorithms are gradient-free, stochastic, able to escape from local minima and can be easily implemented, they can be a good alternative for traditional algorithms such as gradient based and quadratic programming^49^. Furthermore, metaheuristic algorithms use a population of search agents in order to search the domain using probabilistic rather than some deterministic rules of deterministic algorithms^50^. Exploration and exploitation ability of metaheuristic algorithms are indeed very important since at the end of the algorithm, obtaining an efficient and accurate search for optimum solutions depends on the tradeoff between these two^20^.

In this study we present a novel metaheuristic algorithm which will be called stadium spectators optimizer algorithm (SSO) which is inspired by human behaviors in watching sports in a stadium where the spectators communicate with each other inside of stadium and also sharing their opinions with others outside of stadium. The SSO algorithm relies on no extra parameter setup throughout the optimization process, making it a parameter-free optimization technique. This paper's primary contribution is the innovative search approach it proposes for optimization, which involves an intelligent procedure to identify the optimal solutions of various global optimization problems. The modeling of what occurs in a stadium during a game, where fans are the primary influencing element for cheering and supporting their preferred team, as well as the outcomes of that cheering during the entire event, served as the inspiration for the suggested algorithm. If fans' preferred team scores more points as a result of their cheering, this may result in stronger solution candidates and, ultimately, an improved value for the objective function. If spectators dominate the match, the current solution is getting closer to the best one that has been developed so far. The best solution moves toward the player or players who are receiving special attention from the audience; if not, it moves toward random players. According to the suggested strategy, every iteration always selects two players at random, chosen by spectators, to decide how the other players move. This determines the events that will happen during the competition, such as their team scoring or their opponent scoring. As a result, the viewers' actions will have an impact on the players, leading to varying actions and scores, which ultimately decide the outcome of the match.

The presented algorithm can be categorized in the human-behavior based metaheuristic algorithms and it is a population-based method, free of other parameters which makes it easy to implement. In order to evaluate the robustness of the presented method, twenty-three test functions and test suite of CEC-BC-2017 (with every size conceivable 10, 30, 50, and 100) are investigated by the proposed method. The performance of the presented algorithm is compared with some of the well-known state of the art optimization algorithms and the results are presented and discussed. Furthermore, some statistical tests are conducted to assess the effectiveness of the suggested approach.

## Literature review

The Sine Cosine hybrid optimization technique with the Modified Whale Optimization technique (SCMWOA) was proposed to address issues involving continuous and binary decision variables by combining the advantages of WOA and SCA. The recent global health crisis also known as the COVID-19 or coronavirus pandemic has attracted the researchers’ attentions to a treatment approach called immune plasma or convalescent plasma once more again. The main idea lying behind the immune plasma treatment is transferring the antibody rich part of the blood taken from the patients who are recovered previously, to the critical individuals and its efficiency has been proven by successfully using against great influenza of 1918, H1N1 flu, MERS, SARS and Ebola. This prompted scientists studying optimization, to create the Immune Plasma (IP) algorithm, a novel meta-heuristic. The Solar System Algorithm (SSA), a novel metaheuristic algorithm for global optimization, was introduced. It mimics the circling behavior of several solar system objects, such as the Sun, planets, moons, stars, and black holes. The Ebola Optimization Search Algorithm (EOSA), a new metaheuristic algorithm based on the Ebola virus disease's mode of propagation, was introduced. military Strategy Optimization (WSO), a new metaheuristic optimization algorithm based on antiquated military strategy, was put forth. It is based on the army's strategic troop movement during the conflict. An optimization method is used to model military strategy, in which each soldier advances dynamically in the direction of the ideal outcome. The innovative metaheuristic known as Crystal Structure method (CryStAl) was proposed with the goal of creating an extremely efficient optimization method that draws inspiration from nature. The symmetric arrangement of constituents (i.e., atoms, molecules, or ions) in crystalline minerals like quartz is a natural phenomenon that serves as the primary inspiration for this method. This formation of crystal structures is based on the same principles. Inspired by the well-known story of Ali Baba and the forty thieves, in which Ali Baba once witnessed a group of forty robbers enter a strange cave full of various treasures, a unique meta-heuristic algorithm named Ali Baba and the forty thieves (AFT) was presented for tackling global optimization issues. A unique hybrid optimization technique known as MoSSE, which combines the capabilities of Salp Swarm technique (SSA), Emperor Penguin Optimizer (EPO), and Multi-objective Spotted Hyena Optimizer (MOSHO) was introduced. MoSSE employs the following techniques: EPO's efficient mover approach for better adjustment of the next solution; SSA's leadership and selection process to get the fittest global solution with faster convergence technique; and MOSHO's searching capabilities to effectively explore the search space. A new technique known as ARSH-FATI-based Cluster Head Selection (ARSH-FATI-CHS) was created, combining a heuristic known as novel ranked-based clustering (NRC) to effectively improve the LT of the network while lowering the communication energy usage of the sensor nodes. In contrast to existing population-based algorithms, ARSH-FATI-CHS dynamically alternates between search process exploration and exploitation during run-time in order to achieve a higher trade-off between performance and a large rise in the network's LT. Wingsuit Flying Search (WFS) a revolutionary global optimization algorithm was introduced in which the well-liked extreme sport of wingsuit flying served as inspiration. The method simulates a flier's desire to land at the lowest location in their range on Earth's surface, or a global minimum of the search space. This is accomplished by using a properly chosen population of points to probe the search space at each iteration. The flier gradually gains a crisper image of the surface as a result of the iterative population update, which causes the attention to move to lower regions. The archerfish hunting optimizer (AHO) is a novel metaheuristic algorithm for global optimization that was developed based on the shooting and jumping actions of archerfish, which are used to hunt aerial insects. The swapping angle and the attractiveness rate are the two parameters that must be set for the AHO algorithm. To solve optimization difficulties, a brand-new metaheuristic algorithm known as Chaos Game Optimization (CGO) was created. The primary idea behind the CGO algorithm is grounded in some chaos theory ideas, which put the self-similarity problems with fractals and the configuration of fractals by the chaos game notion in context. To address the optimization issues, a brand-new metaheuristic method known as the Archimedes optimization algorithm (AOA) was presented. Archimedes' Principle, an intriguing law of physics, served as an inspiration for the development of AOA. It mimics the idea that the buoyant force applied to an object—whether submerged entirely or only partially—is proportionate to the weight of the displaced fluid.

## Stadium spectators optimizer algorithm

### Inspiration

Before the beginning of a match in a stadium, usually people are around the stadium and near to the entrance of it. They may speak in different languages, have common interests in cheering their favorite team, take pictures of themselves with their favorite players and share them with others both inside and outside of the stadium, check the results and news about other teams in the internet, change their slogan in stadium for encouraging their team to score more or showing their dissatisfaction of their favorite team because of their weak performance. All these behaviors and doings, can influence the team performance in achieving a good or bad result during a match. Therefore, if an action by the spectators which are considered as search agents, cause a behavior in members of a team which the behavior is directly connected to objective function quality of an optimization problem being solved by the algorithm, it can be considered as a candidate solution for the optimization problem being tackled. The better quality of objective function will be perceived by spectators and can be used to cheer the team for scoring more and getting advanced of the opponent team.

Showing excitement during a match from fans and spectators will affect the behavior of players in both teams. Any behavior of players will have its own particular objective function value which at the end, the scores will determine the winner of the match, and the end of the match is determined by the algorithm termination criterion which in this case is the maximum number of iterations of the algorithm. The population of the presented algorithm is the number of spectators present in stadium and their actions will have a reaction from players. Different search strategies will be carried out according to the action-reaction relationship between spectators and players.

### Mathematical model

The mathematical modeling of the SSO algorithm is presented in this section. The first stage begins with the initializations of candidate solutions (X_i_) being the reaction of players in stadium to actions of spectators. This can be mathematically expressed as the following:

1 $$X = \left[ {\begin{array}{*{20}c} {X_{1} } \\ {X_{2} } \\ \vdots \\ {X_{i} } \\ \vdots \\ {X_{n} } \\ \end{array} } \right] = \left[ {\begin{array}{*{20}c} {x_{1}^{1} } & {x_{1}^{2} } & \ldots & {x_{1}^{j} } & \ldots & {x_{1}^{d} } \\ {x_{2}^{1} } & {x_{2}^{2} } & \ldots & {x_{2}^{j} } & \ldots & {x_{2}^{d} } \\ \vdots & \vdots & \vdots & \vdots & \ddots & \vdots \\ {x_{i}^{1} } & {x_{i}^{2} } & \ldots & {x_{i}^{j} } & \ldots & {x_{i}^{d} } \\ \vdots & \vdots & \vdots & \vdots & \ddots & \vdots \\ {x_{n}^{1} } & {x_{n}^{2} } & \ldots & {x_{n}^{j} } & \ldots & {x_{n}^{d} } \\ \end{array} } \right],\quad \left\{ { \begin{array}{*{20}c} {i = 1,2, \ldots ,n.} \\ {j = 1,2, \ldots ,d.} \\ \end{array} } \right.$$

2 $$x_{i}^{j} = x_{i,min}^{j} + rand.\left( {x_{i,max}^{j} - x_{i,min}^{j} } \right), \left\{ { \begin{array}{*{20}c} { i = 1,2, \ldots ,n.} \\ { j = 1,2, \ldots ,d.} \\ \end{array} } \right.$$

Where n shows the number of candidate solutions of the SSO algorithm and d indicates the optimization problem dimension, ${x}_{i}^{j}$ shows the jth decision variable for specifying the ith candidate’ initial position,${x}_{i,max}^{j}$ and ${x}_{i,min}^{j}$ are the upper and lower bounds of the jth variable in the ith candidate in optimization problem, respectively and “*rand”* is a random number uniformly distributed within the range of zero and one.

The mathematical model expressed in the Eqs. (1) and (2), are used to create the initial search agents in the search space of optimization problem. They also will provide different reactions in players, causing different values of objective function. For each candidate solution (X_i_), at the next iterations of the algorithm, different values will be replaced in order to tweak the population of the algorithm for exploring and exploiting different regions of the search space.

At each iteration of the SSO algorithm, two random search agents are chosen among the population for conducting a search strategy by the algorithm. The search agent related to the best so far found solution is also chosen to help conducting the search strategy. A random value between zero and one will determine whether to explore or exploit the search space and therefore for exploitation purpose, the SSO algorithm will move from the best solution to a space between the two sleeted random search agents (providing two other solutions of optimization problem). For exploration, the search agent will move from the best solution towards a point in a different and far location in the search space or in other words it will jump from the best search agent’s position towards another position in the search space causing a different behavior in players’ reaction to spectators’ actions. Both of these behaviors of the algorithm and the values obtained from them are stored in a vector called RND. It can be mathematically stated as the following:

3 $$\overrightarrow {{ RND_{i} }} = \left\{ {\begin{array}{*{20}l} {\overrightarrow {{ {\text{XB}}_{i} }} + \overrightarrow {{ (X_{R1} }} - \overrightarrow {{ X_{R2} )}} if \,HR \le 0.5} \hfill \\ {\overrightarrow {{ {\text{XB}}_{i} }} + \overrightarrow { R} otherwise} \hfill \\ \end{array} } \right.$$

where $\overrightarrow{{{\text{XB}}}_{i}}$ is the best solution so far found, $\overrightarrow{{X}_{R1}}$ and $\overrightarrow{{X}_{R2}}$ are the two selected random search agents, HR is a random number in the interval of zero and one and $\overrightarrow{R}$ is a vector of uniformly distributed random numbers in the interval of zero and one. In order to control the movements of search agents in the algorithm and providing a balance between the exploration and exploitation phases of the SSO algorithm, a parameter is used to control the diversification and intensification of the algorithm. This parameter is mathematically expressed as the following:

4 $$LI = {1} - \left( {{\text{I}}/{\text{Im}}} \right)$$

where *I* is the current number of iterations and Im is the maximum, number of iterations of the SSO algorithm. Considering the aforementioned matters, the updating of the solutions in each iteration is done through the following mathematical expression:

5 $$\overrightarrow {{ NewPosition_{i} }} = \overrightarrow {{ {\text{X}}_{i} }} + \overrightarrow {r1} \cdot LI \cdot \left( {mu1 \otimes \overrightarrow {r2} \cdot (\overrightarrow {{ {\text{XB}}_{i} }} - \overrightarrow {r3} \otimes \overrightarrow {{ X_{R1} }} ) + \overrightarrow {r4} \cdot \left( {\overrightarrow {r5} \cdot {\text{mu}}2 \otimes \left( {{\text{rn}}1 \cdot \overrightarrow {{ RND_{i} }} - \overrightarrow {{ X_{R2} }} } \right)} \right)} \right)\quad {\text{i}} = {1},{2}, \ldots ,{\text{n}}$$

Where r1, r2, r3, r4 and r5 are random vector numbers in the interval of zero and one; mu1, mu2 and rn1 are random numbers with normal distributions.

New positions at each iteration are determined through moving the previous positions of the optimization problem, towards a point between the best so far found solution and one of the two random selected search agents by providing a stochastic behavior causing the agents of the algorithm moving randomly in that region. The search agents also move towards a place between the RND vector and the other randomly selected search agent, to conduct a more precise search in the search space of problem. This will assure that a good exploration will be conducted at the first levels of the search process of the SSO algorithm. “LI” will cause an exploitative behavior of the algorithm at the final stages of search process and it assures of the local search of promising regions of the search space.

For controlling the violations of decision variables from the defined lower and upper bounds in optimization problem, a mathematical flag is defined to modify the violated decision variables and bring them back to the search space. The termination criterion of the algorithm is determined by a fixed number of iterations. The step by step implementation procedure of the algorithm with the pseudo-code of the SSO algorithm are presented in the following:

- Step 1: Initialize positions of the candidate solutions in the search space randomly.
- Step 2: Evaluate the objective function’s values of the initial positions and store them.
- Step 3: Determine the best solution so far found by the SSO algorithm which contains the important position in the search space and later will be used to determine new positions at the next iterations.
- Step 4: Determine the RND vector based on a probability mechanism provided in the SSO algorithm.
- Step 5: Determine the new positions by moving the current solutions available towards the new positions explained in the Eq. (5).
- Step 6: Check the boundary conditions of problem.
- Step 7: Calculate the new values of the objective function of problem.
- Step 8: Substitute the worst solutions with better solutions found at the new iteration.
- Step 9: check the termination criterion of the algorithm.

**Figure 1:**
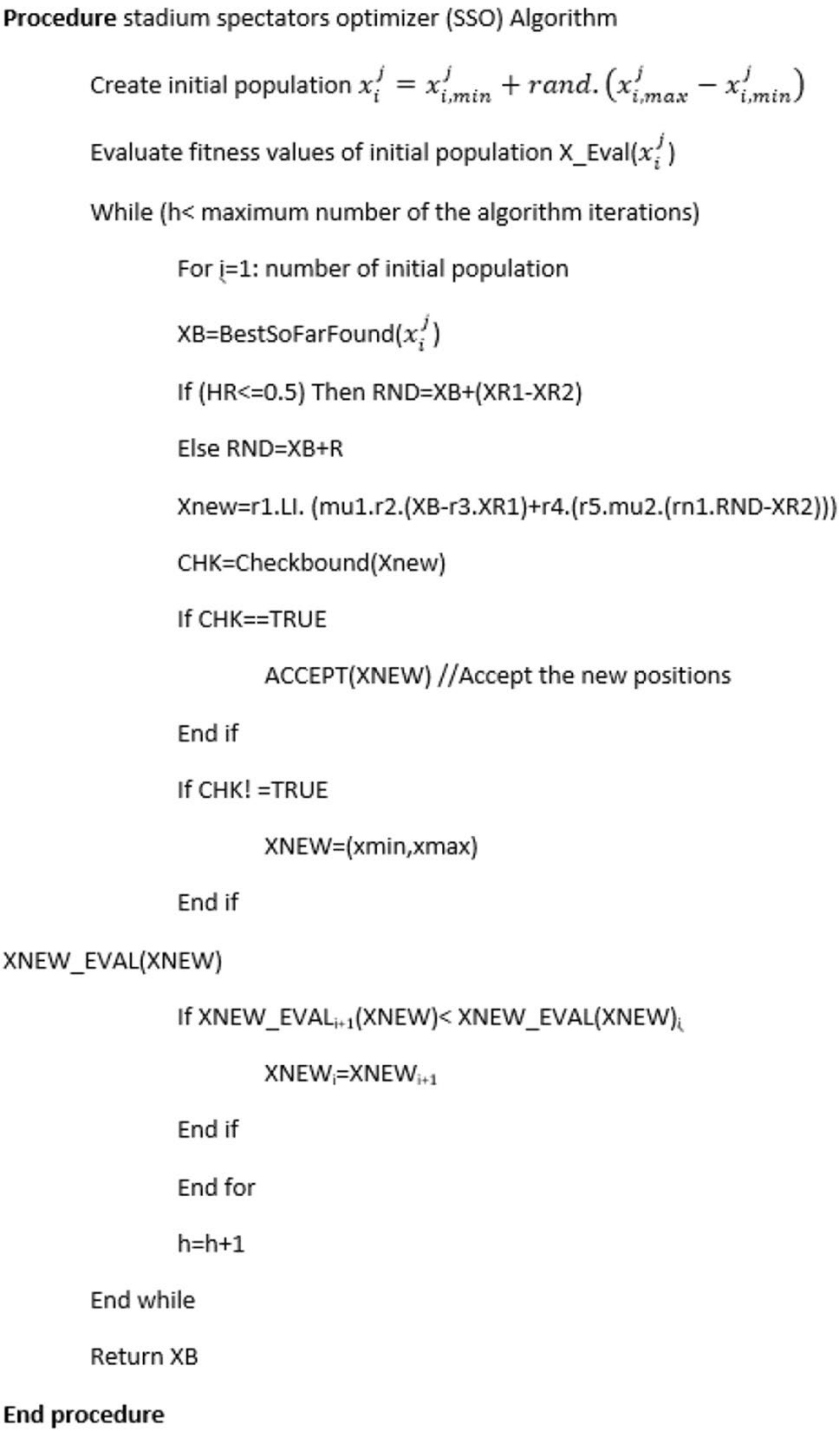
The pseudo-code of SSO algorithm.

The pseudo code of the SSO algorithm is shown in Fig. 1:

## Mathematical test functions

For conducting an assessment of the proposed method in this paper, different test functions are used to evaluate the SSO’s capability for doing exploration and exploitation alongside the ability of the algorithm in escaping from local minima. This is done by testing the SSO algorithm over the benchmarks which have fixed, multimodal and unimodal dimensions. The test functions F1-F7 can determine and evaluate the exploitation ability of the SSO algorithm, test functions F8-F13 can be used to test the exploration ability of the proposed algorithm, and in order to evaluate the exploration of the SSO algorithm in low-dimensional optimization problems, functions F14-F23 are tested by the SSO. All of these mathematical test functions can be in general a good assessment of the performance of the SSO algorithm in a real-world optimization problem since the mathematical test functions have too many local optima in their search spaces. The details of these test functions are available in study of Liang et al.^51^.

The maximum number of iterations in this study is set to 1000 and the mathematical test functions are evaluated 150,000 times and for the purpose of having an understandable statistical test results, the SSO algorithm was ran 30 times over each of the mathematical test functions. The assumption is that the suggested algorithm or the compared algorithms have reached the global optimum point when they are close to the global optimum value of the objective functions given in the mathematical test functions with a tolerance of 1E–12. The results of this experiment, provide mean and standard deviation values of the minimum values obtained in each run for all of the mathematical test functions. Some of the other well-known existing optimization algorithms are also used to compare the performance of the SSO algorithm with them and also to analyze the efficiency and superiority of the proposed method over some of the existing ones. The algorithms used to do this are as the following: Golf optimization^52^, Tiki-taka algorithm^53^, Harris hawks optimization^54^, The arithmetic optimization^55^ algorithms. Table 1 shows the parameters used for each of these algorithms in the experiments. The values of the parameters are set as per recommended values by their developers.

**Table 1:** Parameters of the algorithms used to compare the results with the SSO algorithm.

Algorithms	Parameter	Definition	Value
GOA	Npop	Population size	50
TTA	Npop	Population size	50
HHO	Npop	Population size	50
AOA	Npop	Population size	50
	α	Exploitation accuracy	5
	MOP_min_	Math optimizer probability (min)	0.2
	MOP_max_	Math optimizer probability (Max)	1
	μ	Control parameter to adjust the search process	0.5
CMA-ES	Npop	Population size	50
EBOwithCMAR	Npop	Population size	50

Figures 2, 3, 4, 5, 6, 7, 8, 9, 10, 11, 12, 13, 14, 15, 16, 17, 18, 19, 20, 21, 22, 23, 24 show the convergence curves of mathematical test functions of all algorithms.

**Figure 2:**
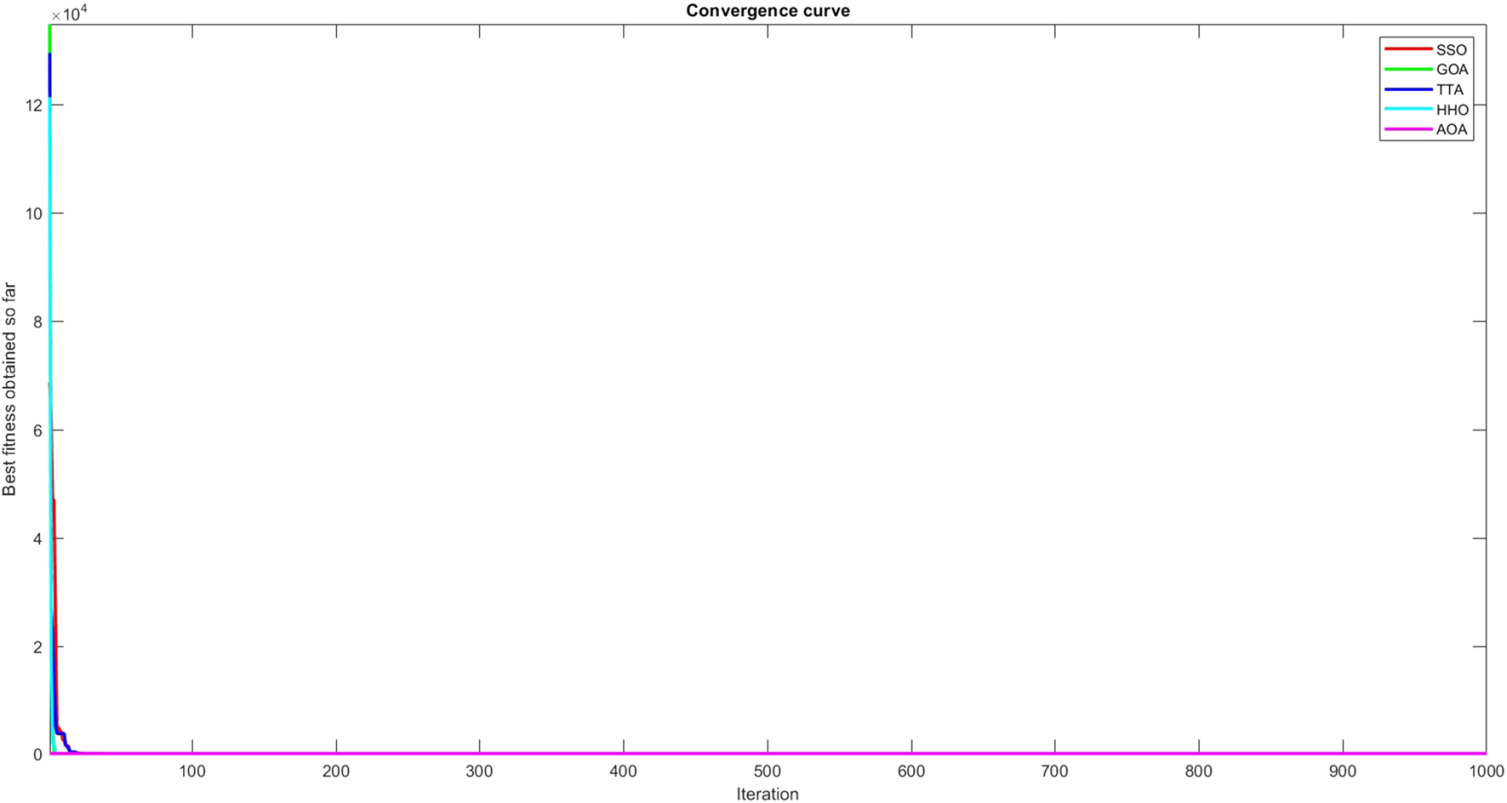
Mathematical test function 1.

**Figure 3:**
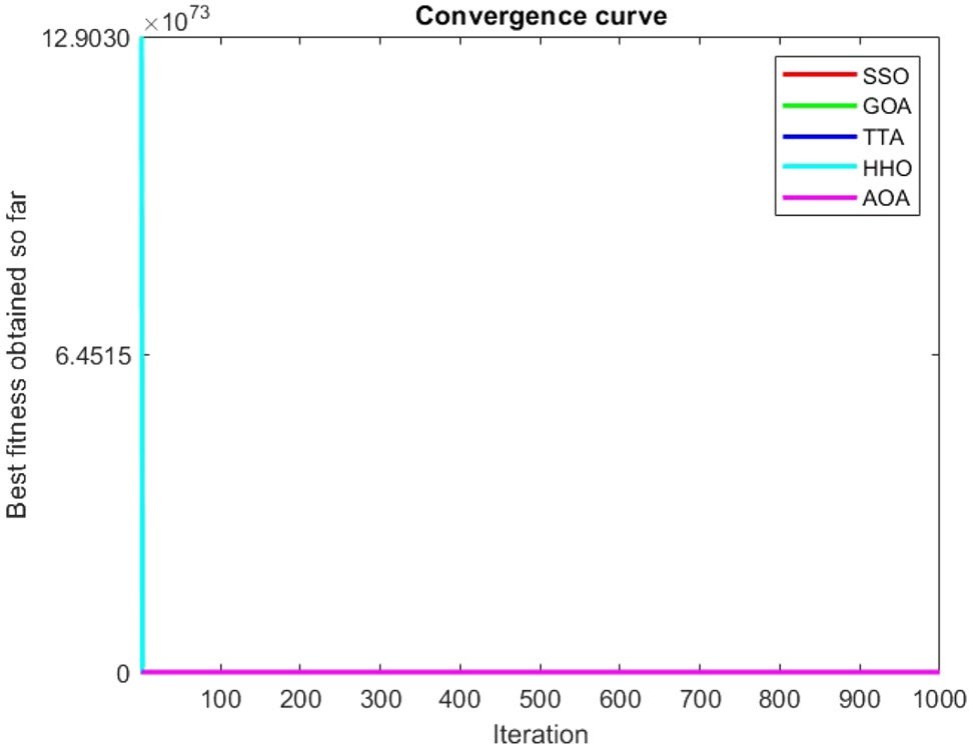
mathematical test function 2.

**Figure 4:**
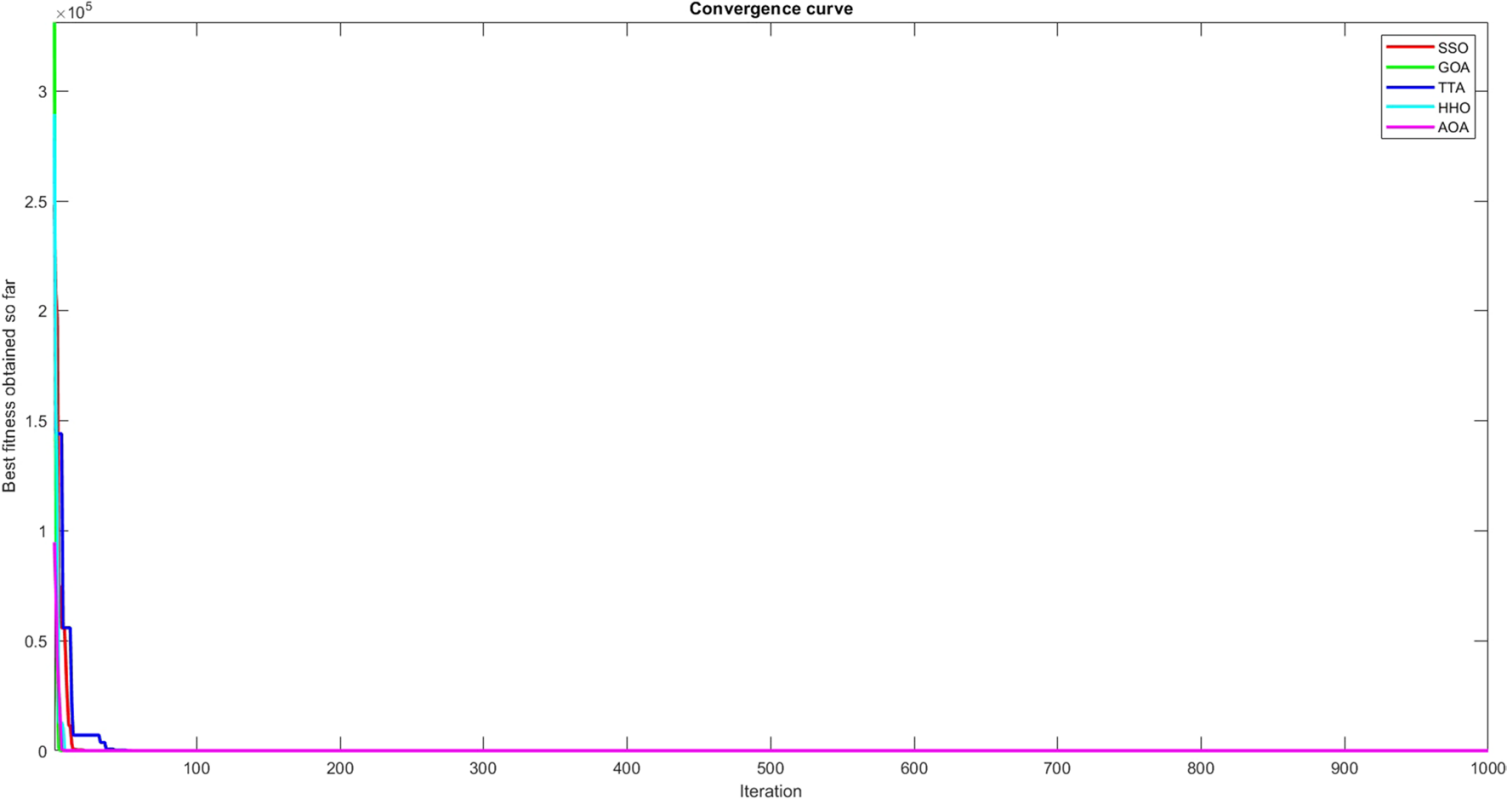
Mathematical test function 3.

**Figure 5:**
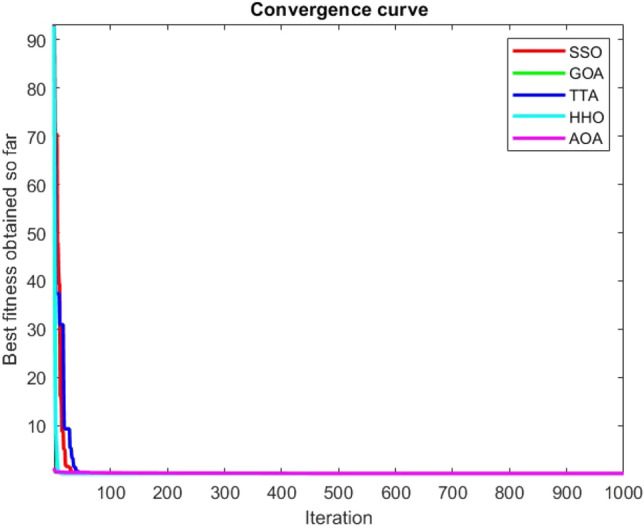
Mathematical test function 4.

**Figure 6:**
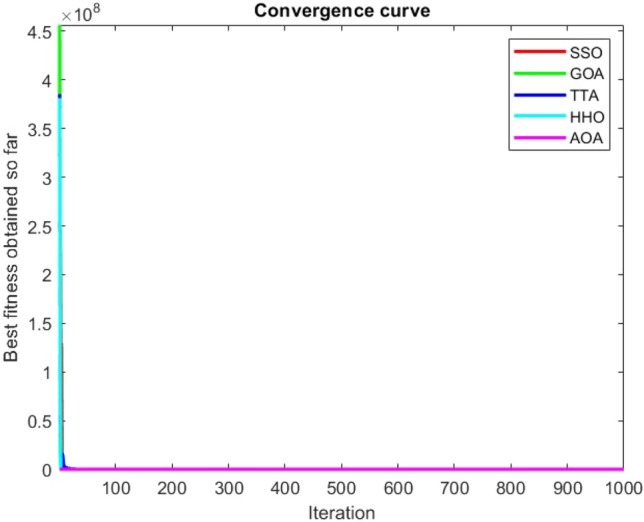
Mathematical test function 5.

**Figure 7:**
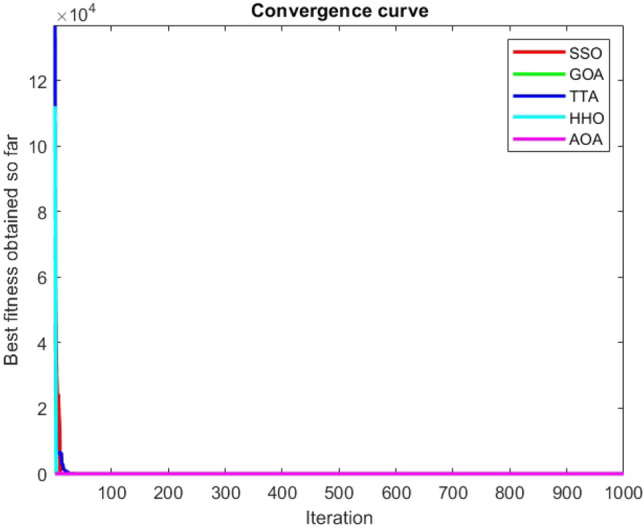
Mathematical test function 6.

**Figure 8:**
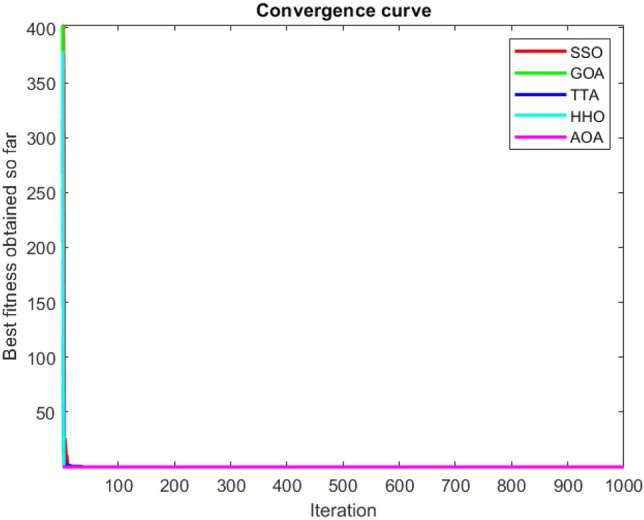
Mathematical test function 7.

**Figure 9:**
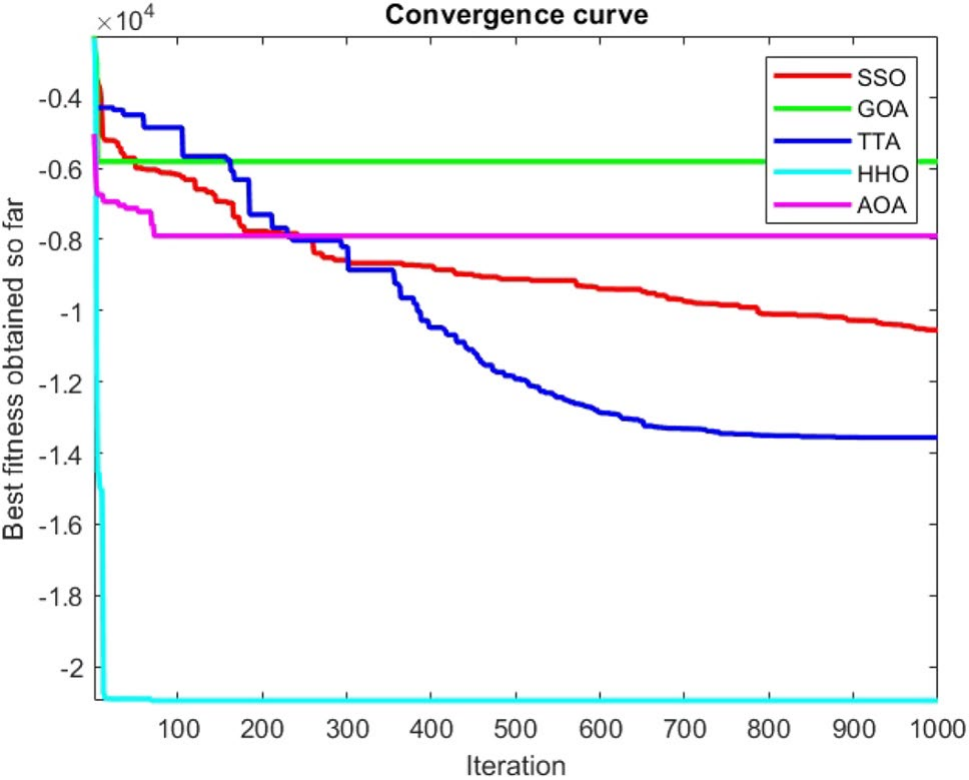
Mathematical test function 8.

**Figure 10:**
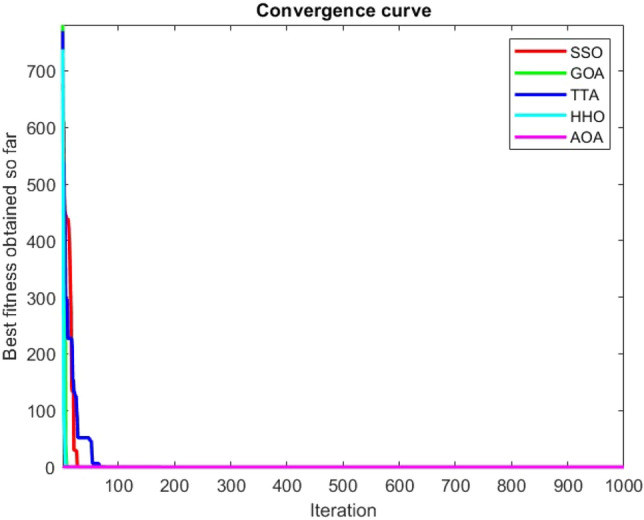
Mathematical test function 9.

**Figure 11:**
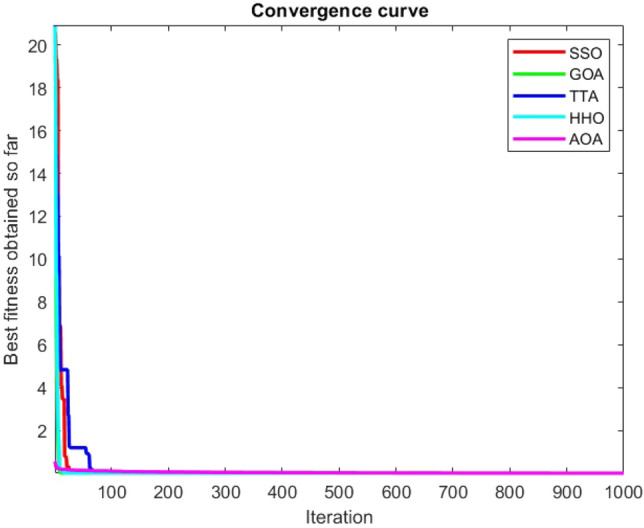
Mathematical test function 10.

**Figure 12:**
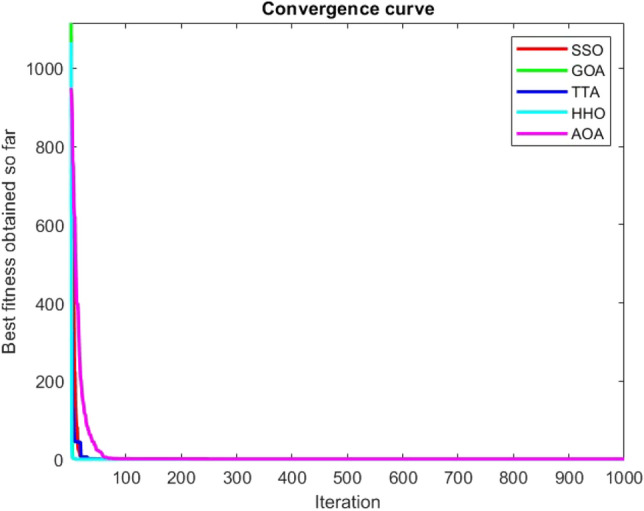
Mathematical test function 11.

**Figure 13:**
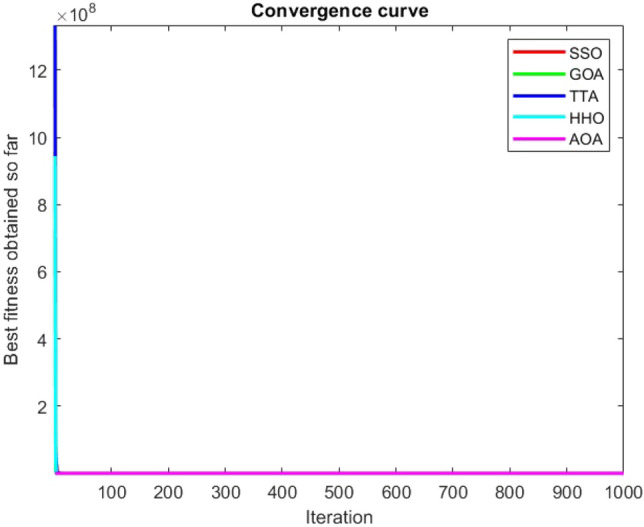
Mathematical test function 12.

**Figure 14:**
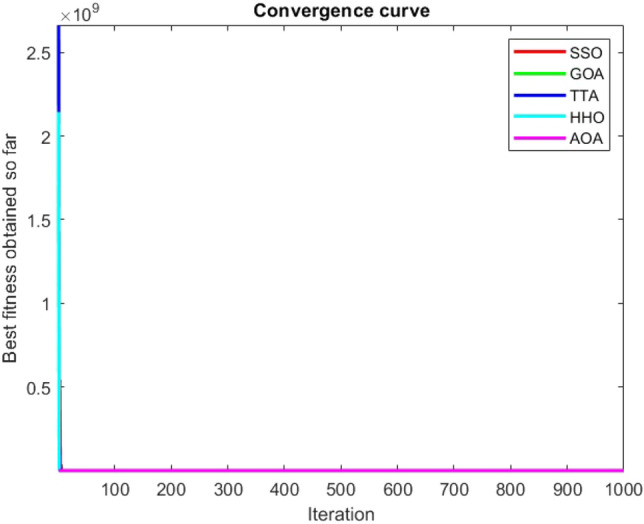
Mathematical test function 13.

**Figure 15:**
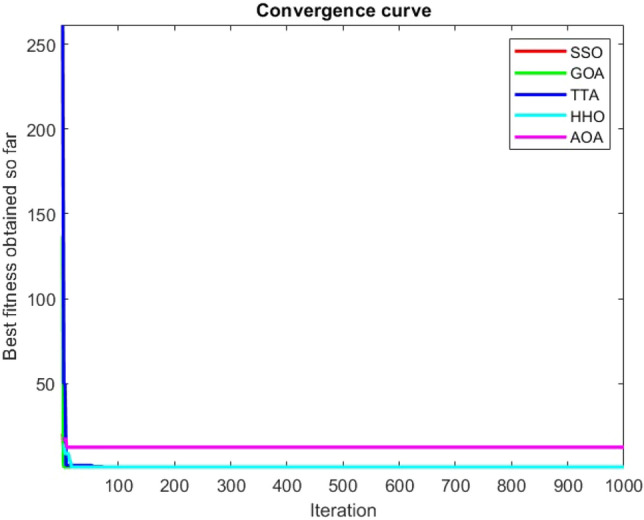
Mathematical test function 14.

**Figure 16:**
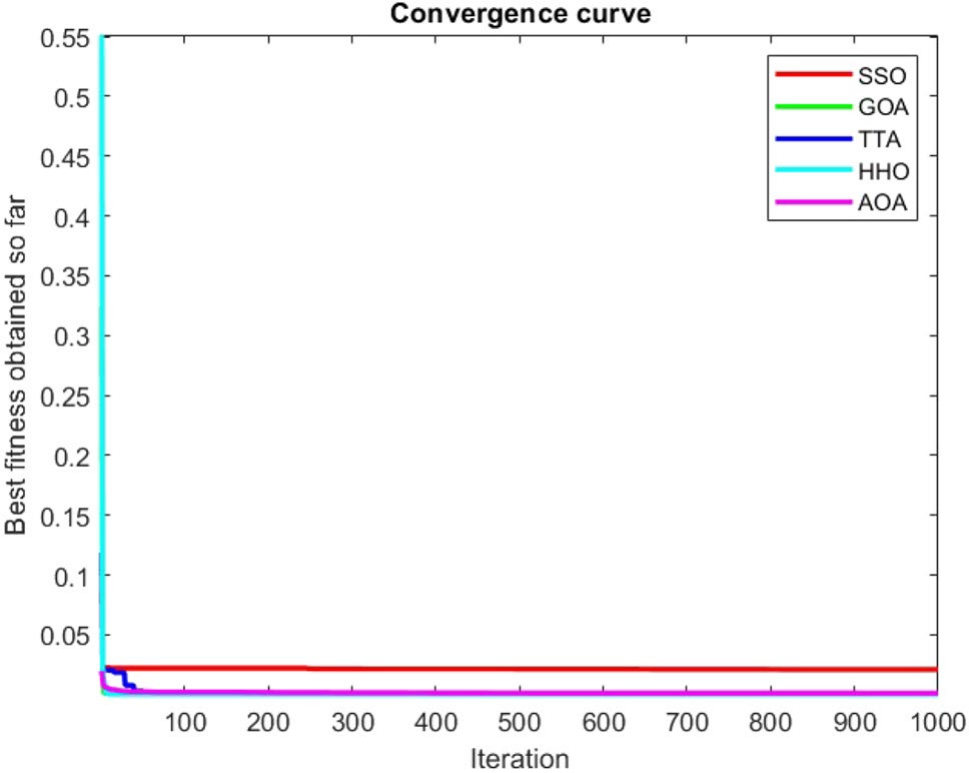
Mathematical test function 15.

**Figure 17:**
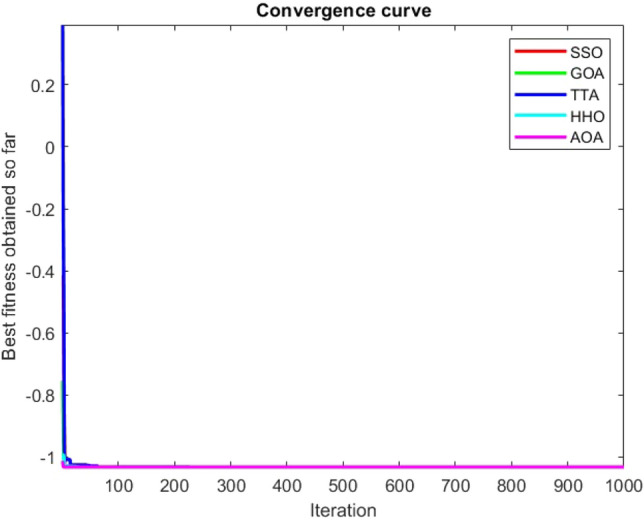
Mathematical test function 16.

**Figure 18:**
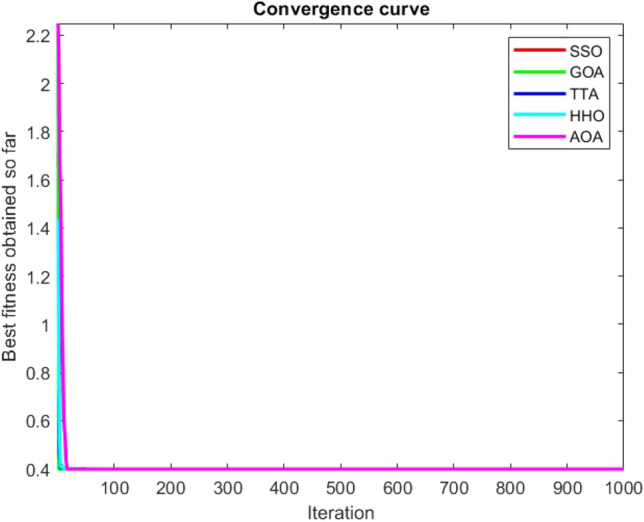
Mathematical test function 17.

**Figure 19:**
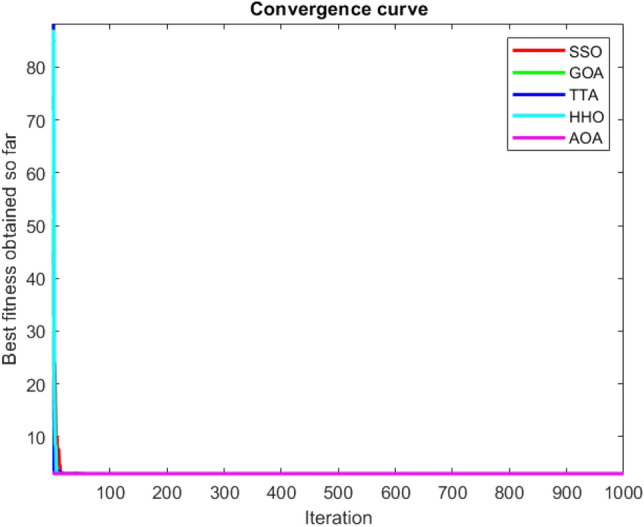
Mathematical test function 18.

**Figure 20:**
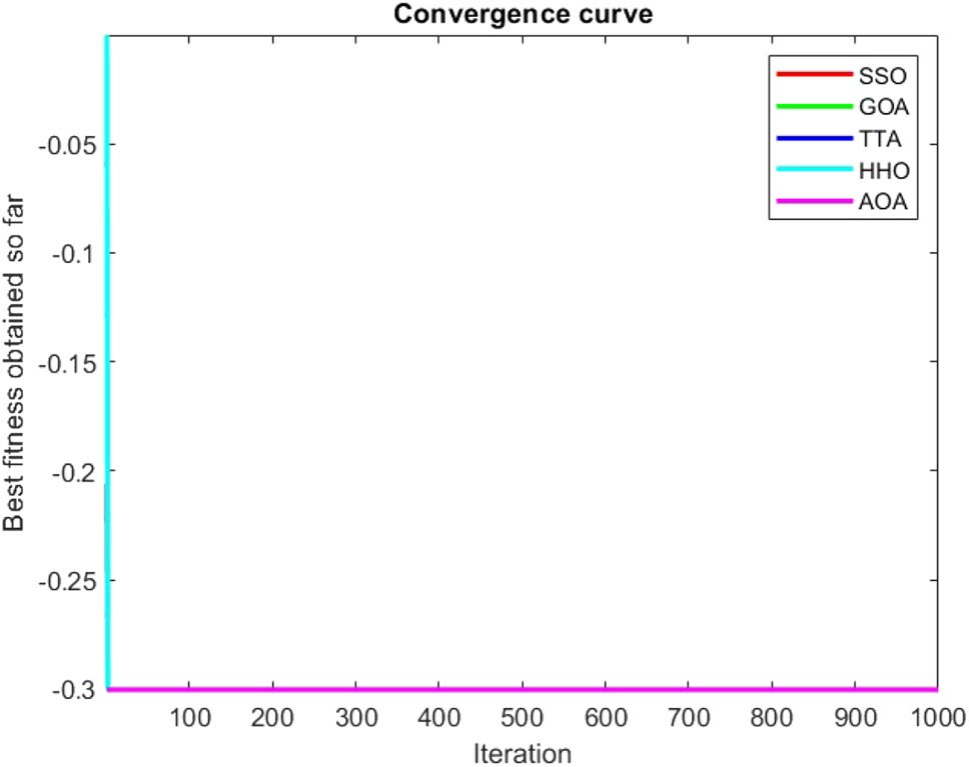
Mathematical test function 19.

**Figure 21:**
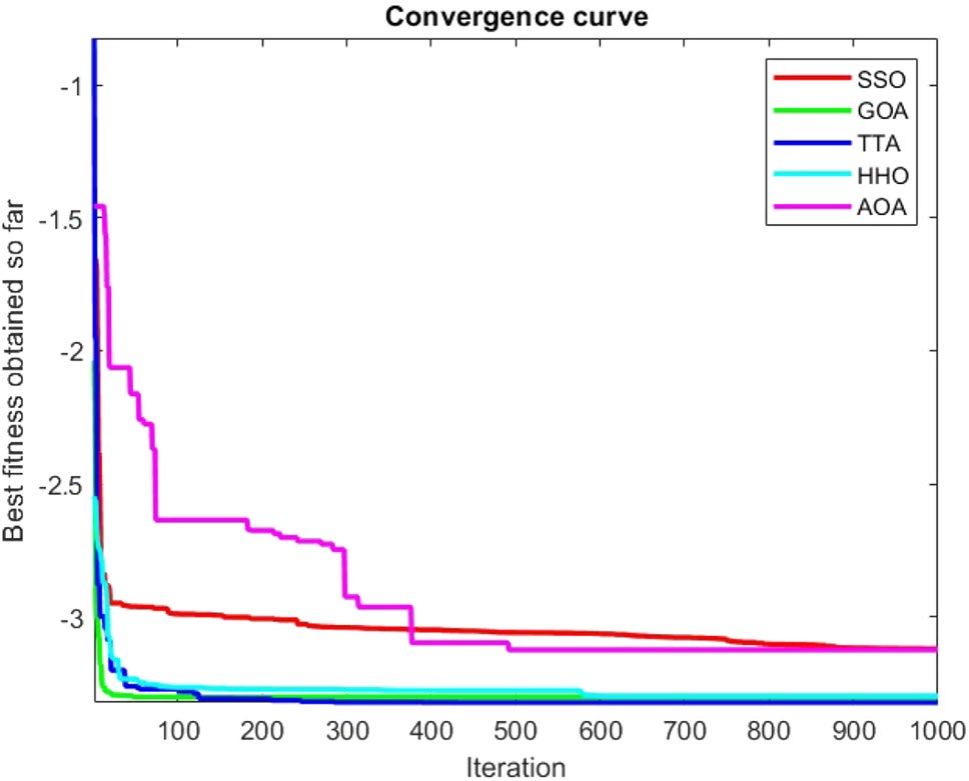
Mathematical test function 20.

**Figure 22:**
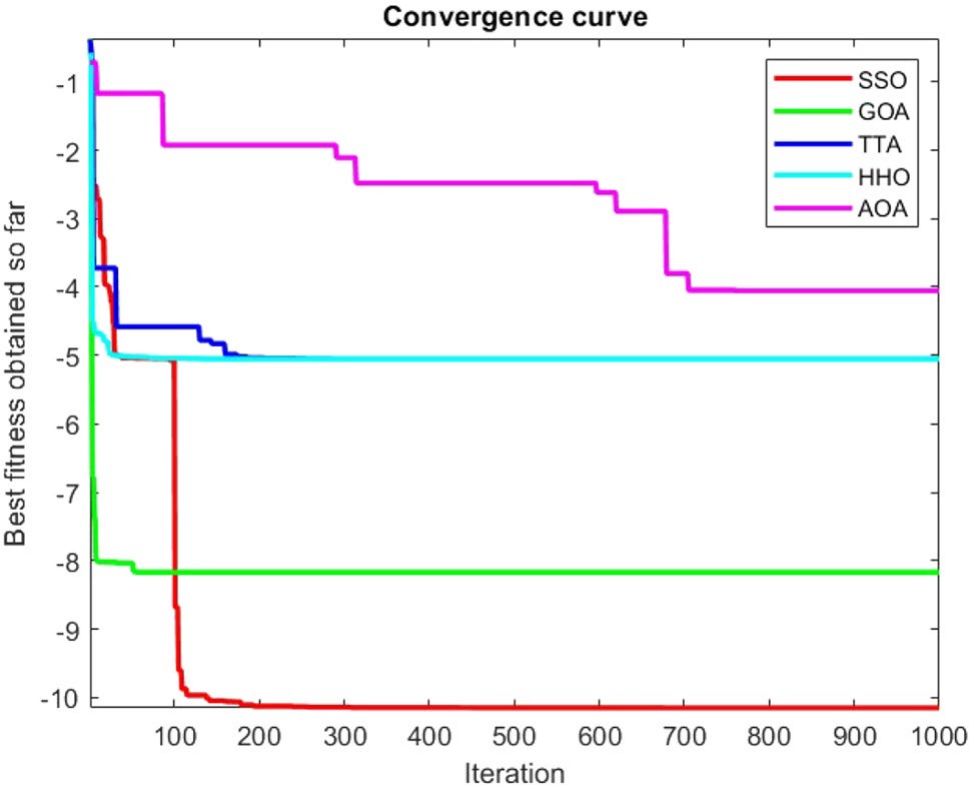
Mathematical test function 21.

**Figure 23:**
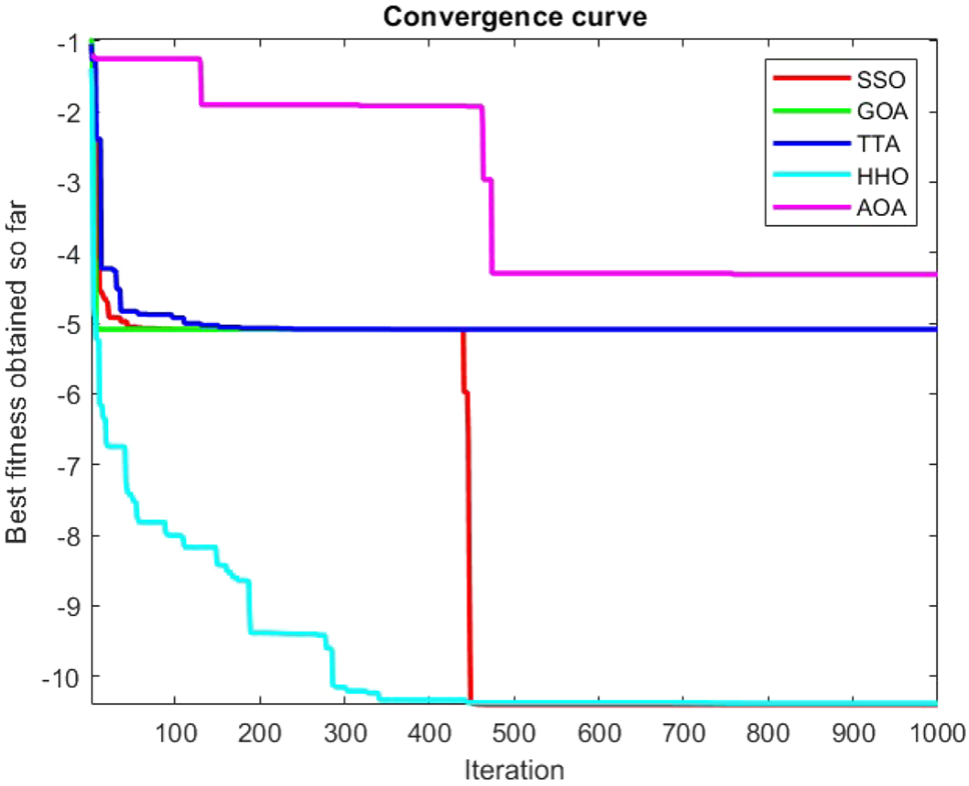
Mathematical test function 22.

**Figure 24:**
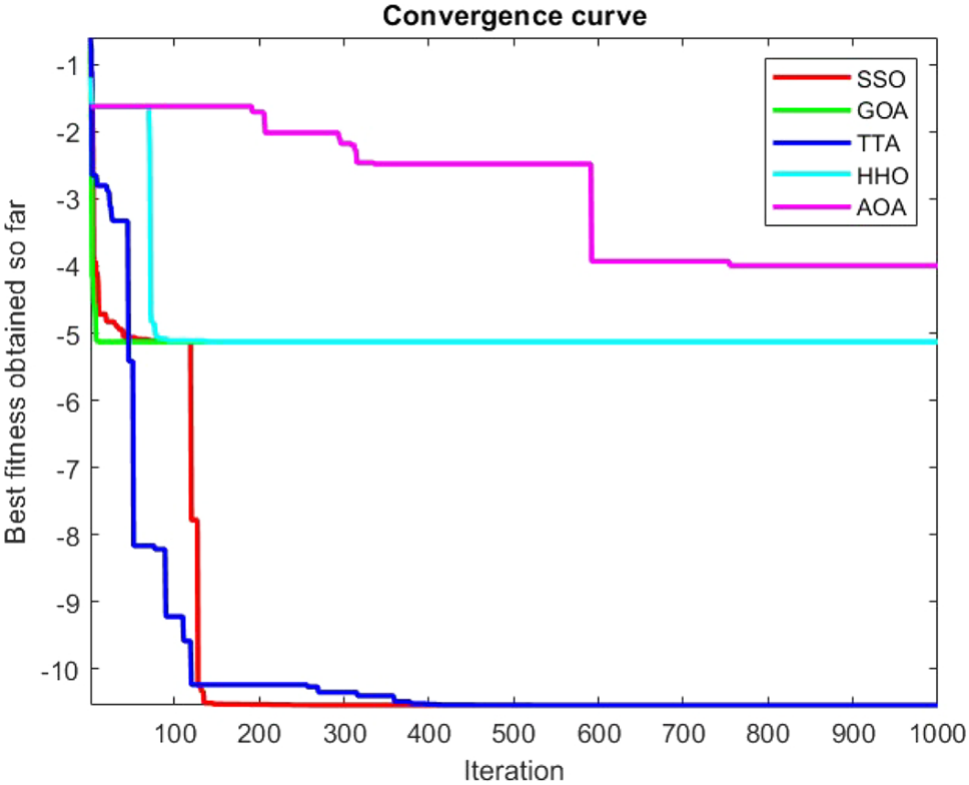
Mathematical test function 23.

### Analysis of exploitation ability of SSO

Functions F1-F7 are unimodal functions and therefore they have only one global optimum solution in their search space. This can be a test for assessment of the exploitation ability of the proposed algorithm. The results for F1-F7 mathematical test functions in Tables 2 and 3 show that the SSO algorithm can perform exploitation in a relatively good way and very close to the performance of the compared algorithms. This indicates that the ability of the SSO algorithm in converging towards the optimum solution is quite accurate and fast due to the correct choosing of controlling parameters for the trade-off between exploration and exploitation of the proposed algorithm. It can also be inferred that selecting random solutions alongside with the best so far found solution, leads to a robust exploitation capability of the SSO algorithm. Furthermore, the RND vector defined in the algorithm can boost up the convergence of the SSO to guide the search agents towards the optimum solutions. It can also be seen that the GOA algorithm is a close rival for the SSO algorithm according to the results obtained and stipulated in the Table 2, which we can understand the similarity between both the SSO and GOA algorithms in adopting a search strategy for conducting a better exploitation in the search space of different optimization problems.

**Table 2:** Mean of the results for mathematical test functions.

NO	SSO	GOA	TTA	HHO	AOA
F1	**0.00E + 00**	0.00E + 00	0.00E + 00	0.00E + 00	0.00E + 00
F2	**0.00E + 00**	0.00E + 00	0.00E + 00	0.00E + 00	0.00E + 00
F3	**0.00E + 00**	0.00E + 00	0.00E + 00	0.00E + 00	0.0508
F4	**0.00E + 00**	0.00E + 00	0.00E + 00	0.00E + 00	0.0457
F5	48.4343	0.00E + 00	48.2212	0.0028	48.4952
F6	0.0200	0.00E + 00	4.7406	1.76E-06	6.2827
F7	4.0139E-04	1.20E-05	9.41E-05	4.69E-05	1.84E-05
F8	-1.1680E + 04	-5.38E + 03	-1.19E + 04	-2.09E + 04	-7.99E + 03
F9	**0.00E + 00**	0.00E + 00	0.00E + 00	0.00E + 00	0.00E + 00
F10	**0.00E + 00**	0.00E + 00	0.00E + 00	0.00E + 00	0.00E + 00
F11	**0.00E + 00**	0.00E + 00	0.00E + 00	0.00E + 00	0.4525
F12	0.0037	0.00E + 00	0.0759	5.48E-07	0.5869
F13	3.5644	0.00E + 00	3.4569	8.96E-06	4.7963
F14	1.4608	1.5002	1.00E + 00	1.1955	9.0192
F15	5.15E-04	0.0011	6.58E-04	3.22E-04	0.0061
F16	**-1.0316**	-1.0316	-1.0316	-1.0316	-1.0316
F17	**0.398**	0.398	0.398	0.398	0.398
F18	**3.00E + 00**	3.11E + 00	3.00E + 00	3.00E + 00	3.00E + 00
F19	**-0.3005**	-0.3005	-0.3005	-0.3005	-0.3005
F20	**-3.2701**	-3.09E + 00	-3.2586	-3.1985	-3.1323
F21	**-8.9637**	-7.99E + 00	-6.5873	-5.5628	-4.3350
F22	**-9.3399**	-6.9150	-9.2944	-5.4409	-4.7512
F23	-9.4117	-7.5264	-9.8185	-5.4872	-4.1004

The results that have advantage over the other methods are bolded and underlined.

**Table 3:** Standard deviation of the results for mathematical test functions.

NO	SSO	GOA	TTA	HHO	AOA
F1	**0.00E + 00**	0.00E + 00	0.00E + 00	0.00E + 00	0.00E + 00
F2	**0.00E + 00**	0.00E + 00	0.00E + 00	0.00E + 00	0.00E + 00
F3	**0.00E + 00**	0.00E + 00	0.00E + 00	0.00E + 00	0.0664
F4	**0.00E + 00**	0.00E + 00	0.00E + 00	0.00E + 00	0.0096
F5	0.0274	0.00E + 00	0.6776	0.0034	0.3863
F6	0.0047	0.00E + 00	0.7379	2.45E-06	0.5833
F7	3.3220E-04	9.97E-06	1.07E-04	4.39E-05	1.81E-05
F8	694.5889	948.7218	2.91E + 03	0.2562	635.7357
F9	**0.00E + 00**	0.00E + 00	0.00E + 00	0.00E + 00	0.00E + 00
F10	**0.00E + 00**	0.00E + 00	0.00E + 00	0.00E + 00	0.00E + 00
F11	**0.00E + 00**	0.00E + 00	0.00E + 00	0.00E + 00	0.1682
F12	0.0033	0.00E + 00	0.0336	8.34E-07	0.0428
F13	1.9422	0.00E + 00	0.4344	1.31E-05	0.0938
F14	0.8914	1.0921	1.39E-16	0.9122	3.8416
F15	3.97E-04	0.0041	1.92E-04	1.40E-05	0.0089
F16	1.08E-10	2.69E-05	6.07E-16	2.36E-12	6.15E-08
F17	4.63E-10	0.0038	0.00E + 00	2.44E-08	2.53E-08
F18	7.47E-09	0.4655	2.05E-10	7.73E-09	12.7147
F19	**2.25E-16**	2.25E-16	2.25E-16	2.25E-16	2.25E-16
F20	0.0763	0.1882	0.0603	0.0578	0.0494
F21	2.1931	2.0781	2.3744	1.5494	1.0723
F22	2.1625	2.3284	2.2667	1.3451	1.5472
F23	2.2983	2.2873	1.8616	1.3659	1.5530

The results that have advantage over the other methods are bolded and underlined.

### Analysis of exploration ability of SSO

Since the multimodal mathematical test functions (F8-F23) have many local optima, and the local optima of these functions increases with expanding their dimensions, it can be very useful to test the exploration ability of the proposed method with evaluating these test functions. Moreover, evaluating the test functions with low dimensions is of great importance. The SSO shows to be a relatively good method in dealing with the test functions for checking exploration capability of itself and provides results that makes it a good algorithm to compete with excellent high performance optimization algorithms (Tables 4, 5, 6, 7, 8, 9, 10, 11).

**Table 4:** Mean of the results for CEC-BC-2017(with dimensions = 10).

NO	SSO	HHO	CMA-ES	EBOwithCMAR	AOA
F1	5.06E + 03	1.58E + 05	2.52E + 03	100.0000	9.37E + 08
F3	300.0142	300.5068	300.0000	300.0000	2.12E + 03
F4	407.5092	409.8126	400.0000	400.0000	488.8725
F5	513.7650	535.8798	555.8831	500.0000	543.8242
F6	608.2479	619.6248	631.2500	600.0000	634.6448
F7	728.7065	771.2006	871.9100	710.5500	796.5826
F8	816.3509	822.8548	850.2100	800.0000	830.9376
F9	907.3487	1.28E + 03	2.28E + 03	900.0000	1.34E + 03
F10	1.67E + 03	1.93E + 03	2.02E + 03	1.05E + 03	1.90E + 03
F11	1.16E + 03	1.16E + 03	1.11E + 03	1100.00	1.13E + 03
F12	1.2E + 06	3.19E + 05	1.65E + 03	1.28E + 03	9.20E + 05
F13	6.66E + 03	1.51E + 04	1.37E + 03	1.30E + 03	9.19E + 03
F14	1.47E + 03	1.50E + 03	1.51E + 03	1.40E + 03	6.91E + 03
F15	2.03E + 03	1.62E + 03	1.55E + 03	1.50E + 03	9.43E + 03
F16	1.66E + 03	1.82E + 03	2.04E + 03	1.60E + 03	1.97E + 03
F17	1.74E + 03	1.77E + 03	1.93E + 03	1.70E + 03	1.83E + 03
F18	9.13E + 03	1.64E + 04	1.86E + 03	1.80E + 03	1.49E + 04
F19	2.21E + 03	8.94E + 03	1.93E + 03	1.90E + 03	1.76E + 04
F20	2.05E + 03	2.10E + 03	2.18E + 03	2.00E + 03	2.10E + 03
F21	**2.22E + 03**	2.29E + 03	2.35E + 03	2.22E + 03	2.28E + 03
F22	**2.29E + 03**	2.30E + 03	2.25E + 03	2.30E + 03	2.52E + 03
F23	2.61E + 03	2.65E + 03	2.65E + 03	2.60E + 03	2.68E + 03
F24	2.68E + 03	2.76E + 03	2.63E + 03	2.55E + 03	2.80E + 03
F25	2.93E + 03	2.92E + 03	2.93E + 03	2.91E + 03	2.96E + 03
F26	2.91E + 03	3.17E + 03	3.04E + 03	2.88E + 03	3.45E + 03
F27	**3.09E + 03**	3.12E + 03	3.10E + 03	3.09E + 03	3.18E + 03
F28	3.24E + 03	3.28E + 03	3.37E + 03	3.10E + 03	3.45E + 03
F29	3.17E + 03	3.27E + 03	3.36E + 03	3.13E + 03	3.27E + 03
F30	4.75E + 05	4.07E + 05	1.39E + 05	3.40E + 03	2.21E + 06

The results that have advantage over the other methods are bolded and underlined.

**Table 5:** Standard deviation of the results for CEC-BC-2017(with dimensions = 10).

NO	SSO	HHO	CMA-ES	EBOwithCMAR	AOA
F1	3.42E + 03	8.79E + 04	0.00E + 00	0.00E + 00	1.53E + 03
F3	0.0105	0.2402	0.00E + 00	0.00E + 00	82.0048
F4	12.6379	20.5411	0.00E + 00	0.00E + 00	45.7754
F5	5.6105	12.3856	31.47E + 00	1.44E-12	14.9006
F6	6.8835	11.4189	18.48E + 00	0.00E + 00	5.6128
F7	7.7239	18.1366	189.97E + 00	0.1745	14.6178
F8	6.0756	9.8742	23.56E + 00	1.03–12	7.1056
F9	34.3035	223.4491	483.28E + 00	0.00E + 00	177.0636
F10	233.8491	244.6249	363.54E + 00	65.0612	188.9749
F11	52.3310	60.9392	11.49E + 00	2.53E-13	18.7059
F12	1.72E + 06	5.86E + 05	209.9873	64.7312	1.22E + 06
F13	6.33E + 03	1.00E + 04	165.9266	2.6301	6.34E + 03
F14	37.6326	22.2691	110.7835	3.6798	6.63E + 03
F15	435.6148	76.1709	47.0618	0.2152	5.56E + 03
F16	57.0930	126.9841	184.2659	0.1848	137.2655
F17	19.9186	38.5289	160.6022	1.30E-05	87.8757
F18	9.20E + 03	1.22E + 04	56.4829	3.6805	9.95E + 03
F19	567.1635	6.30E + 03	23.9616	9.28E-03	1.60E + 04
F20	20.2623	65.5467	58.2672	0.1343	50.6827
F21	44.7793	64.4078	33.9534	41.2604	50.8711
F22	23.4801	20.8449	47.5529	0.00E + 00	106.7917
F23	8.1919	20.3992	167.2058	7.65E-07	21.7041
F24	105.6776	93.7854	117.0648	87.6376	45.4453
F25	28.4166	63.7865	14.2427	22.2058	47.7866
F26	**42.6672**	306.4397	487.8637	61.0257	286.9078
F27	4.6108	34.7980	35.5876	2.4617	26.7476
F28	112.1875	113.5687	107.8111	51.8046	169.0917
F29	24.5532	61.2740	166.0766	1.9257	83.8795
F30	6.69E + 05	6.48E + 05	3.09E + 05	20.9621	2.48E + 06

The results that have advantage over the other methods are bolded and underlined.

**Table 6:** Mean of the results for CEC-BC-2017(with dimensions = 30).

NO	SSO	HHO	CMA-ES	EBOwithCMAR	AOA
F1	1.30E + 06	1.04E + 07	100.00	100.00	4.39E + 10
F3	1.15E + 04	6.46E + 03	300.00	300.00	7.55E + 04
F4	527.3598	536.4367	456.9586	451.2304	9.11E + 03
F5	643.8462	717.6747	683.3684	505.4860	8.32E + 02
F6	640.8229	660.1770	661.7798	600.00	667.0330
F7	940.4008	1.25E + 03	1.00E + 03	735.4208	1.31E + 03
F8	916.0751	962.2834	976.5701	804.9874	1.06E + 03
F9	2.91E + 03	6.28E + 03	9.76E + 03	900.0211	6.11E + 03
F10	4.93E + 03	5.38E + 03	5.32E + 03	2.62E + 03	6.94E + 03
F11	1.32E + 03	1.24E + 03	1.27E + 03	1.12E + 03	4.91E + 03
F12	4.42E + 07	1.13E + 07	5.13E + 03	2.78E + 03	8.43E + 09
F13	1.23E + 05	2.82E + 05	2.83E + 04	1.34E + 03	5.96E + 08
F14	5.03E + 04	6.81E + 04	1.58E + 03	1.43E + 03	1.67E + 05
F15	4.65E + 04	5.73E + 04	2.84E + 03	1.53E + 03	2.66E + 04
F16	2.86E + 03	3.18E + 03	1.88E + 03	1.79E + 03	3.82E + 03
F17	2.18E + 03	2.50E + 03	1.94E + 03	1.74E + 03	2.68E + 03
F18	5.53E + 05	1.14E + 06	2.27E + 03	1.87E + 03	3.05E + 06
F19	2.31E + 06	2.24E + 05	2.14E + 03	1.92E + 03	1.36E + 06
F20	2.50E + 03	2.71E + 03	2.92E + 03	2.07E + 03	2.71E + 03
F21	2.43E + 03	2.53E + 03	2.46E + 03	2.30E + 03	2.62E + 03
F22	2.92E + 03	5.79E + 03	6.59E + 03	2.30E + 03	8.08E + 03
F23	2.86E + 03	3.06E + 03	2.70E + 03	2.65E + 03	3.32E + 03
F24	3.02E + 03	3.30E + 03	2.83E + 03	2.80E + 03	3.67E + 03
F25	2.93E + 03	2.91E + 03	2.88E + 03	2.88E + 03	4.61E + 03
F26	5.60E + 03	7.18E + 03	3.36E + 03	3.19E + 03	9.18E + 03
F27	3.32E + 03	3.32E + 03	3.21E + 03	3.20E + 03	4.20E + 03
F28	3.30E + 03	3.27E + 03	3.15E + 03	3.11E + 03	6.05E + 03
F29	4.31E + 03	4.32E + 03	3.54E + 03	3.34E + 03	6.04E + 03
F30	1.08E + 07	1.48E + 06	8.48E + 03	5.21E + 03	1.16E + 08

**Table 7:** Standard deviation of the results for CEC-BC-2017(with dimensions = 30).

NO	SSO	HHO	CMA-ES	EBOwithCMAR	AOA
F1	3.85E + 05	1.80E + 06	7.46E-15	1.292E-13	7.46E + 09
F3	3.74E + 03	2.00E + 03	25.85E-15	6.14E-08	8.91E + 03
F4	39.0502	42.5976	9.8810	23.1711	2.64E + 03
F5	30.4218	29.2311	180.3096	2.0691	31.3881
F6	5.1342	6.6641	10.4171	1.81E-06	6.4399
F7	44.0442	52.20	695.7159	1.3629	46.6983
F8	24.7085	21.5646	153.8865	2.0805	27.4446
F9	616.1883	7.26E + 02	1.69E + 03	0.0881	1.06E + 03
F10	672.9247	7.15E + 02	916.37	213.5509	527.4837
F11	57.8865	46.4606	58.1149	25.6885	1.99E + 03
F12	3.47E + 07	7.23E + 06	2.21E + 03	1.36E + 03	2.35E + 09
F13	1.08E + 05	2.78E + 05	6.88E + 04	23.7628	1.04E + 09
F14	6.11E + 04	9.21E + 04	43.5302	10.8438	1.84E + 05
F15	3.86E + 04	4.35E + 04	603.5139	18.3586	1.28E + 04
F16	2.99E + 02	369.2356	1.88E + 03	141.4801	608.3084
F17	1.93E + 02	286.5457	206.9133	8.6494	274.6835
F18	4.44E + 05	1.19E + 06	172.5212	40.5837	3.75E + 06
F19	1.48E + 06	1.84E + 05	78.0098	14.9304	9.83E + 04
F20	1.35E + 02	1.69 + 02	277.6799	53.7460	180.7018
F21	27.27	48.39	131.3866	19.4971	37.8955
F22	1.52E + 03	2.06E + 03	1.56E + 03	3.58E-13	1.00E + 03
F23	48.05	86.1860	203.8233	5.6023	136.1689
F24	63.14	88.4511	5.7857	71.4372	131.4629
F25	23.92	20.13	31.78E-03	0.5769	546.5345
F26	8.99E + 02	992.0004	443.1338	381.1415	834.0049
F27	77.35	56.9242	10.0225	7.3075	246.9755
F28	35.31	29.0095	63.8688	35.7106	655.5168
F29	2.80E + 02	308.7964	135.9257	16.5146	784.2082
F30	8.79E + 06	7.69E + 05	1.39E + 03	146.5684	2.24E + 08

**Table 8:** Mean of the results for CEC-BC-2017(with dimensions = 50).

NO	SSO	HHO	CMA-ES	EBOwithCMAR	AOA
F1	4.17E + 07	5.86E + 07	100.0000	100.0000	1.01E + 11
F3	1.12E + 05	3.49E + 04	345.9444	300.0000	1.66E + 05
F4	723.7271	675.6358	465.3239	443.5542	2.82E + 04
F5	790.0499	879.6405	961.3627	522.3243	1.12E + 03
F6	653.5269	671.0650	669.2858	600.0026	690.8535
F7	1.29E + 03	1.78E + 03	1.22E + 03	767.3672	1.90E + 03
F8	1.11E + 03	1.16E + 03	1.25E + 03	821.5586	1.45E + 03
F9	1.08E + 04	2.04E + 04	2.01E + 04	903.1710	2.50E + 04
F10	8.57E + 03	9.15E + 03	7.74E + 03	4.38E + 03	1.27E + 04
F11	1.85E + 03	1.46E + 03	1.35E + 03	1.21E + 03	1.97E + 04
F12	1.95E + 08	9.42E + 07	2.55E + 05	4.85E + 03	5.86E + 10
F13	2.14E + 05	2.49E + 06	1.06E + 07	1.93E + 03	2.13E + 10
F14	5.35E + 05	6.24E + 05	3.14E + 03	1.61E + 03	8.78E + 06
F15	6.26E + 04	3.31E + 05	4.28E + 04	1.89E + 03	2.63E + 08
F16	3.97E + 03	4.25E + 03	2.04E + 03	2.09E + 03	6.57E + 03
F17	3.29E + 03	3.57E + 03	2.29E + 03	2.17E + 03	5.44E + 03
F18	3.79E + 06	3.21E + 06	6.83E + 04	1.96E + 03	4.82E + 07
F19	3.98E + 06	6.82E + 05	2.44E + 04	2.03E + 03	2.72E + 08
F20	3.11E + 03	3.47E + 03	3.99E + 03	2.35E + 03	3.54E + 03
F21	2.61E + 03	2.80E + 03	2.54E + 03	2.32E + 03	3.02E + 03
F22	1.00E + 04	1.10E + 04	9.43E + 03	4.40E + 03	1.52E + 04
F23	3.28E + 03	3.69E + 03	2.77E + 03	2.75E + 03	4.25E + 03
F24	3.42E + 03	4.07E + 03	2.93E + 03	2.92E + 03	4.70E + 03
F25	3.21E + 03	3.16E + 03	2.98E + 03	3.02E + 03	1.45E + 04
F26	8.90E + 03	1.04E + 04	3.96E + 03	3.38E + 03	1.61E + 04
F27	3.89E + 03	4.15E + 03	3.27E + 03	3.28E + 03	6.21E + 03
F28	3.53E + 03	3.45E + 03	3.27E + 03	3.28E + 03	1.14E + 04
F29	6.65E + 03	5.65E + 03	3.52E + 03	3.33E + 03	2.00E + 04
F30	1.60E + 08	2.43E + 07	2.43E + 06	6.87E + 05	1.76E + 09

**Table 9:** Standard deviation of the results for CEC-BC-2017(with dimensions = 50).

NO	SSO	HHO	CMA-ES	EBOwithCMAR	AOA
F1	1.32E + 07	1.25E + 07	1.11E-14	1.00E-04	1.16E + 10
F3	1.43E + 04	7.52E + 03	92.0445	2.95E-07	2.30E + 04
F4	57.7004	63.2285	53.5603	46.1186	5.27E + 03
F5	42.1635	28.7490	279.0454	4.9134	46.9626
F6	7.4299	4.5572	7.1540	0.0056	6.0062
F7	132.9568	97.9441	1.31E + 03	2.9594	46.9958
F8	45.2586	33.1527	364.1836	3.9385	43.8940
F9	1.61E + 03	2.84E + 03	2.98E + 03	4.6208	2.88E + 03
F10	1.00E + 03	1.20E + 03	820.0595	393.6809	859.5990
F11	161.0013	121.0273	49.6849	33.3225	3.24E + 03
F12	1.31E + 08	6.71E + 07	1.57E + 05	6.13E + 03	1.16E + 10
F13	1.34E + 05	1.61E + 06	4.21E + 07	435.3434	8.10E + 09
F14	4.23E + 05	3.82E + 05	1.41E + 03	55.4384	1.02E + 07
F15	4.86E + 04	2.94E + 05	1.76E + 04	107.8179	3.39E + 08
F16	472.5677	553.8779	223.1987	199.9461	1.05E + 03
F17	287.3663	316.6430	199.3778	145.1708	1.02E + 03
F18	3.15E + 06	2.51E + 06	2.53E + 04	104.4727	2.98E + 07
F19	3.95E + 06	4.26E + 05	1.22E + 04	43.0771	3.53E + 08
F20	289.0653	267.7901	348.8282	164.2410	292.7109
F21	59.8620	83.6983	268.3300	5.5247	71.2523
F22	1.08E + 03	1.07E + 03	1.21E + 03	2.03E + 03	642.1088
F23	111.3962	144.1249	15.3981	9.6930	213.4664
F24	102.6759	194.4515	9.6964	5.8799	249.4549
F25	55.0692	40.2910	19.1674	40.7629	1.44E + 03
F26	1.81E + 03	1.56E + 03	381.7298	532.0457	977.6227
F27	247.8071	378.7915	32.8215	39.9613	496.3528
F28	66.7596	62.4696	22.7667	21.8083	1.20E + 03
F29	847.1558	385.0463	155.3217	80.1201	7.50E + 03
F30	4.41E + 07	5.02E + 06	5.51E + 05	9.47E + 04	1.81E + 09

**Table 10:** Mean of the results for CEC-BC-2017(with dimensions = 100).

NO	SSO	HHO	CMA-ES	EBOwithCMAR	AOA
F1	2.77E + 09	5.98E + 08	5.70E + 03	220.0647	2.63E + 11
F3	2.94E + 05	2.00E + 05	3.00E + 05	300.0001	3.30E + 05
F4	1.58E + 03	1.18E + 03	619.2041	447.1920	8.35E + 04
F5	1.32E + 03	1.47E + 03	1.83E + 03	580.4258	2.01E + 03
F6	668.4889	681.6596	677.5415	600.0691	705.7987
F7	2.60E + 03	3.65E + 03	3.18E + 03	869.1997	3.86E + 03
F8	1.77E + 03	1.96E + 03	2.33E + 03	876.2138	2.47E + 03
F9	3.21E + 04	4.71E + 04	4.13E + 04	928.8395	6.25E + 04
F10	1.92E + 04	2.13E + 04	1.46E + 04	1.24E + 04	2.94E + 04
F11	7.02E + 04	6.51E + 03	2.65E + 03	2.03E + 03	1.66E + 05
F12	1.52E + 09	5.79E + 08	7.42E + 06	4.25E + 05	1.81E + 11
F13	5.78E + 05	6.47E + 06	8.32E + 04	2.21E + 03	3.97E + 10
F14	5.20E + 06	2.29E + 06	2.86E + 05	1.89E + 03	5.02E + 07
F15	5.85E + 05	1.94E + 06	9.66E + 04	1.81E + 03	1.59E + 10
F16	8.49E + 03	7.72E + 03	3.23E + 03	3.82E + 03	1.81E + 04
F17	5.79E + 03	6.43E + 03	2.93E + 03	3.72E + 03	1.94E + 06
F18	4.14E + 06	3.95E + 06	5.81E + 05	2.15E + 03	9.90E + 07
F19	2.21E + 07	7.82E + 06	8.99E + 05	2.29E + 03	1.65E + 10
F20	5.34E + 03	5.89E + 03	5.98E + 03	4.00E + 03	6.62E + 03
F21	3.45E + 03	3.99E + 03	2.90E + 03	2.40E + 03	4.58E + 03
F22	2.19E + 04	2.41E + 04	1.75E + 04	1.45E + 04	3.23E + 04
F23	4.38E + 03	4.90E + 03	3.07E + 03	2.93E + 03	6.79E + 03
F24	5.72E + 03	6.51E + 03	3.38E + 03	3.39E + 03	1.05E + 04
F25	4.26E + 03	3.82E + 03	3.20E + 03	3.24E + 03	2.73E + 04
F26	2.50E + 04	2.68E + 04	6.62E + 03	6.09E + 03	5.16E + 04
F27	4.87E + 03	4.56E + 03	3.35E + 03	3.37E + 03	1.19E + 04
F28	4.77E + 03	3.90E + 03	3.35E + 03	3.32E + 03	3.23E + 04
F29	1.17E + 04	9.87E + 03	5.00E + 03	4.83E + 03	2.51E + 05
F30	4.87E + 08	5.07E + 07	4.46E + 06	8.17E + 03	3.49E + 10

**Table 11:** Standard deviation of the results for CEC-BC-2017(with dimensions = 100).

NO	SSO	HHO	CMA-ES	EBOwithCMAR	AOA
F1	5.12E + 08	8.08E + 07	7.96E + 03	425.0885	1.39E + 10
F3	2.25E + 04	2.04E + 04	5.68E + 04	1.65E-05	1.69E + 04
F4	177.5856	95.8223	15.2142	74.0727	1.39E + 04
F5	59.9579	45.5598	278.6471	10.5817	53.3052
F6	4.7832	3.6782	4.9061	0.0426	4.6622
F7	197.4012	126.8195	4.17E + 03	10.8875	65.7005
F8	63.0442	56.4995	351.5915	11.0843	73.8716
F9	2.96E + 03	5.92E + 03	4.17E + 03	14.2323	4.89E + 03
F10	1.50E + 03	1.39E + 03	1.26E + 03	1.78E + 03	1.15E + 03
F11	1.19E + 04	1.35E + 03	296.5727	214.0841	2.39E + 04
F12	6.11E + 08	1.93E + 08	3.04E + 06	1.84E + 05	2.59E + 10
F13	1.42E + 05	1.57E + 06	3.27E + 04	414.0912	6.53E + 09
F14	1.81E + 06	8.49E + 05	1.55E + 05	109.4971	2.50E + 07
F15	2.65E + 06	1.38E + 06	3.36E + 04	77.8945	4.28E + 09
F16	1.04E + 03	708.9840	501.8256	452.8032	2.74E + 03
F17	670.9827	640.9808	299.5947	457.5604	2.00E + 06
F18	1.68E + 06	1.54E + 06	2.40E + 05	83.5936	7.10E + 07
F19	1.46E + 07	2.64E + 06	3.10E + 05	292.9596	2.67E + 09
F20	494.2066	386.0267	418.0431	317.3453	538.5749
F21	130.6596	151.2313	572.1454	12.5380	196.4940
F22	1.50E + 03	1.64E + 03	1.34E + 03	1.69E + 03	923.8971
F23	249.9539	209.1528	439.4192	18.7462	370.5127
F24	400.9515	336.1390	16.5380	24.8663	870.5824
F25	149.5246	105.6238	45.6092	49.5623	2.88E + 03
F26	2.78E + 03	1.99E + 03	735.6929	1.28E + 03	3.56E + 03
F27	530.9462	455.9865	27.4029	22.3489	958.3921
F28	336.0489	100.0358	29.9038	29.7901	2.31E + 03
F29	1.13E + 03	694.3518	236.9015	409.1394	1.53E + 05
F30	1.95E + 08	1.96E + 07	1.59E + 06	1.91E + 03	7.14E + 09

## Analysis of SSO performance in solving the CEC-BC-2017 test suite

For a more accurate analysis of the performance of the SSO algorithm presented in this paper, one of the most challenging numerical optimization functions are chosen to evaluate the general performance of the SSO. CEC-BC-2017 competition with 30 functions containing at least 15 hybrid and composite functions, are chosen here for this validation. SSO algorithm is tested over CEC-BC-2017 functions with all available dimensions and the results are compared with that of HHO, CMA-ES, EBOwithCMAR and AOA algorithms. All of the available dimensions (10,30,50,100) are considered for all of the CEC-BC-2017 test functions and the experiment is run 30 times with 150 search agents and a maximum number of iterations of 1000 times. The parameters configured for solving these test functions with are as the same as provided in the Table 1.

Figures 25, 26, 27, 28, 29, 30, 31, 32, 33, 34, 35, 36, 37, 38, 39, 40, 41, 42, 43, 44, 45, 46, 47, 48, 49, 50, 51, 52, 53, 54, 55, 56, 57, 58, 59, 60, 61, 62, 63, 64, 65, 66, 67, 68, 69, 70, 71, 72, 73, 74, 75, 76, 77, 78, 79, 80, 81, 82, 83, 84, 85, 86, 87, 88, 89, 90, 91, 92, 93, 94, 95, 96, 97, 98, 99, 100, 101, 102, 103, 104, 105, 106, 107, 108, 109, 110, 111, 112, 113, 114, 115, 116, 117, 118, 119, 120, 121, 122, 123, 124, 125, 126, 127, 128, 129, 130, 131, 132, 133, 134, 135, 136, 137, 138, 139, 140 show the convergence curves of CEC-BC-2017 test functions of all algorithms.

**Figure 25:**
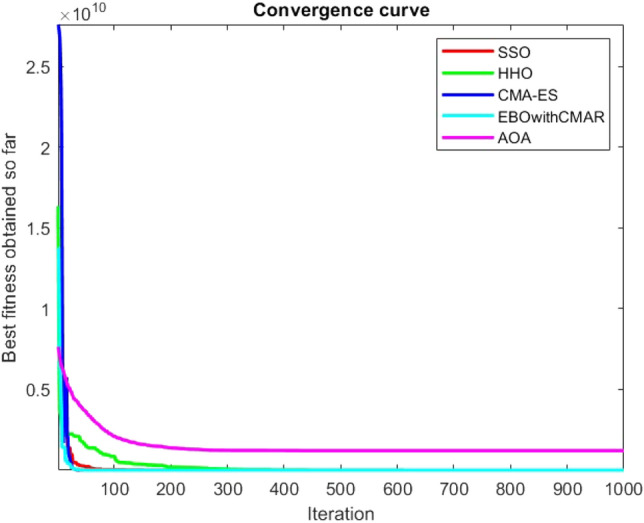
ComplexTest function 1 (Dimension = 10).

**Figure 26:**
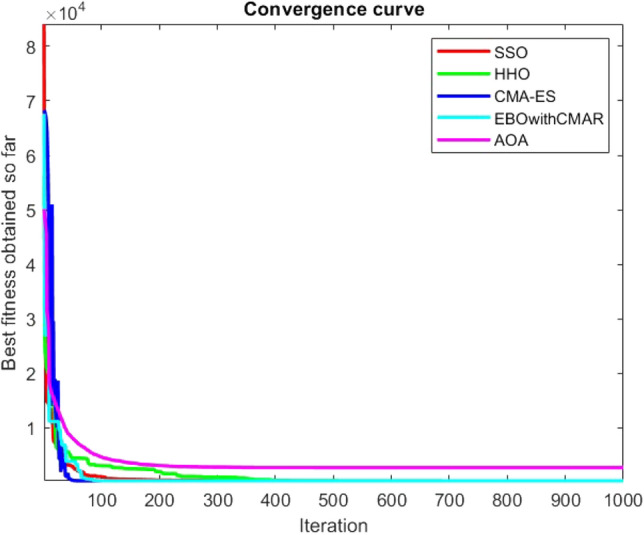
ComplexTest function 3 (Dimension = 10).

**Figure 27:**
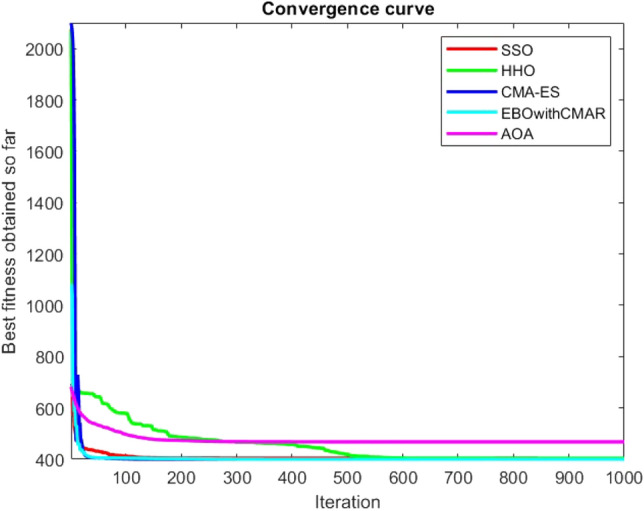
ComplexTest function 4 (Dimension = 10).

**Figure 28:**
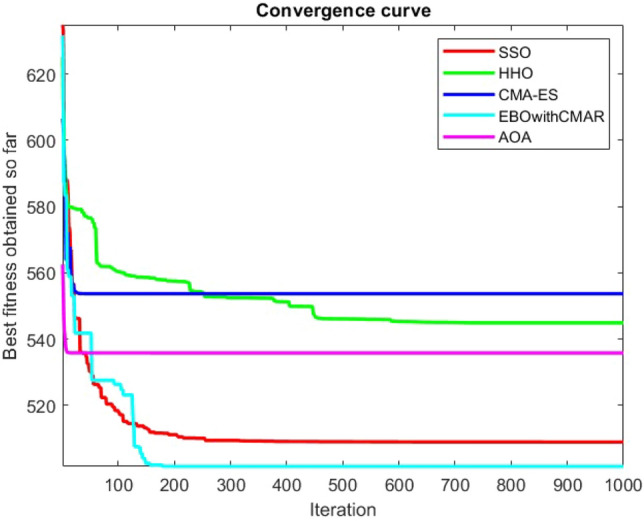
ComplexTest function 5 (Dimension = 10).

**Figure 29:**
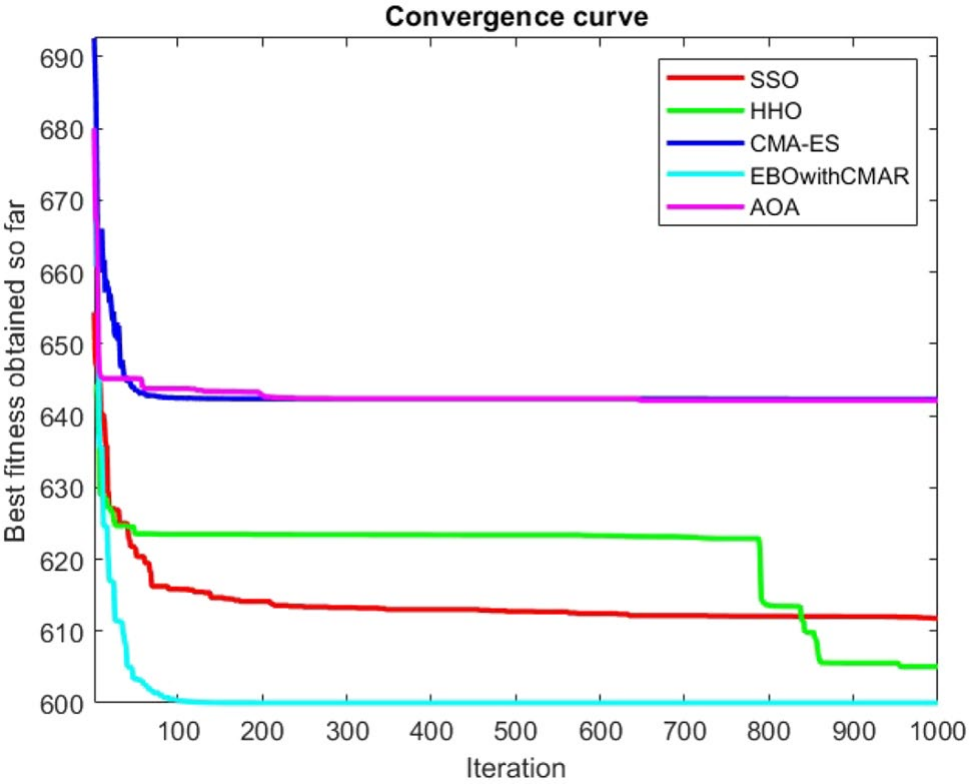
ComplexTest function 6 (Dimension = 10).

**Figure 30:**
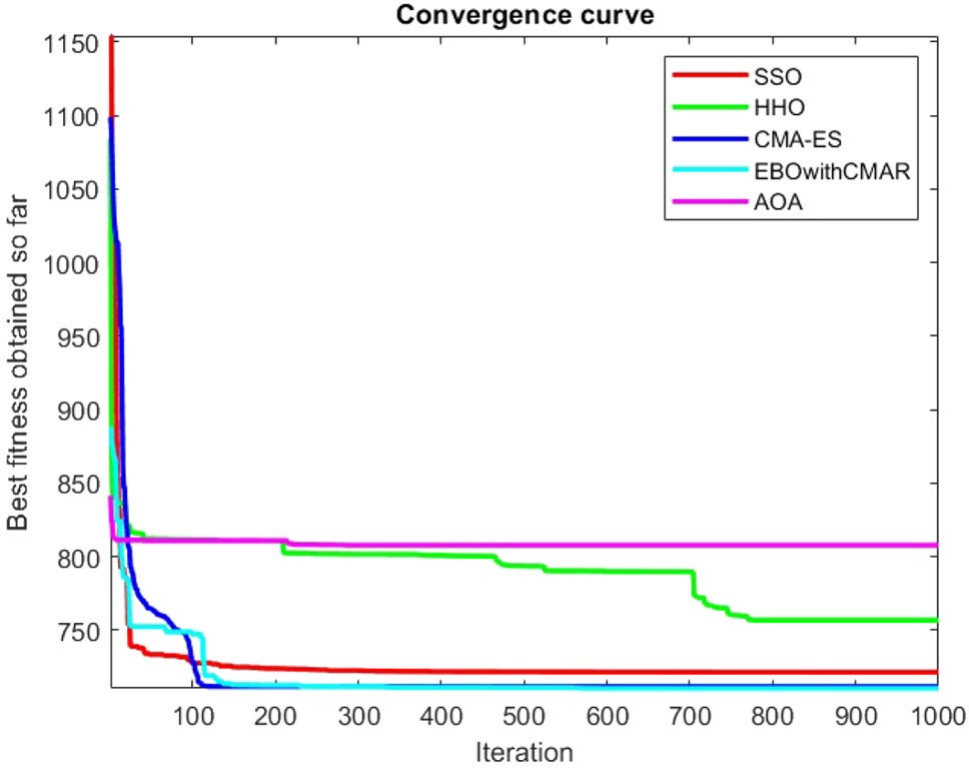
ComplexTest function 7 (Dimension = 10).

**Figure 31:**
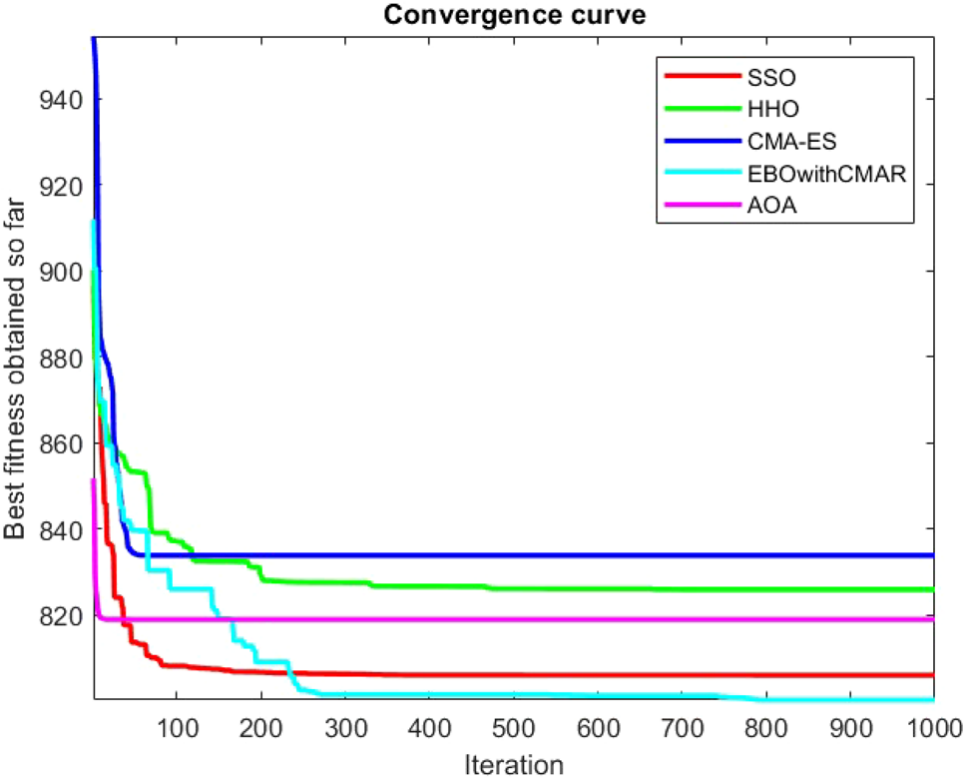
ComplexTest function 8 (Dimension = 10).

**Figure 32:**
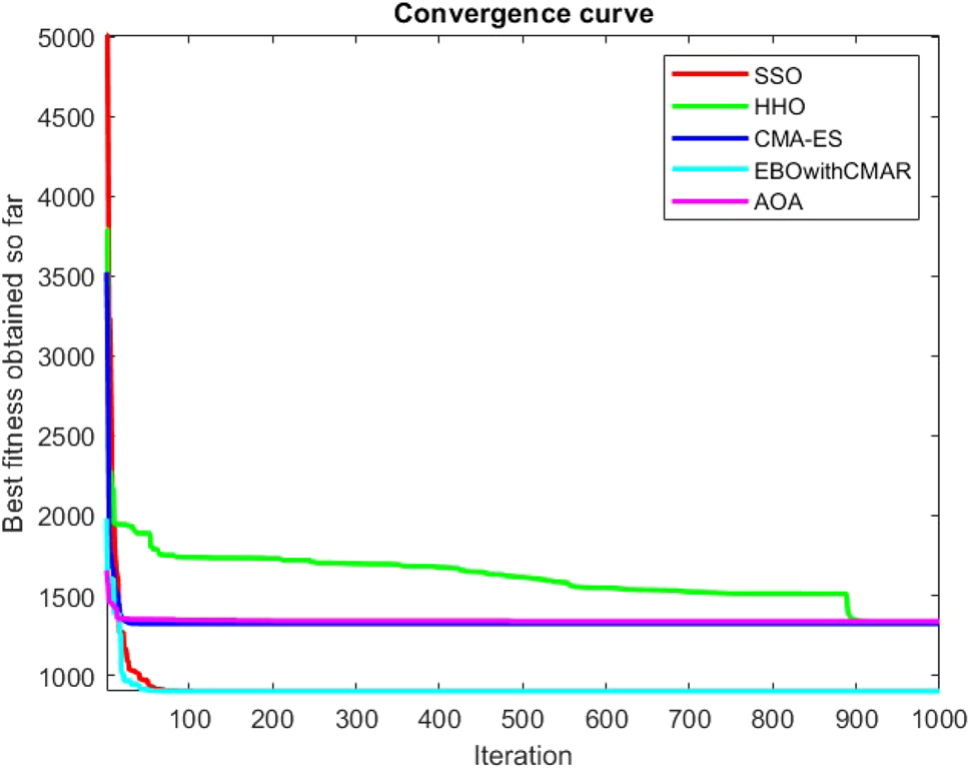
ComplexTest function 9 (Dimension = 10).

**Figure 33:**
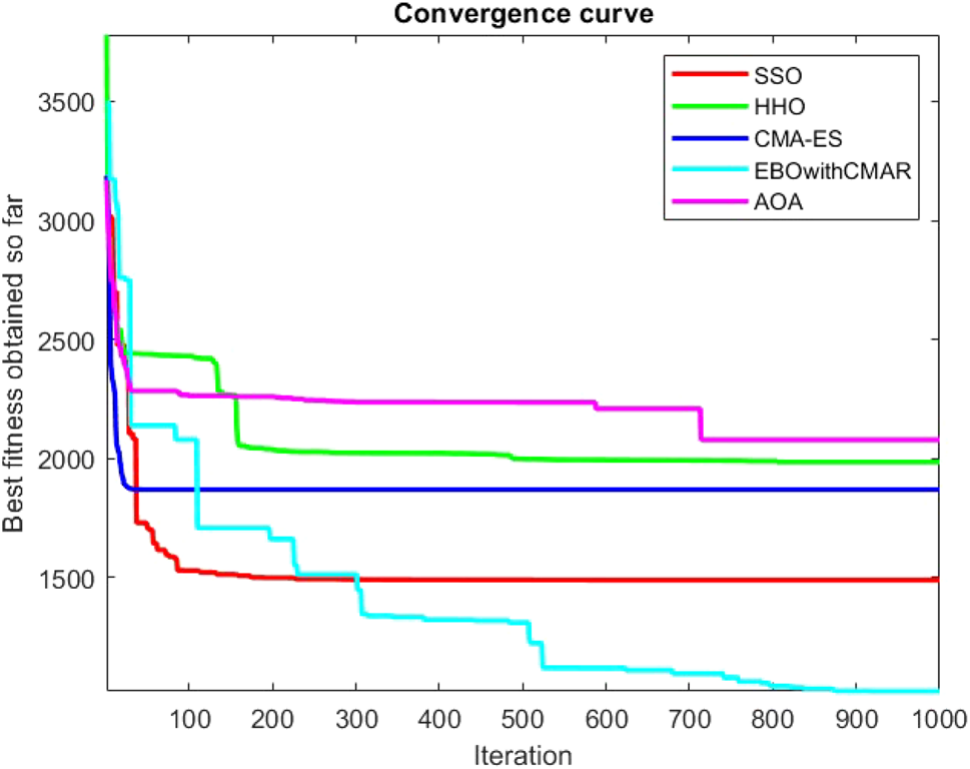
ComplexTest function 10 (Dimension = 10).

**Figure 34:**
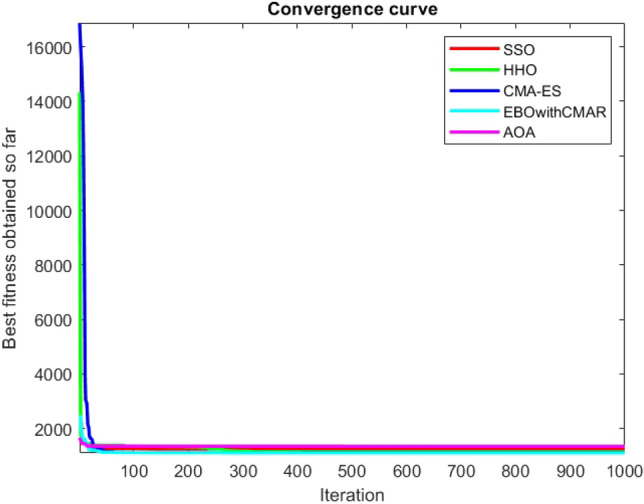
ComplexTest function 11 (Dimension = 10).

**Figure 35:**
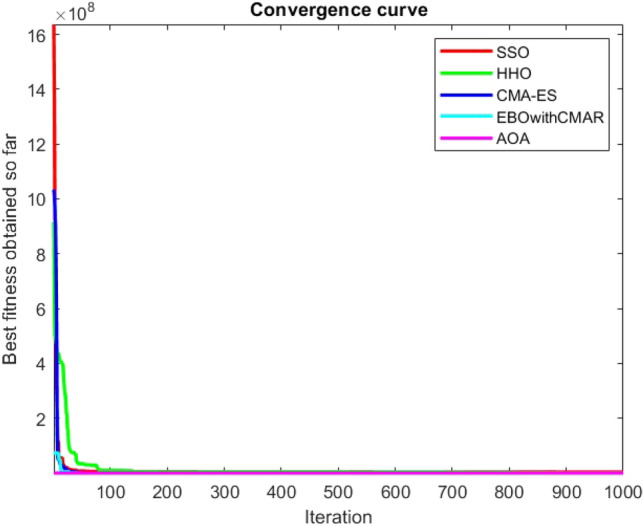
ComplexTest function 12 (Dimension = 10).

**Figure 36:**
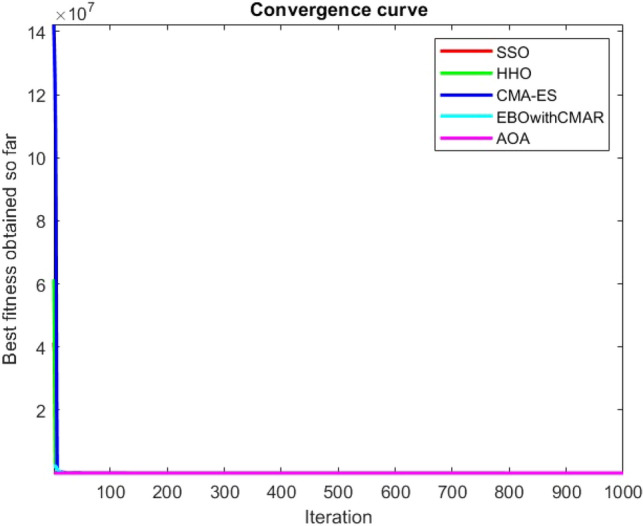
ComplexTest function 13 (Dimension = 10).

**Figure 37:**
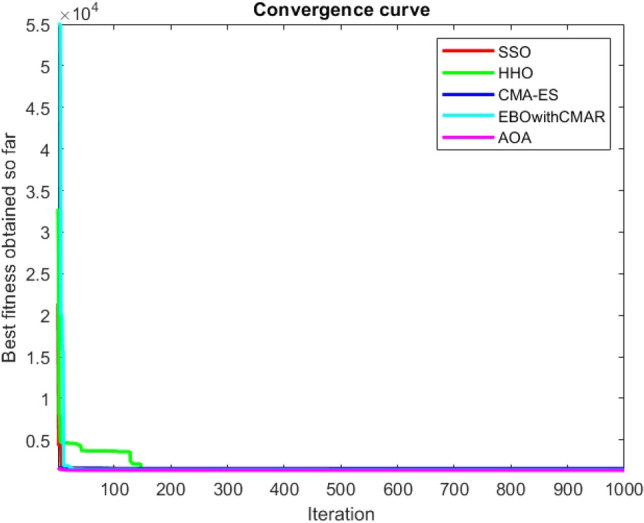
ComplexTest function 14 (Dimension = 10).

**Figure 38:**
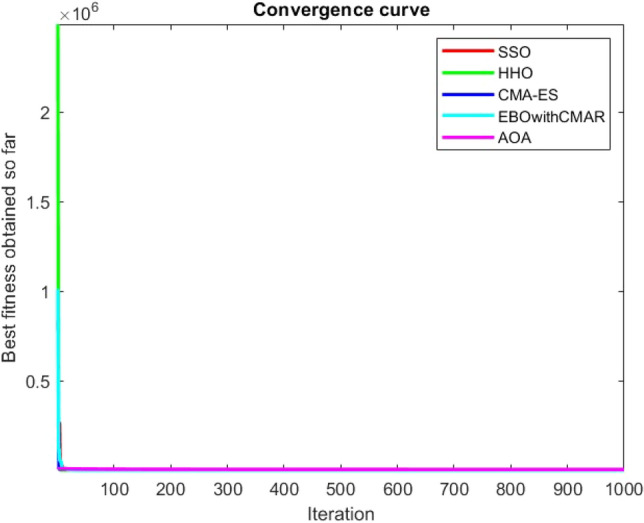
ComplexTest function 15 (Dimension = 10).

**Figure 39:**
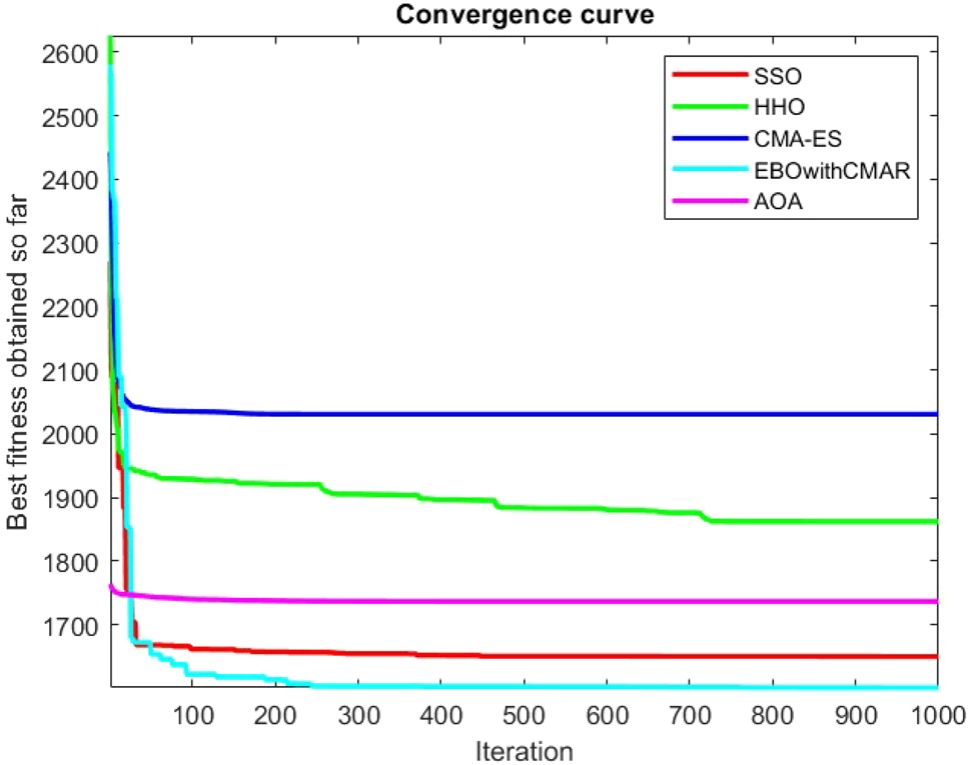
ComplexTest function 16 (Dimension = 10).

**Figure 40:**
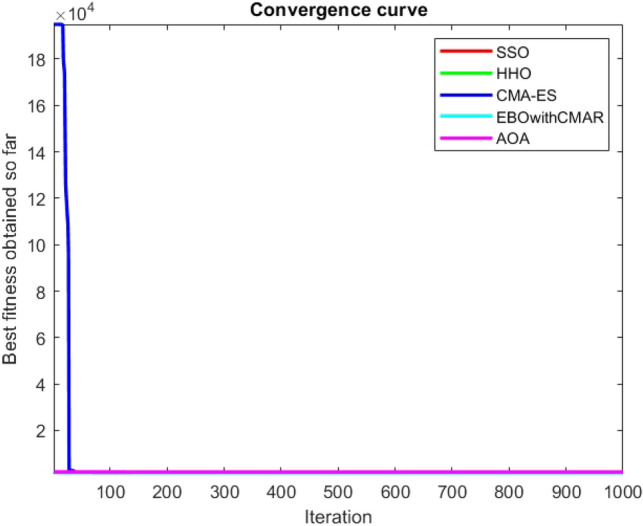
ComplexTest function 17 (Dimension = 10).

**Figure 41:**
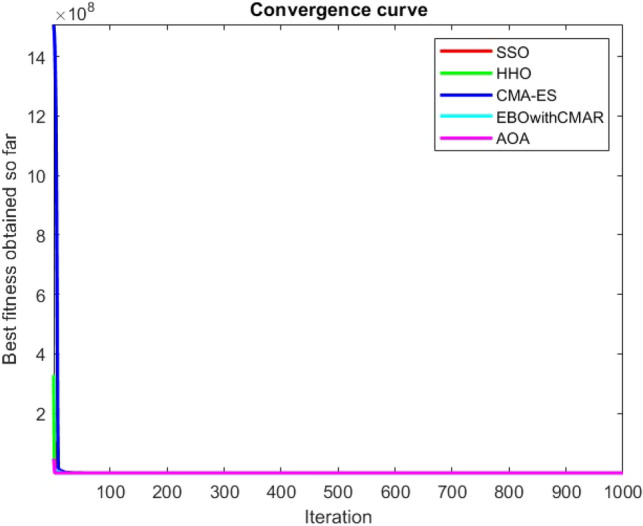
ComplexTest function 18 (Dimension = 10).

**Figure 42:**
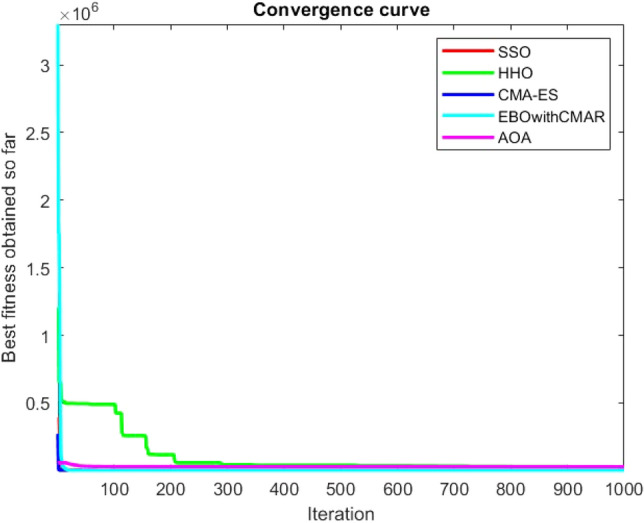
ComplexTest function 19 (Dimension = 10).

**Figure 43:**
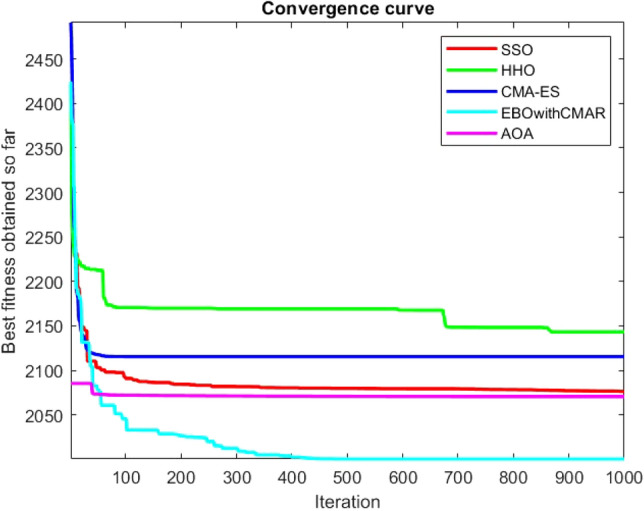
ComplexTest function 20 (Dimension = 10).

**Figure 44:**
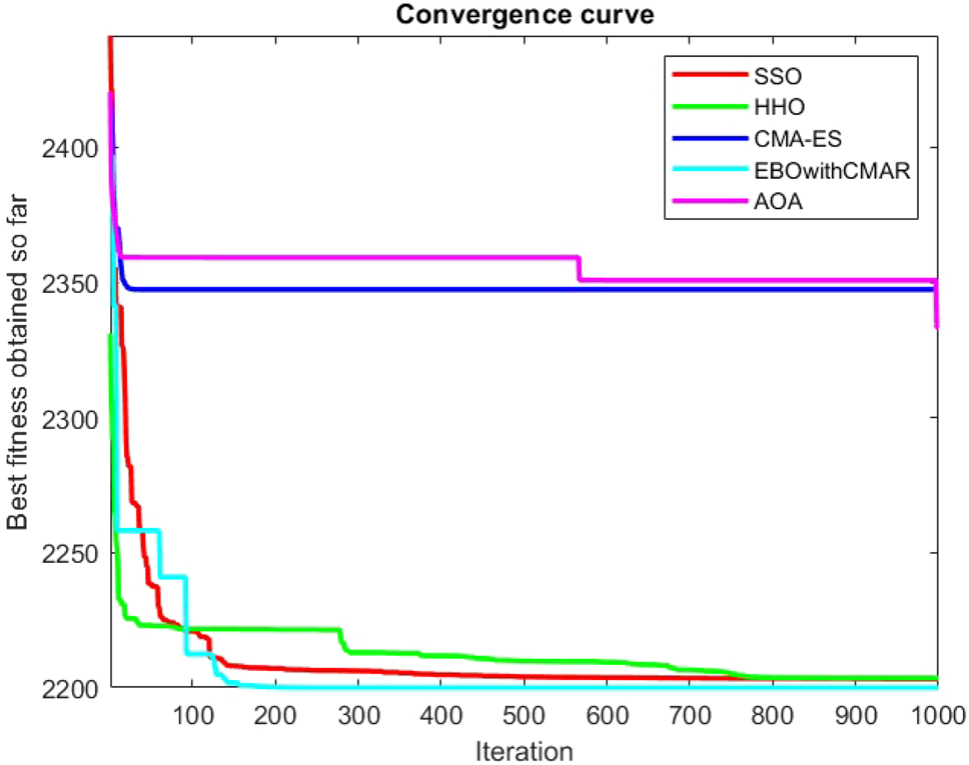
ComplexTest function 21 (Dimension = 10).

**Figure 45:**
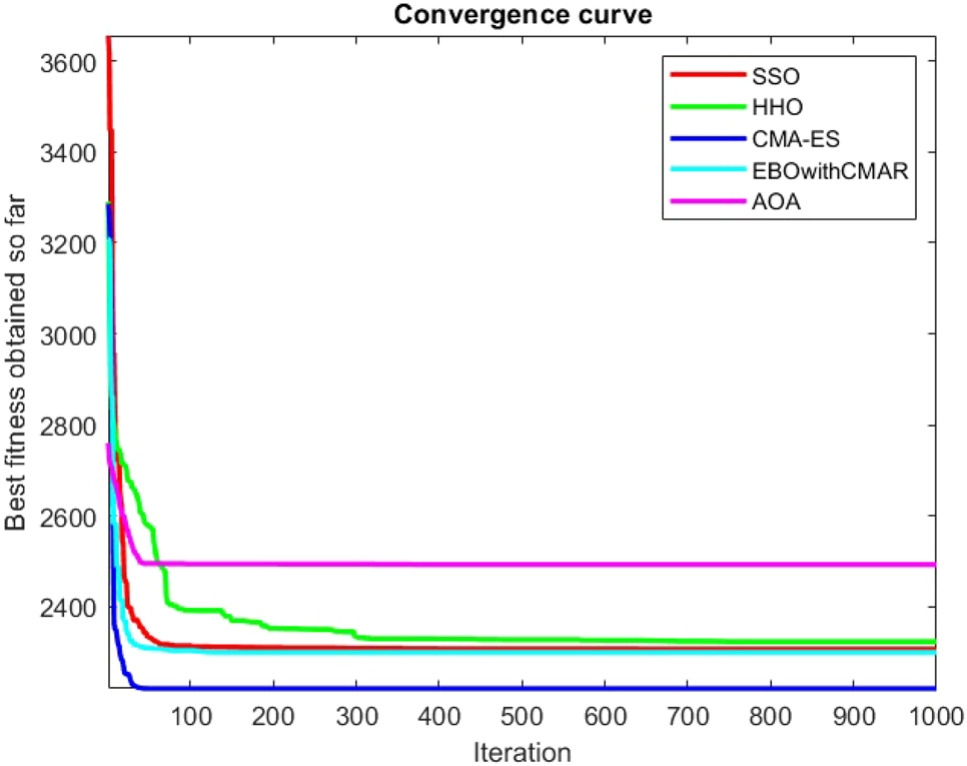
ComplexTest function 22 (Dimension = 10).

**Figure 46:**
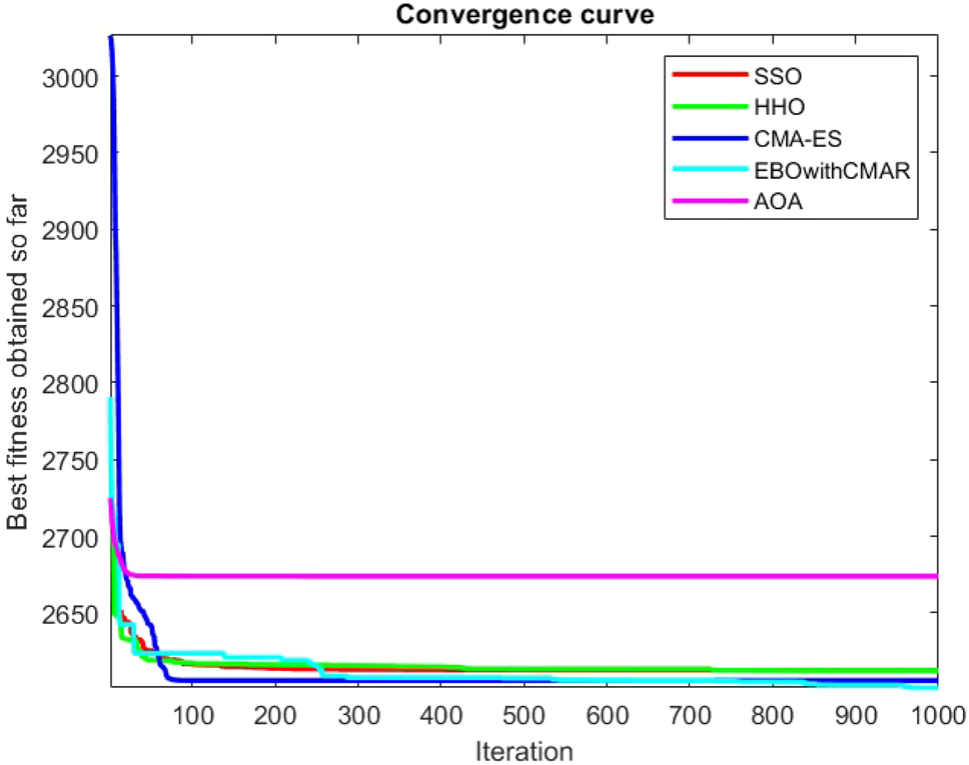
ComplexTest function 23 (Dimension = 10).

**Figure 47:**
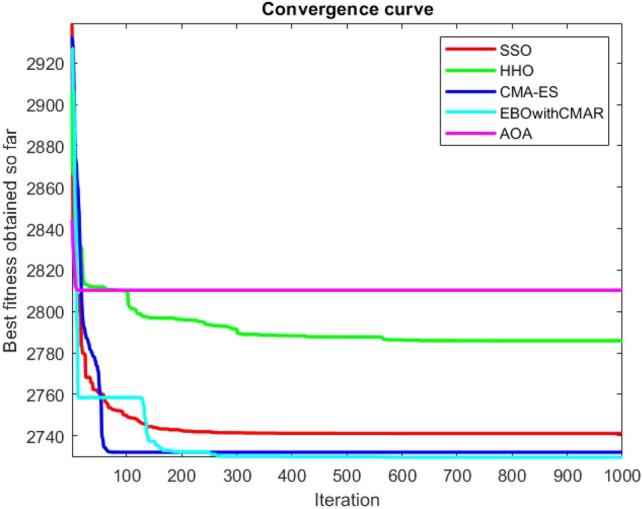
ComplexTest function 24 (Dimension = 10).

**Figure 48:**
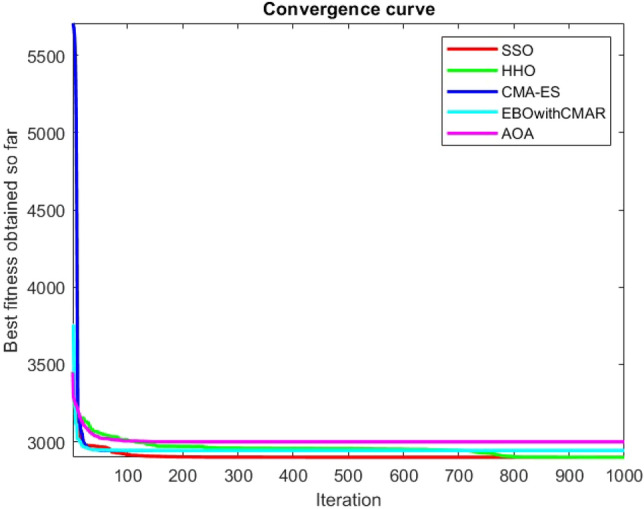
ComplexTest function 25 (Dimension = 10).

**Figure 49:**
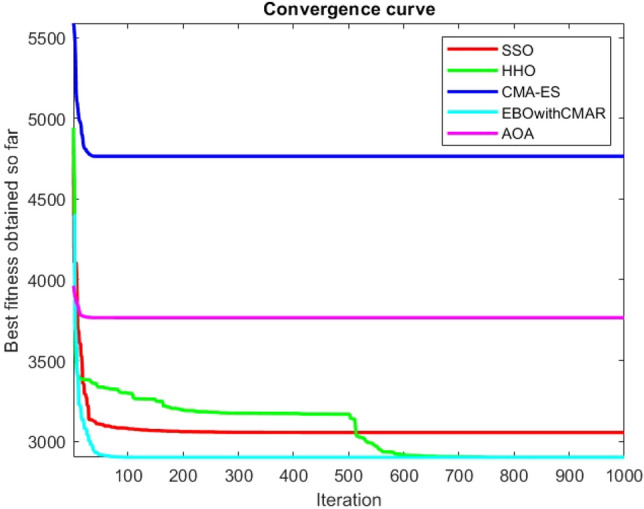
ComplexTest function 26 (Dimension = 10).

**Figure 50:**
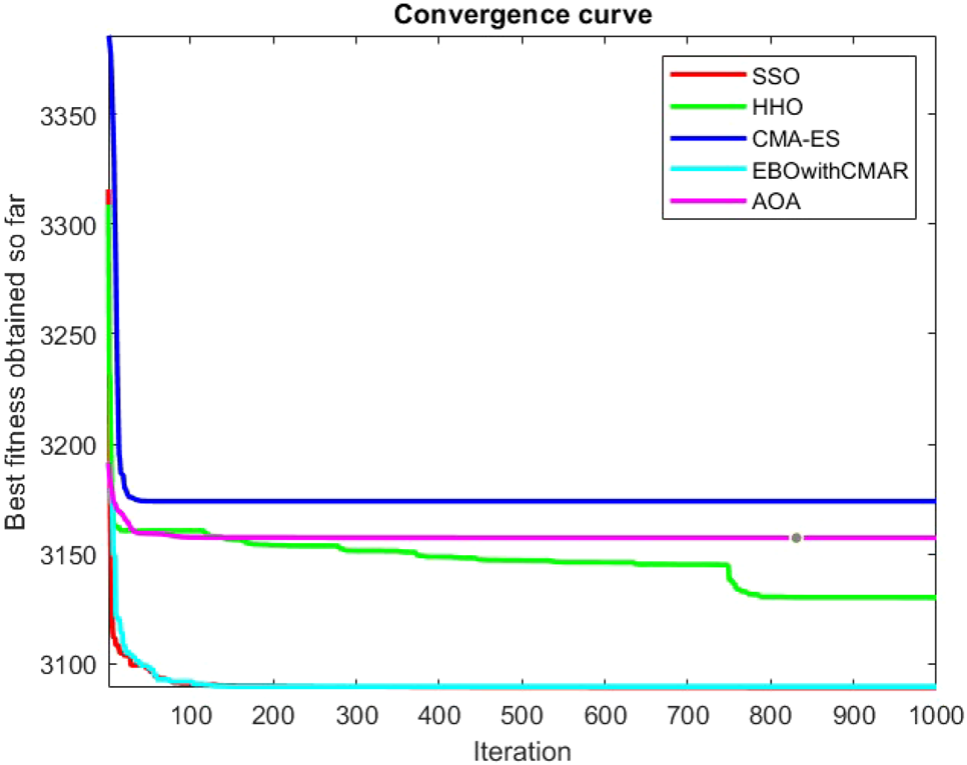
ComplexTest function 27 (Dimension = 10).

**Figure 51:**
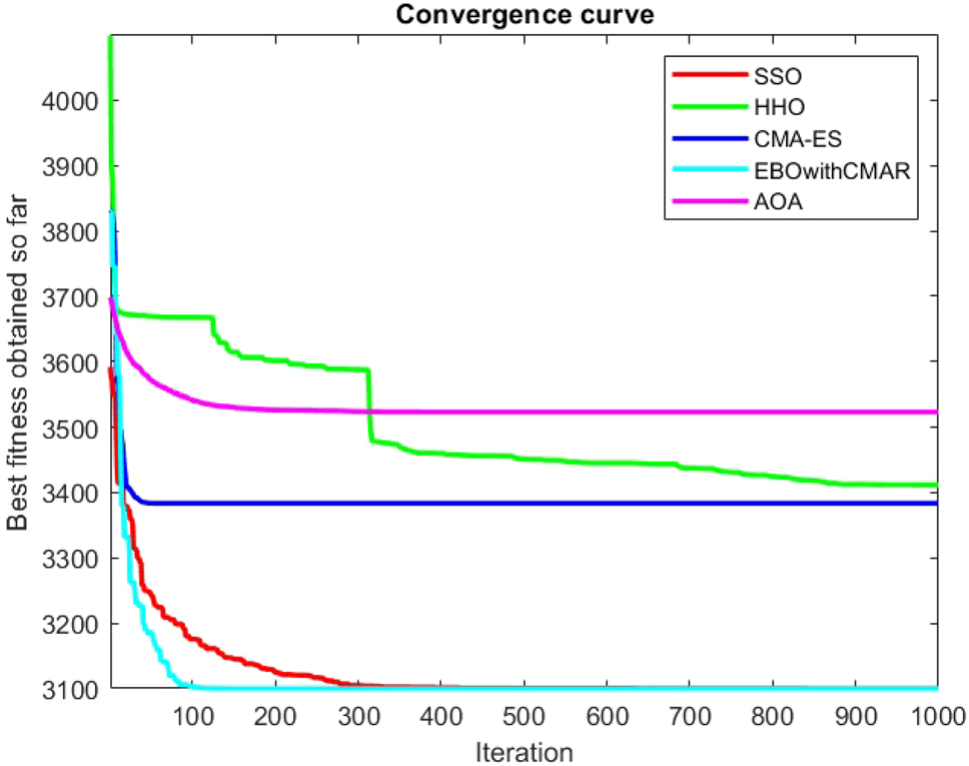
ComplexTest function 28 (Dimension = 10).

**Figure 52:**
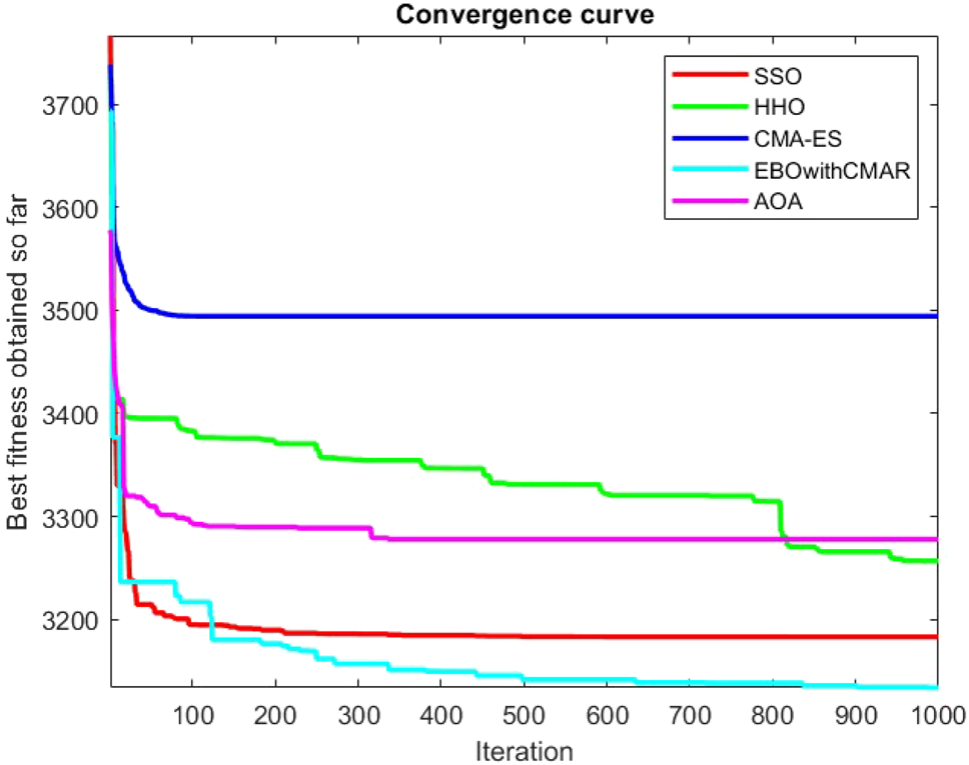
ComplexTest function 29 (Dimension = 10).

**Figure 53:**
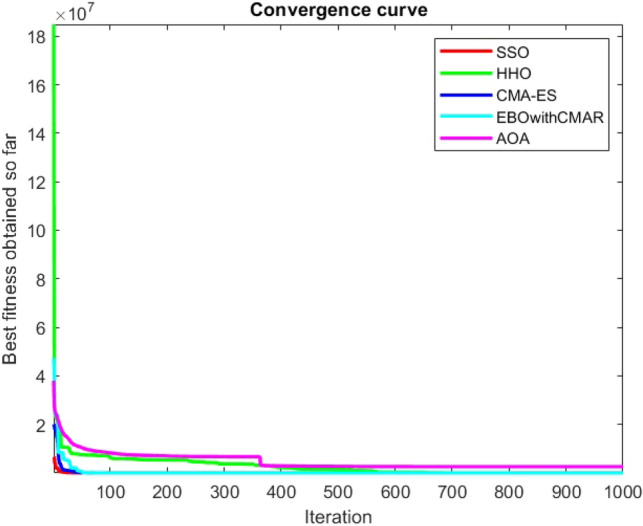
ComplexTest function 30 (Dimension = 10).

**Figure 54:**
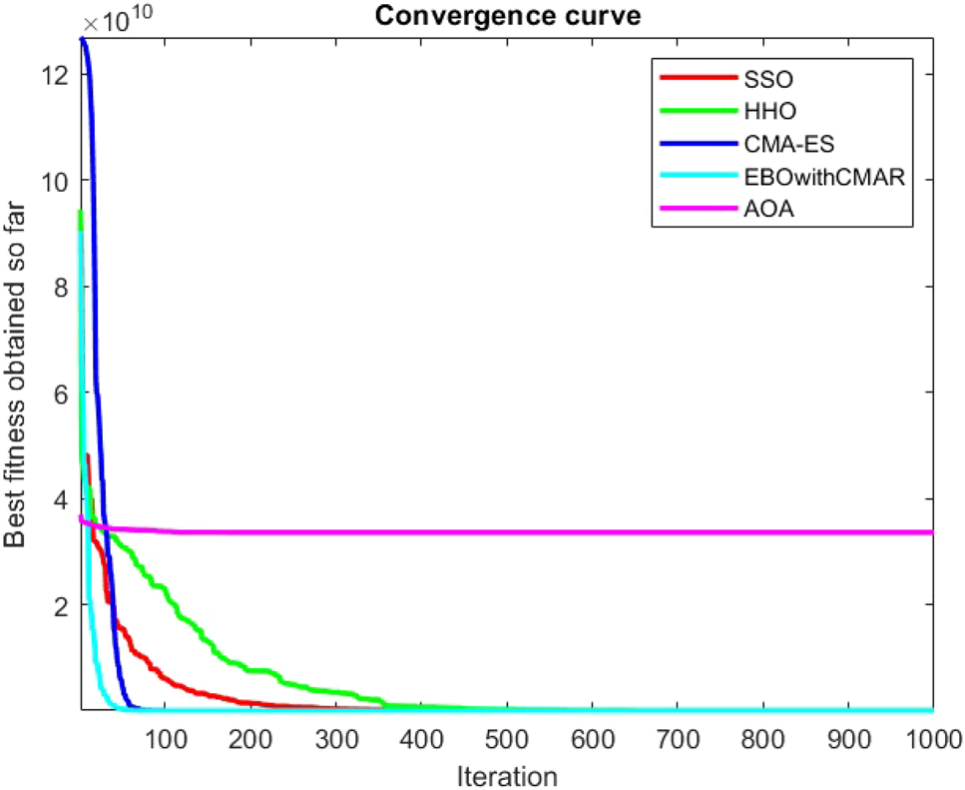
ComplexTest function 1 (Dimension = 30).

**Figure 55:**
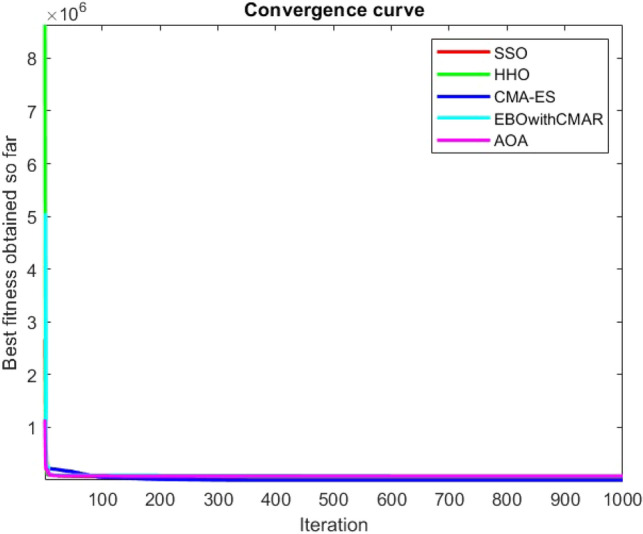
ComplexTest function 3 (Dimension = 30).

**Figure 56:**
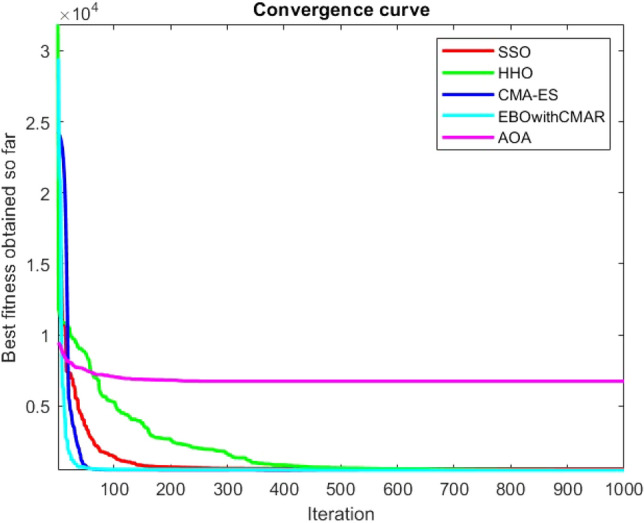
ComplexTest function 4 (Dimension = 30).

**Figure 57:**
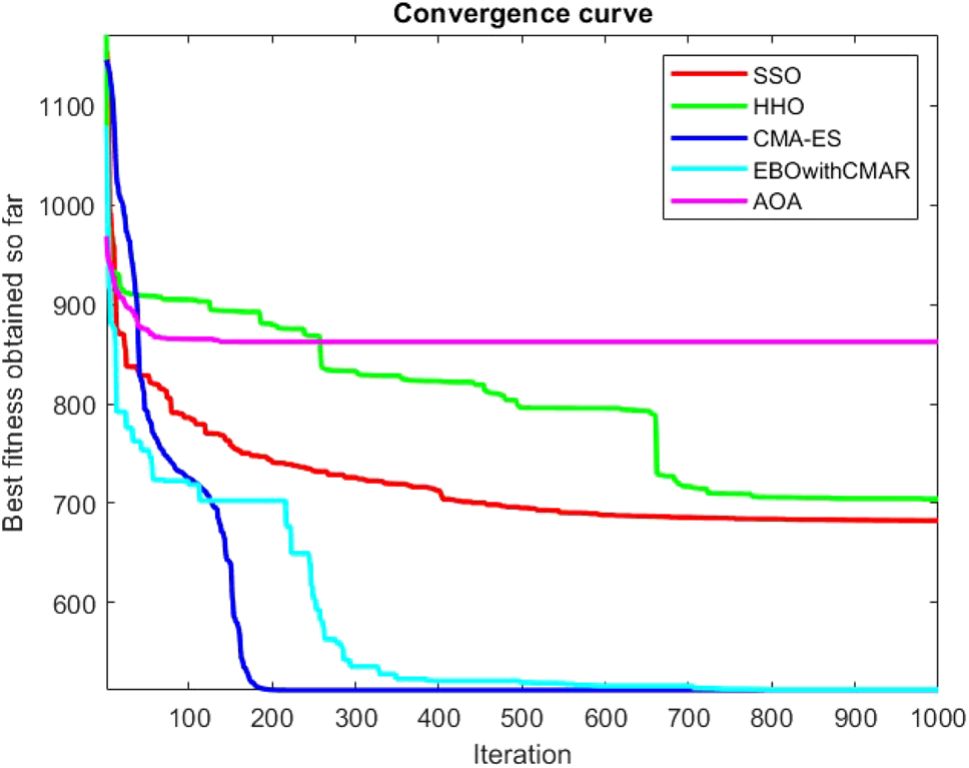
ComplexTest function 5 (Dimension = 30).

**Figure 58:**
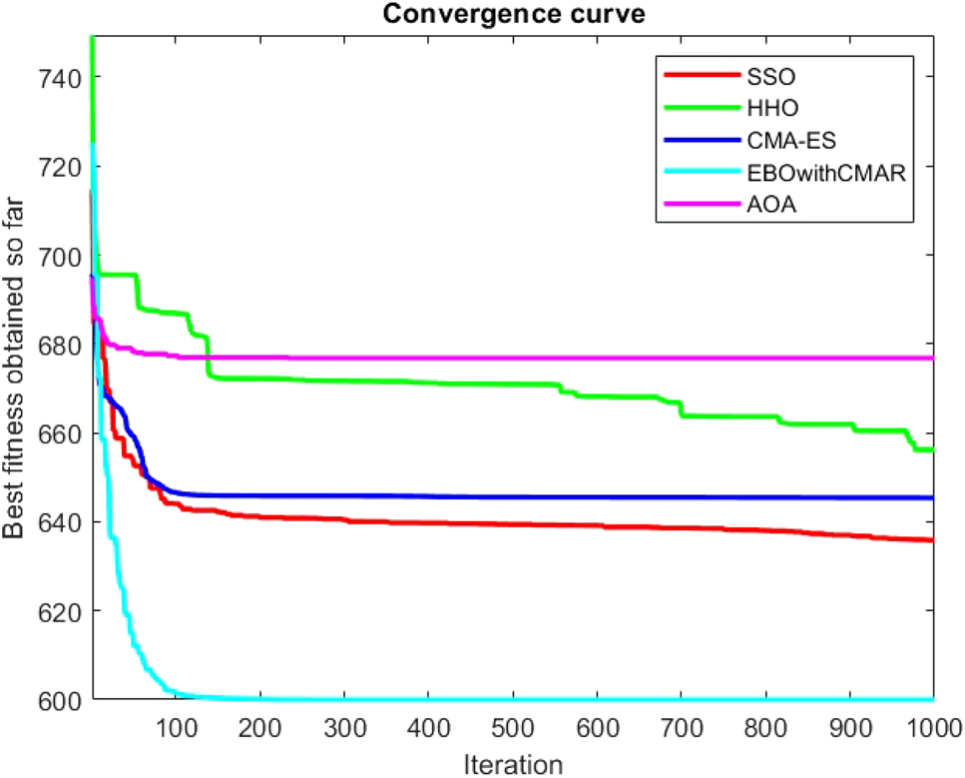
ComplexTest function 6 (Dimension = 30).

**Figure 59:**
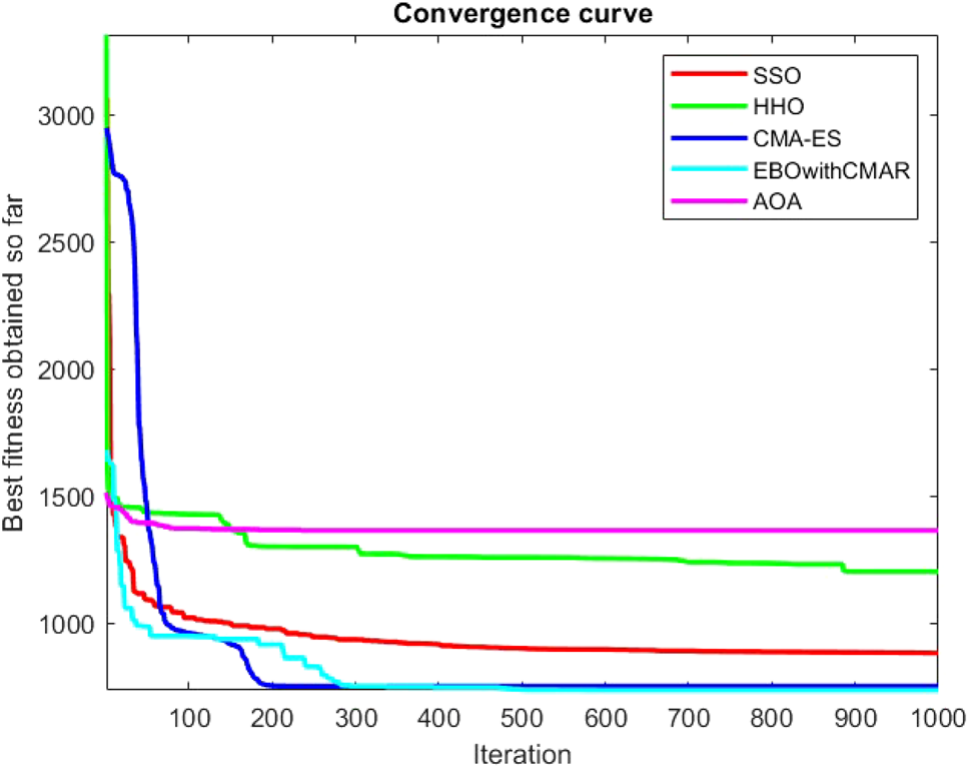
ComplexTest function 7 (Dimension = 30).

**Figure 60:**
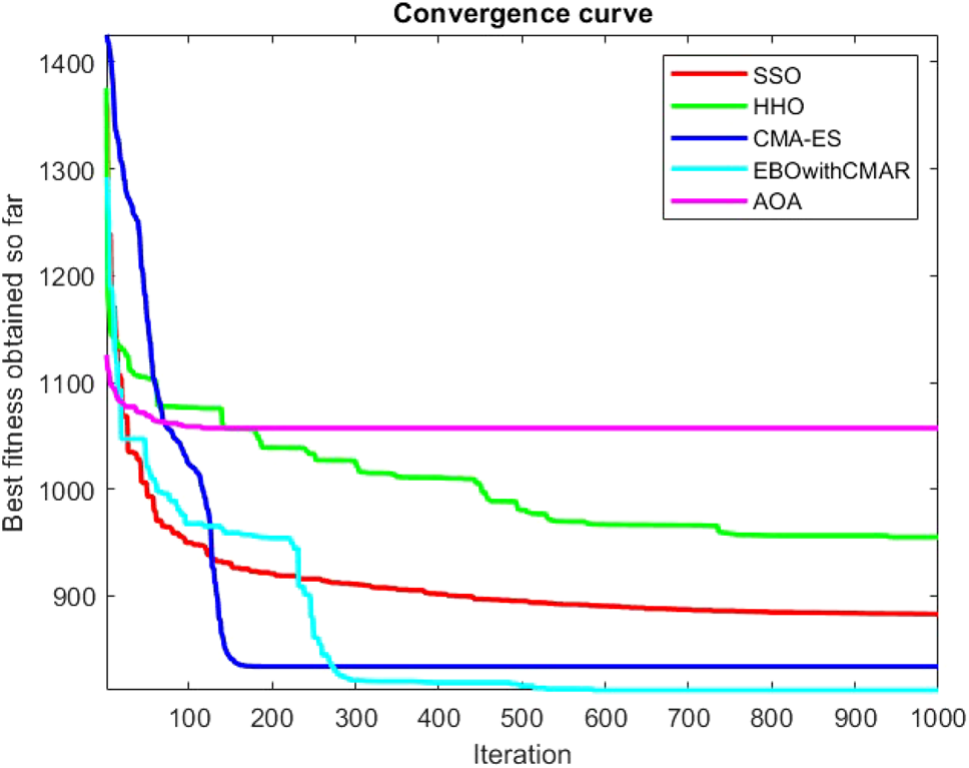
ComplexTest function 8 (Dimension = 30).

**Figure 61:**
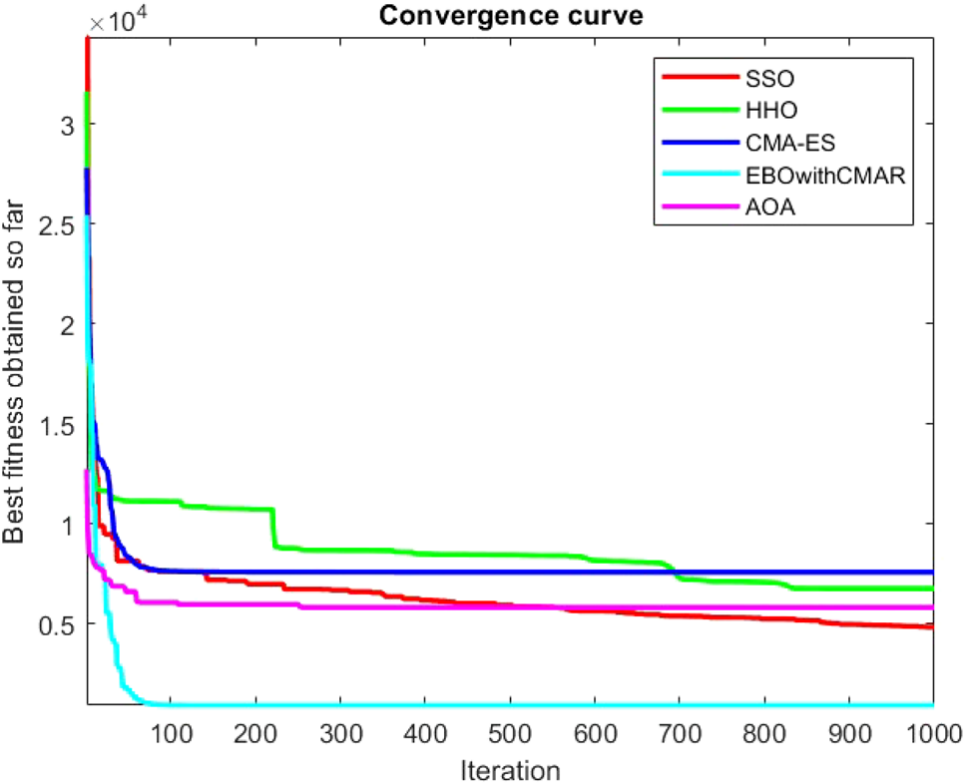
ComplexTest function 9 (Dimension = 30).

**Figure 62:**
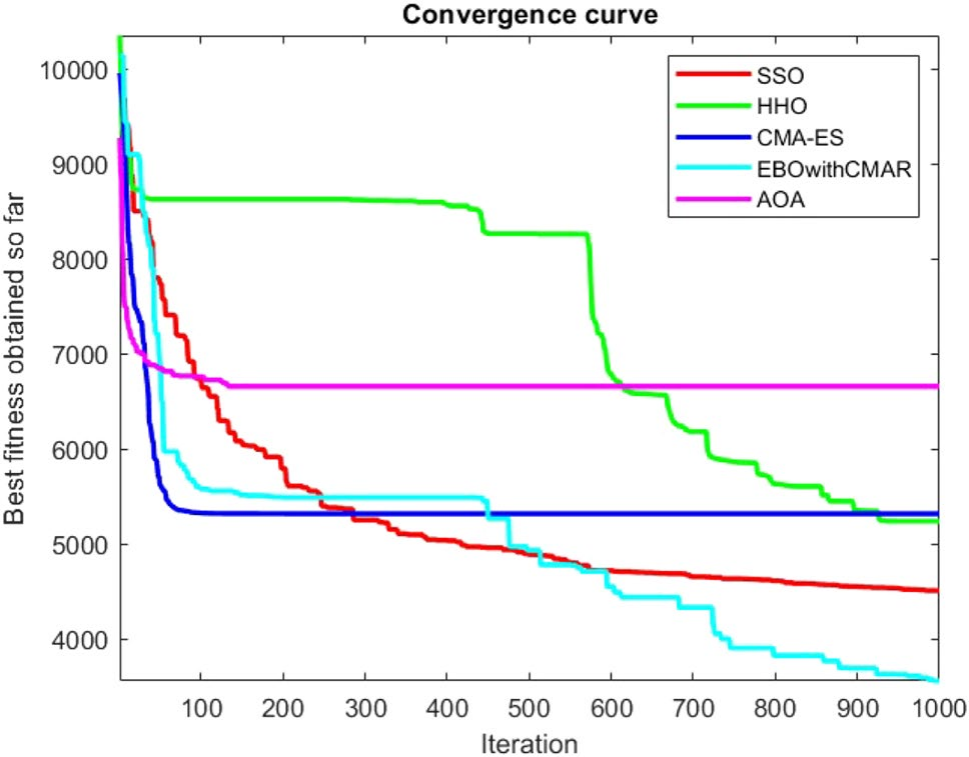
ComplexTest function 10 (Dimension = 30).

**Figure 63:**
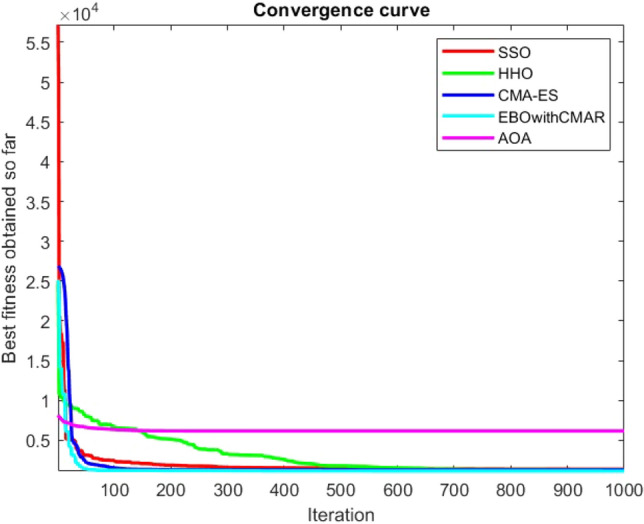
ComplexTest function 11 (Dimension = 30).

**Figure 64:**
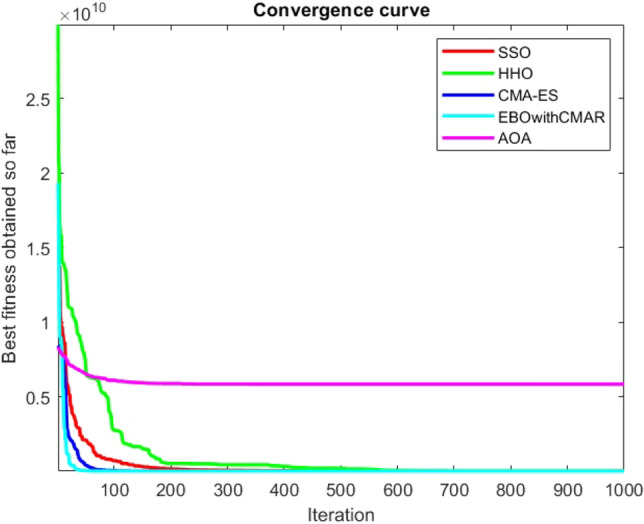
ComplexTest function 12 (Dimension = 30).

**Figure 65:**
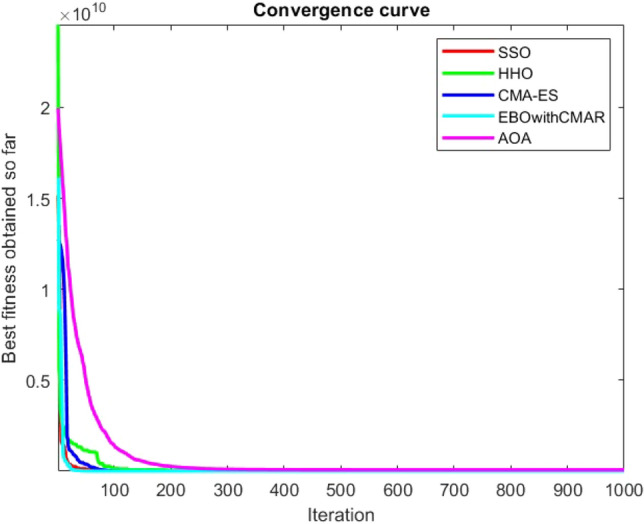
ComplexTest function 13 (Dimension = 30).

**Figure 66:**
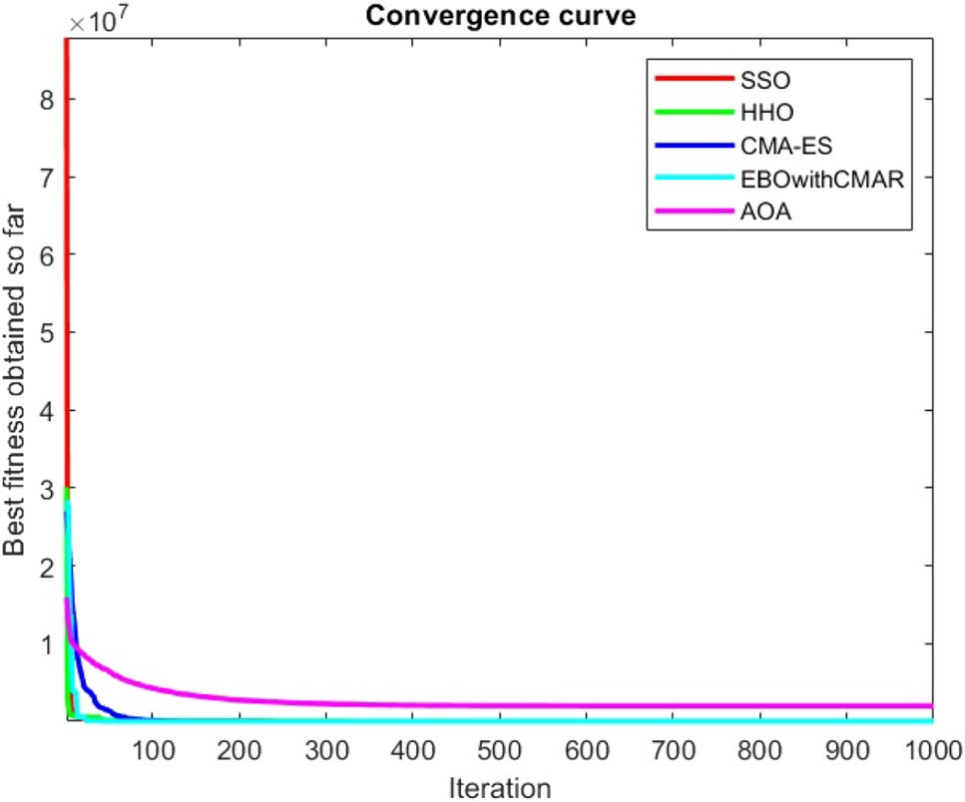
ComplexTest function 14 (Dimension = 30).

**Figure 67:**
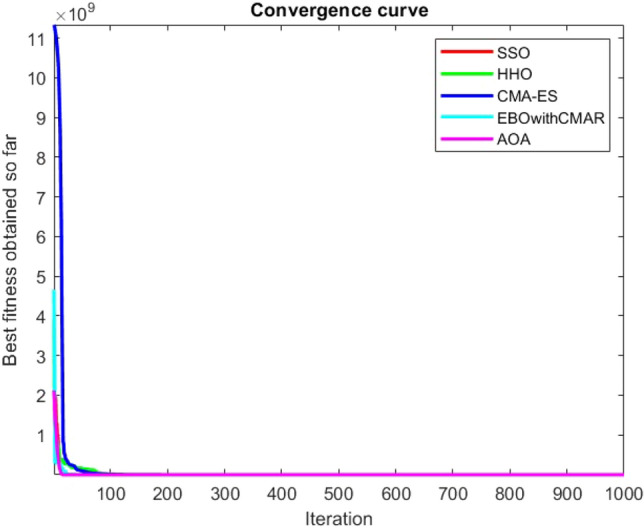
ComplexTest function 15 (Dimension = 30).

**Figure 68:**
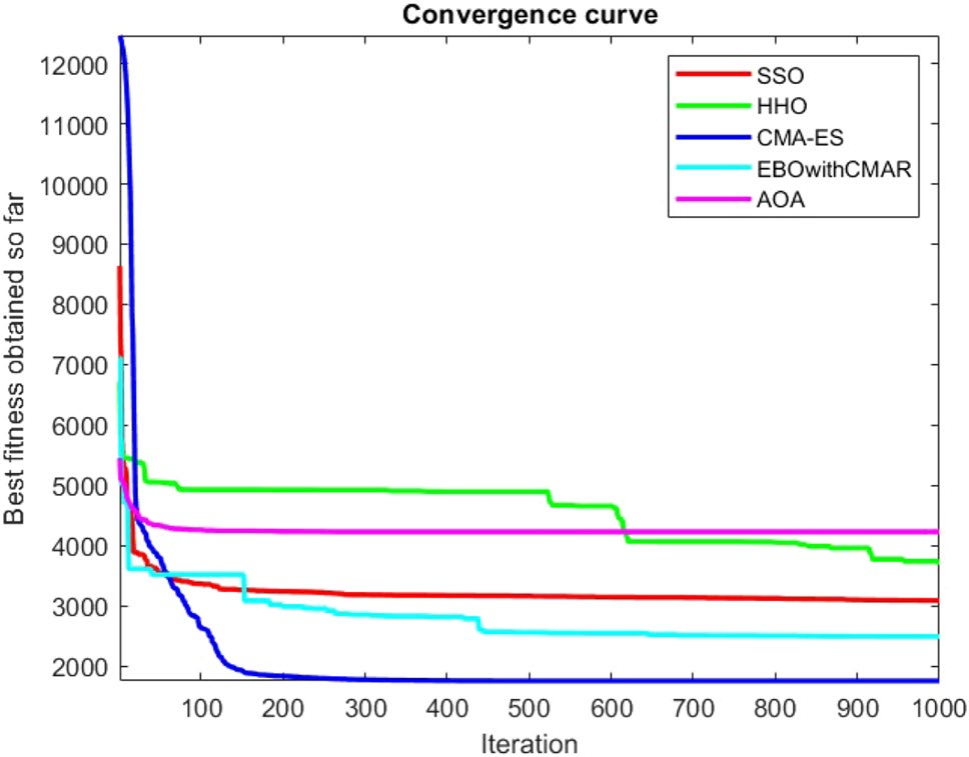
ComplexTest function 16 (Dimension = 30).

**Figure 69:**
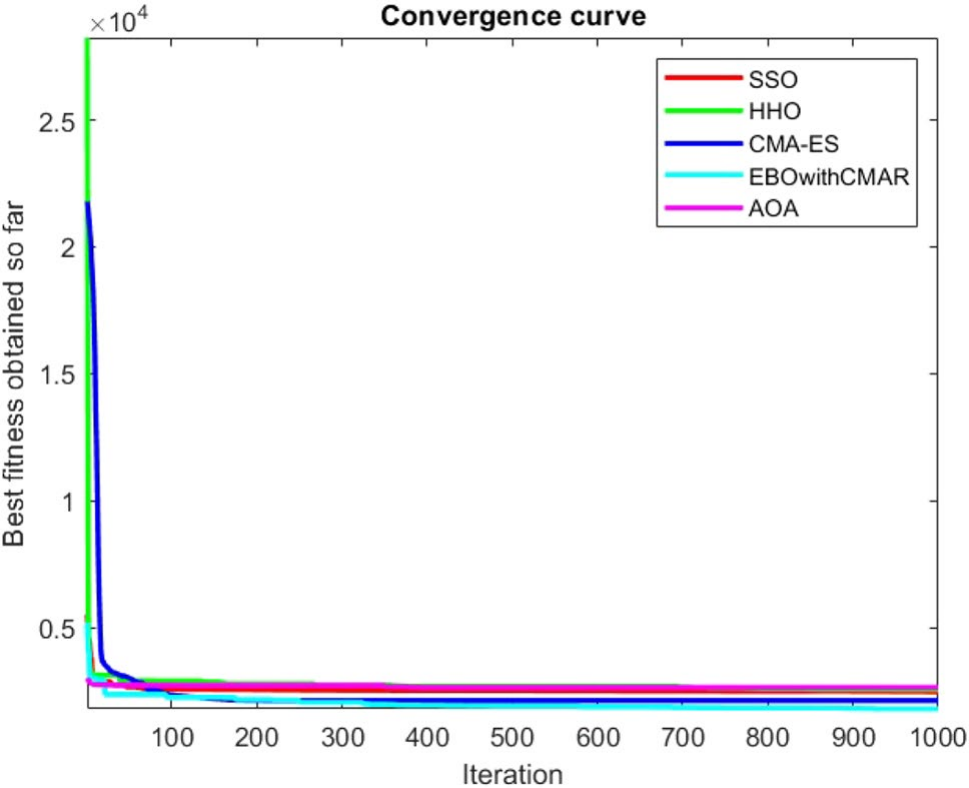
ComplexTest function 17 (Dimension = 30).

**Figure 70:**
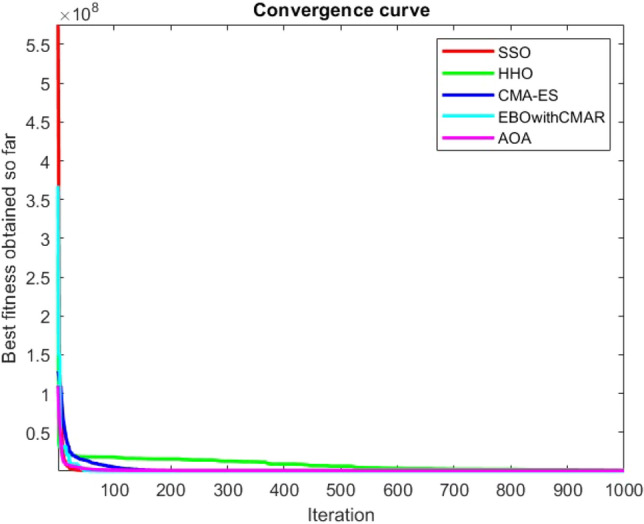
ComplexTest function 18 (Dimension = 30).

**Figure 71:**
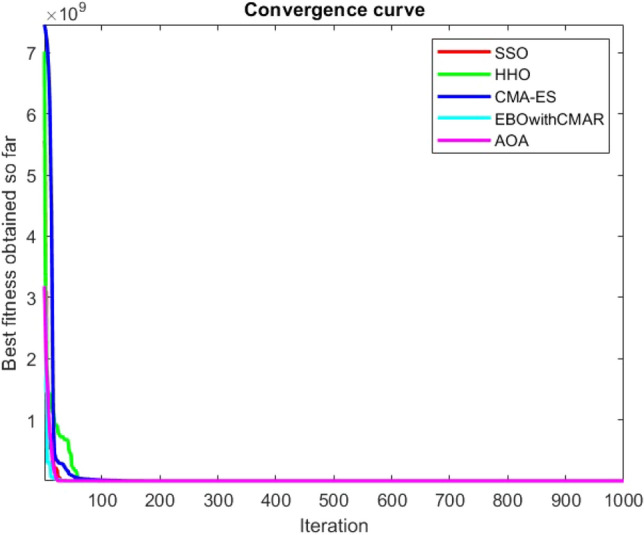
ComplexTest function 19 (Dimension = 30).

**Figure 72:**
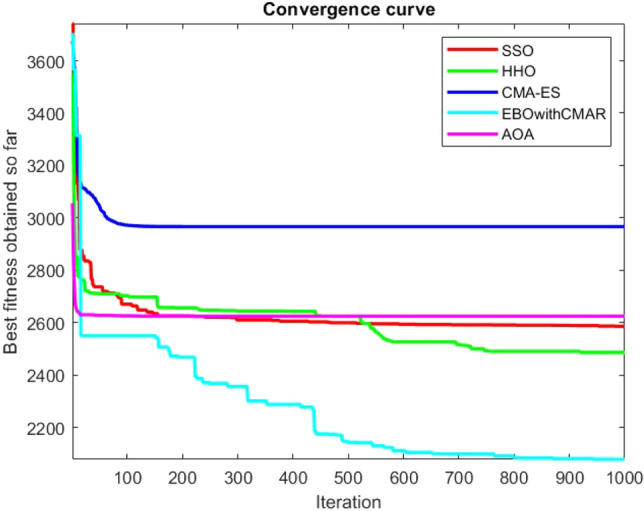
ComplexTest function 20 (Dimension = 30).

**Figure 73:**
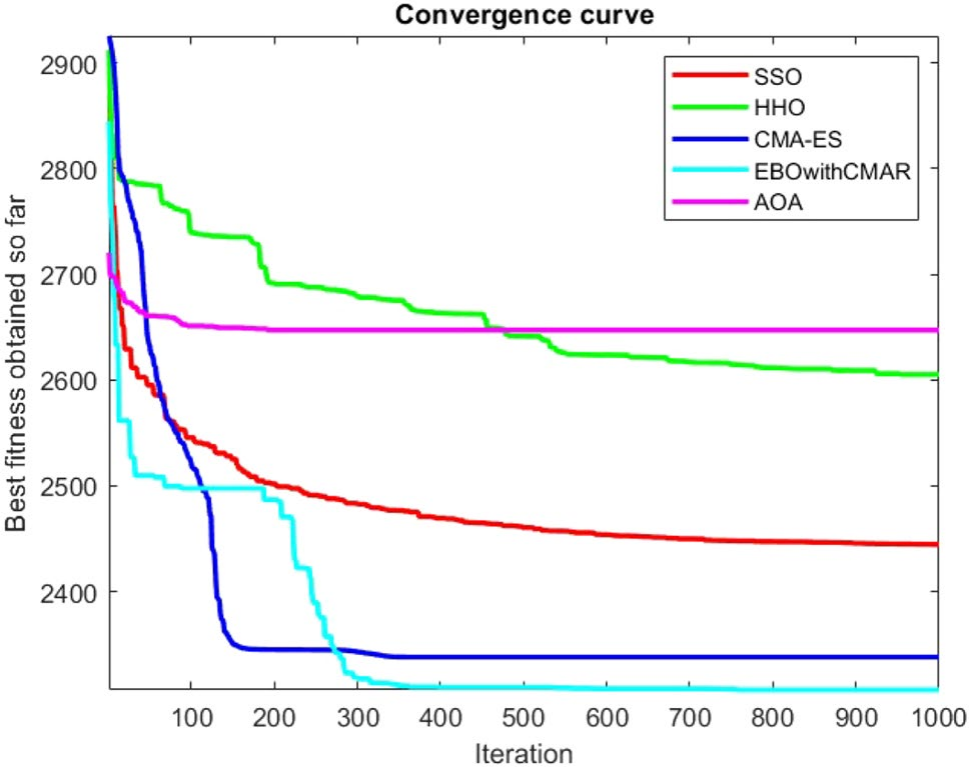
ComplexTest function 21 (Dimension = 30).

**Figure 74:**
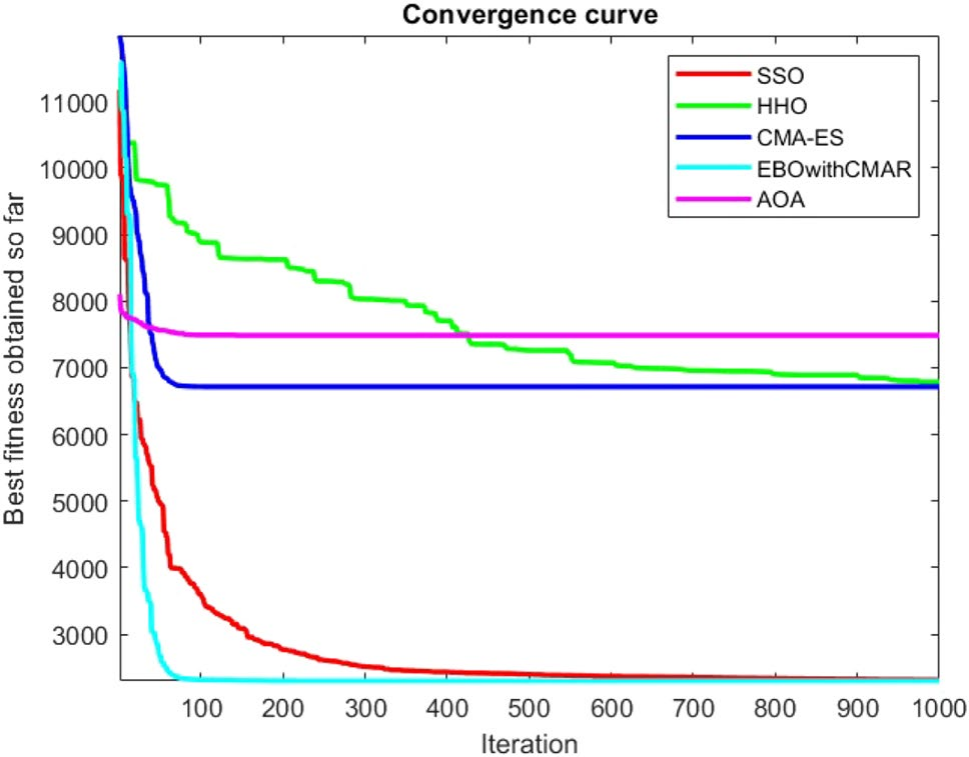
ComplexTest function 22 (Dimension = 30).

**Figure 75:**
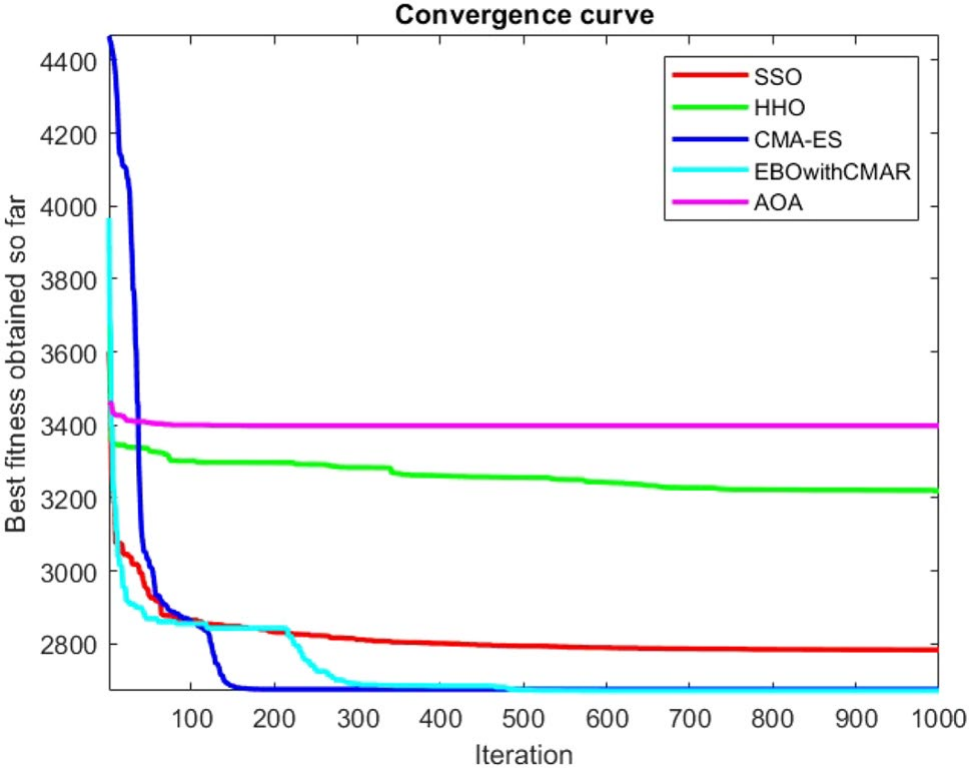
ComplexTest function 23 (Dimension = 30).

**Figure 76:**
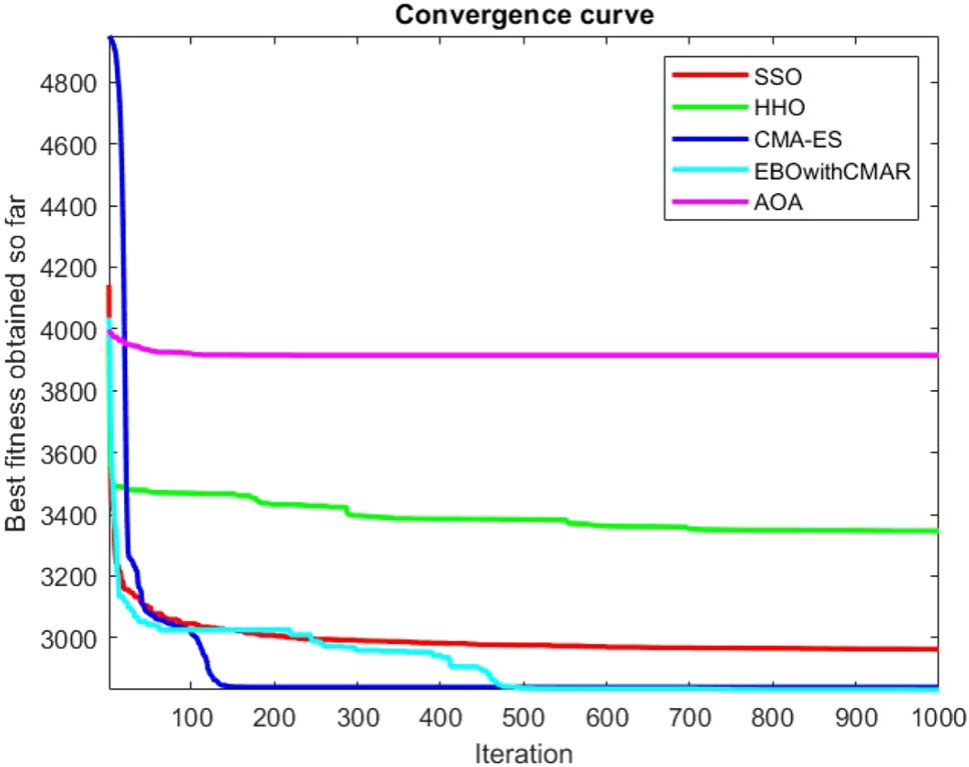
ComplexTest function 24 (Dimension = 30).

**Figure 77:**
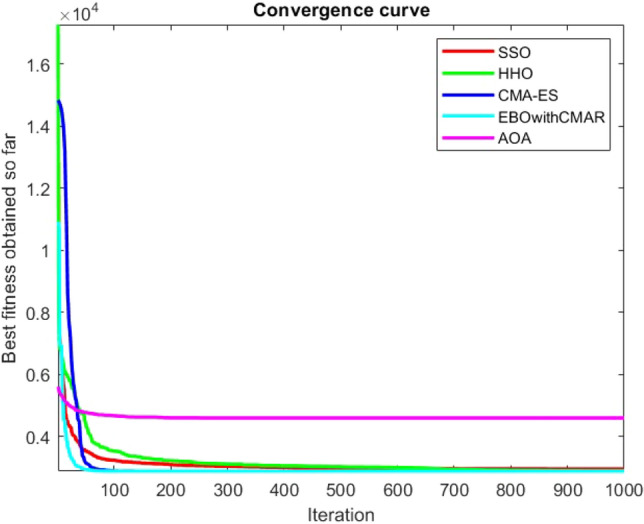
ComplexTest function 25 (Dimension = 30).

**Figure 78:**
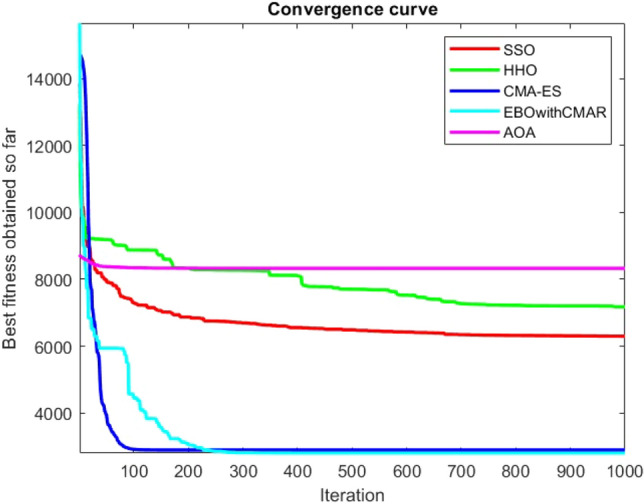
ComplexTest function 26 (Dimension = 30).

**Figure 79:**
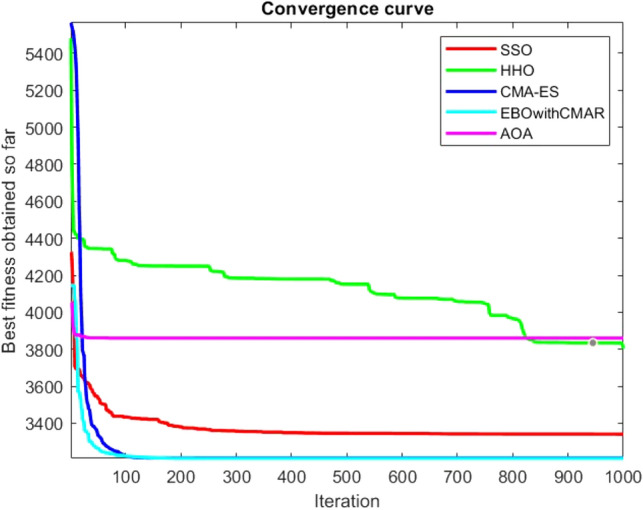
ComplexTest function27 (Dimension = 30).

**Figure 80:**
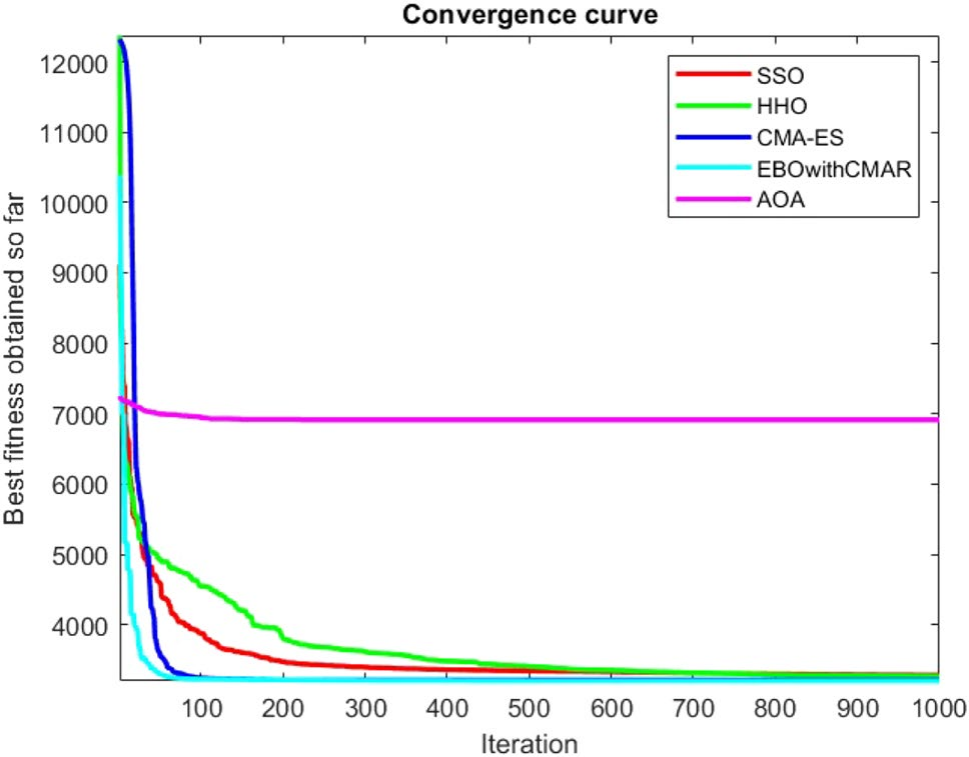
ComplexTest function 28 (Dimension = 30).

**Figure 81:**
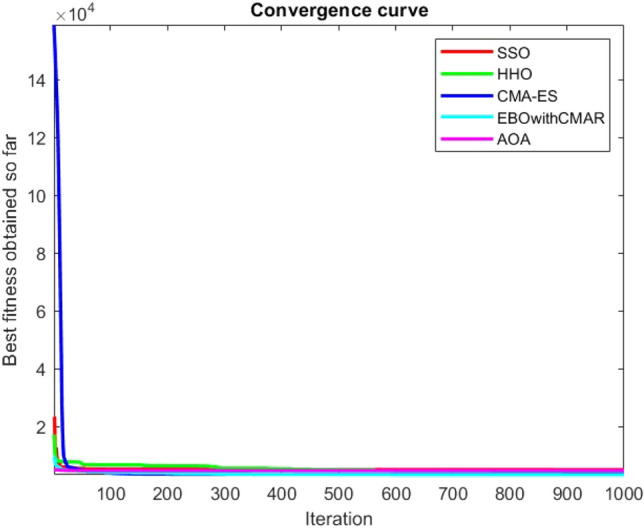
ComplexTest function 29 (Dimension = 30).

**Figure 82:**
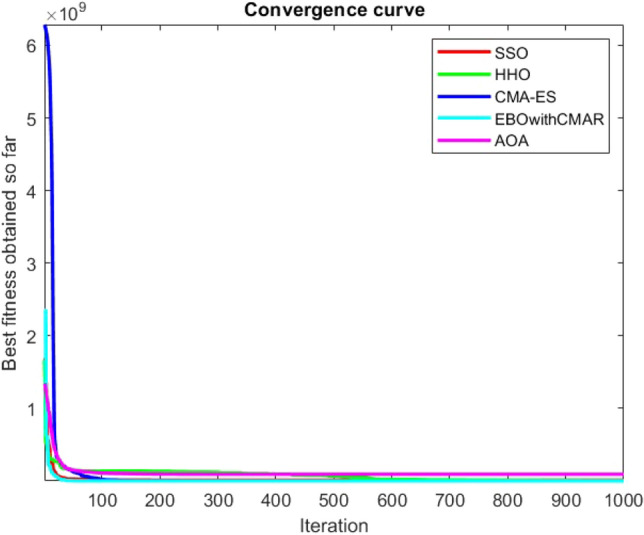
ComplexTest function 30 (Dimension = 30).

**Figure 83:**
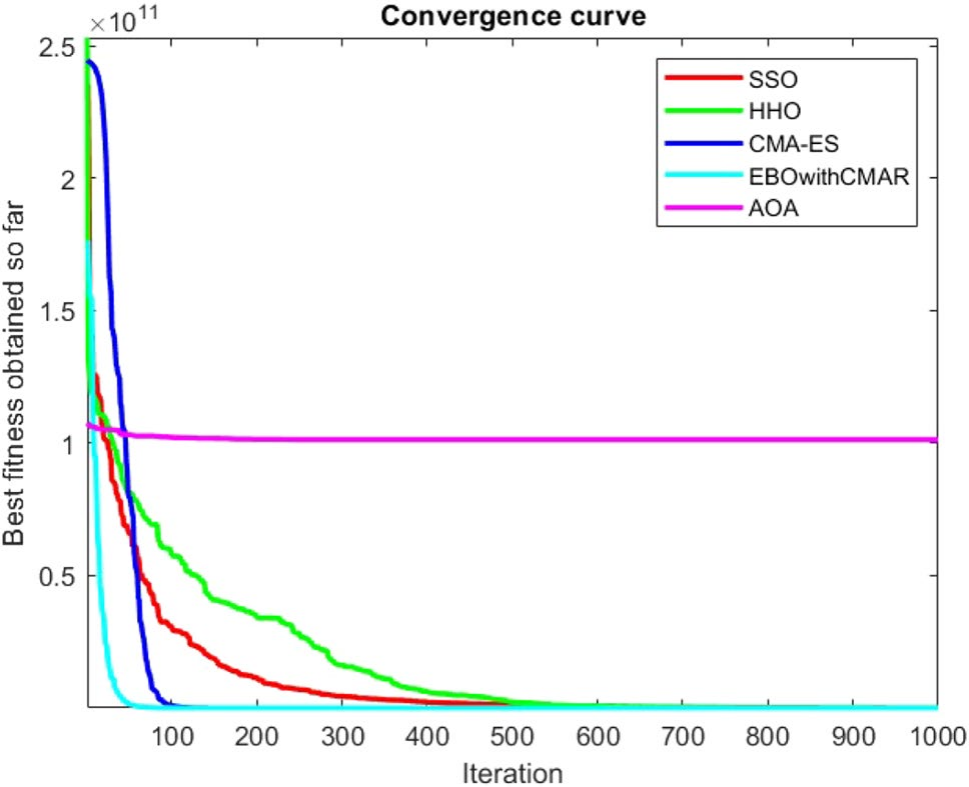
ComplexTest function 1 (Dimension = 50).

**Figure 84:**
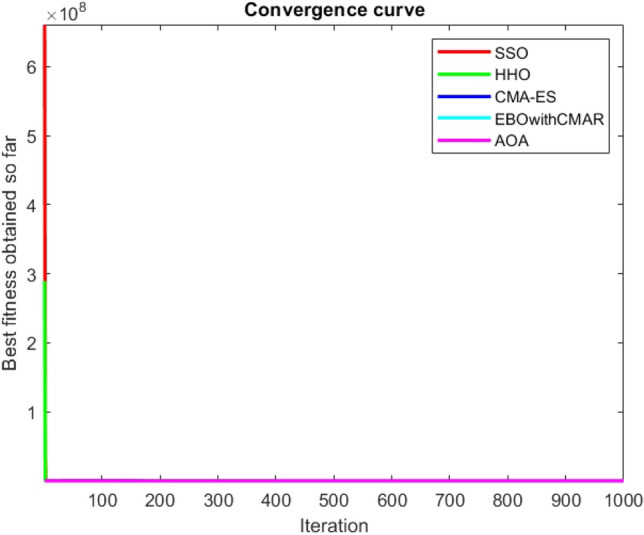
ComplexTest function 3 (Dimension = 50).

**Figure 85:**
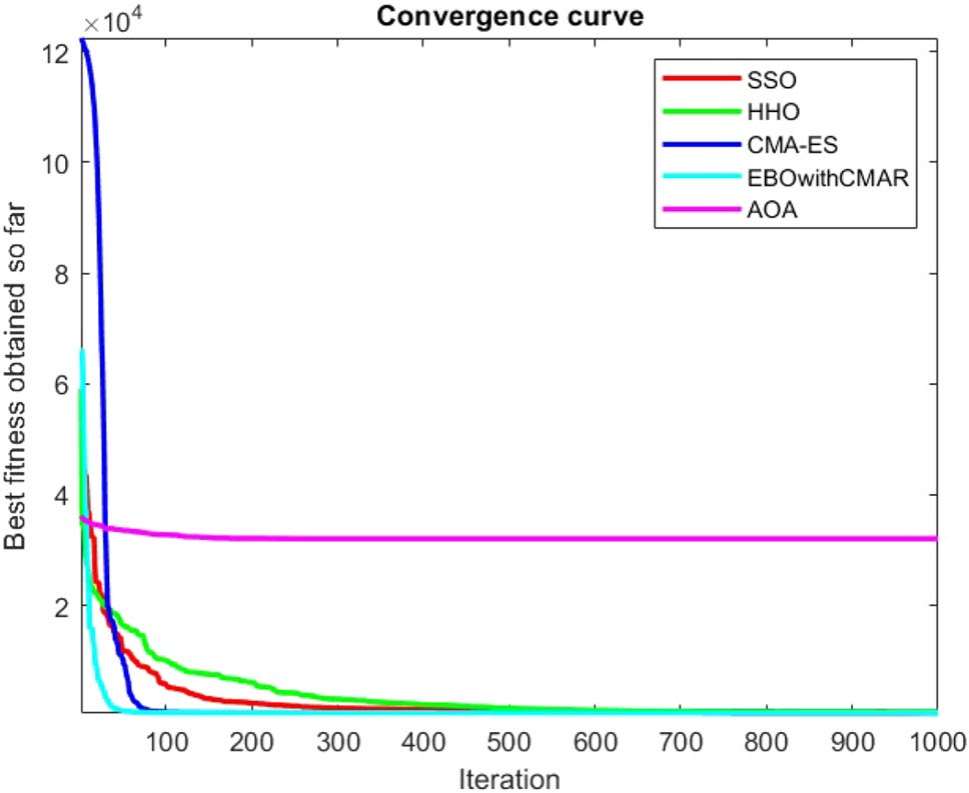
ComplexTest function 4 (Dimension = 50).

**Figure 86:**
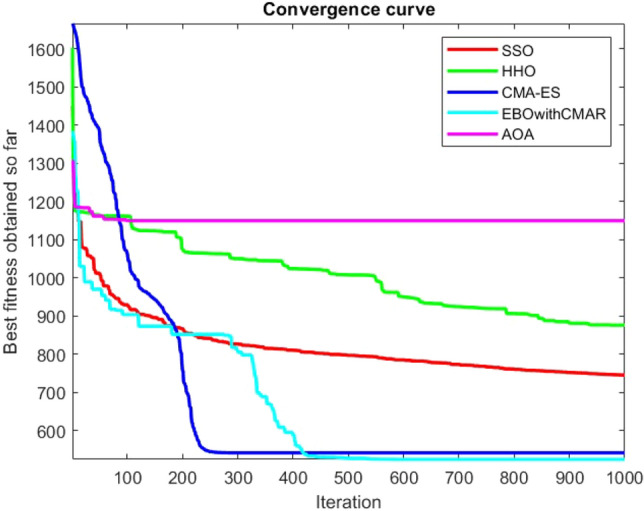
ComplexTest function 5 (Dimension = 50).

**Figure 87:**
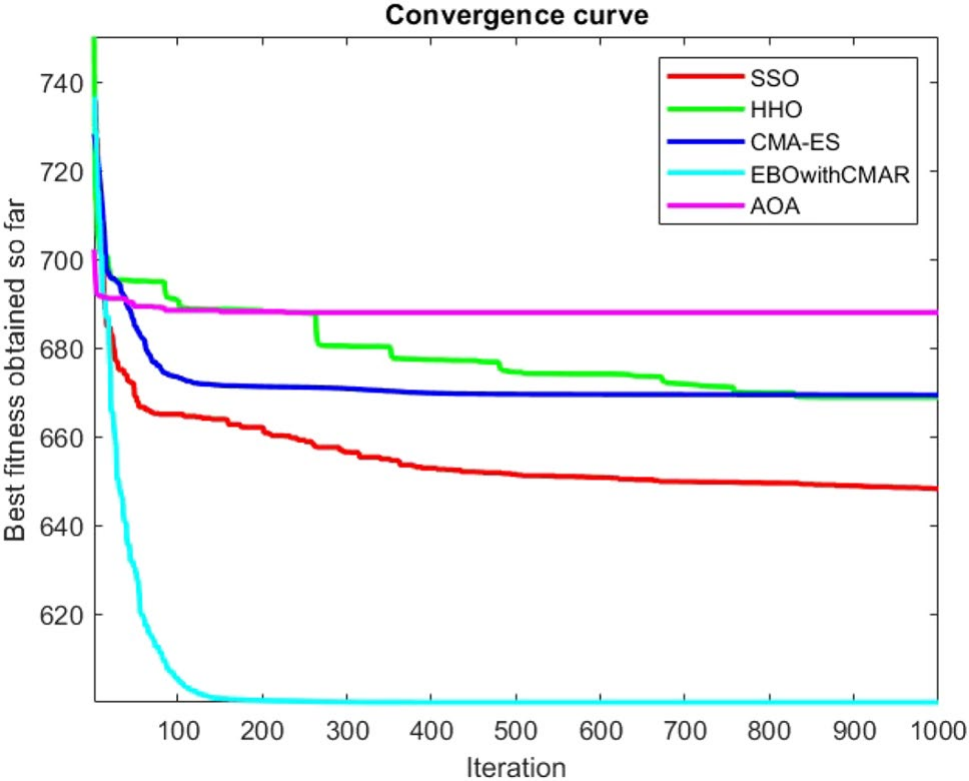
ComplexTest function 6 (Dimension = 50).

**Figure 88:**
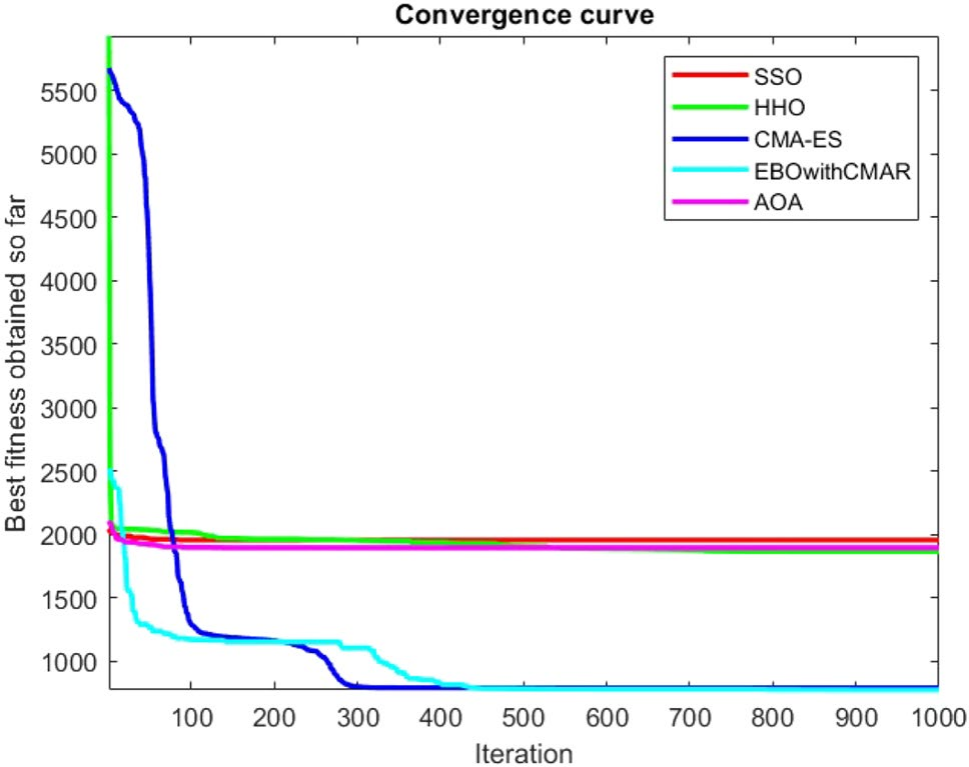
ComplexTest function 7 (Dimension = 50).

**Figure 89:**
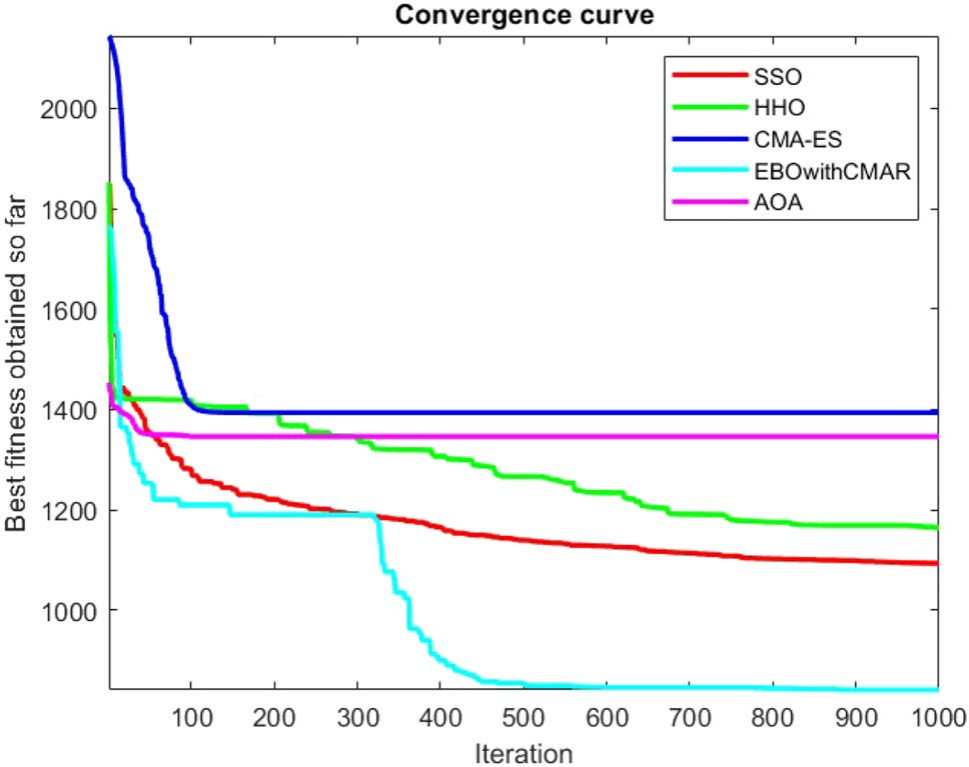
ComplexTest function 8 (Dimension = 50).

**Figure 90:**
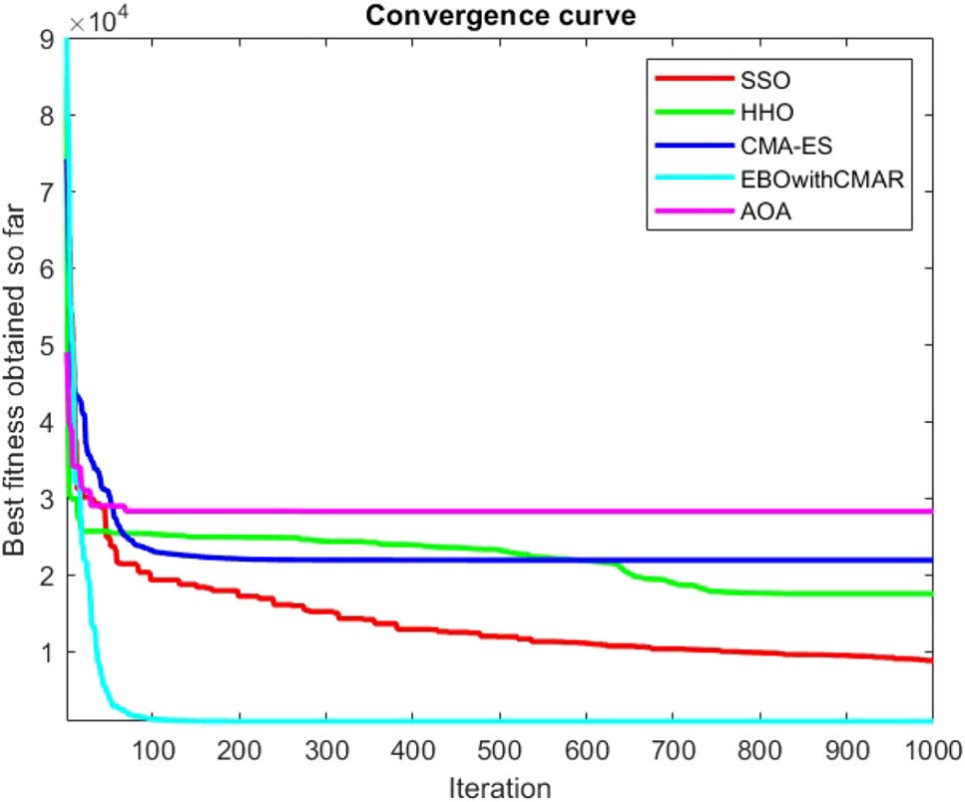
ComplexTest function 9 (Dimension = 50).

**Figure 91:**
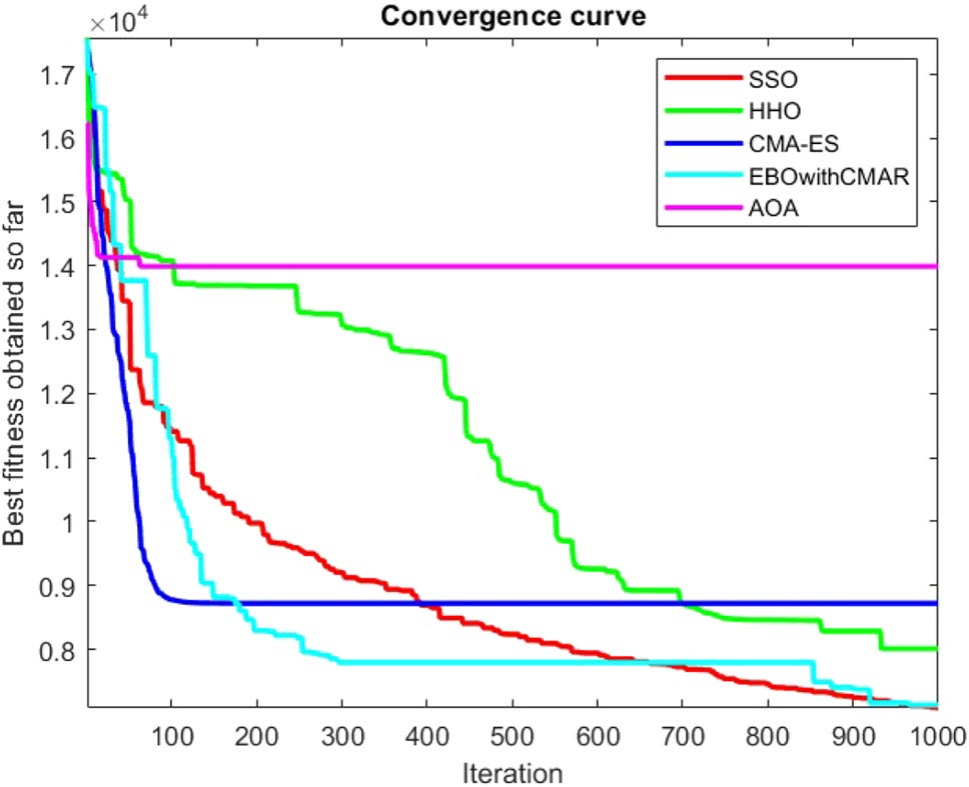
ComplexTest function 10 (Dimension = 50).

**Figure 92:**
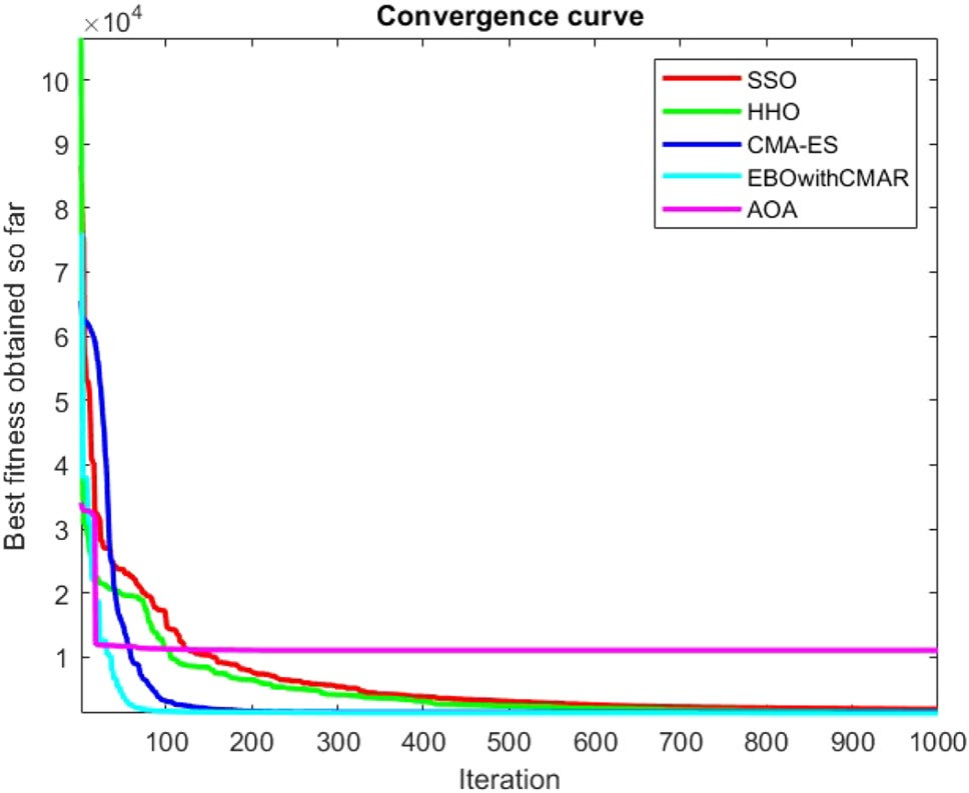
ComplexTest function 11 (Dimension = 50).

**Figure 93:**
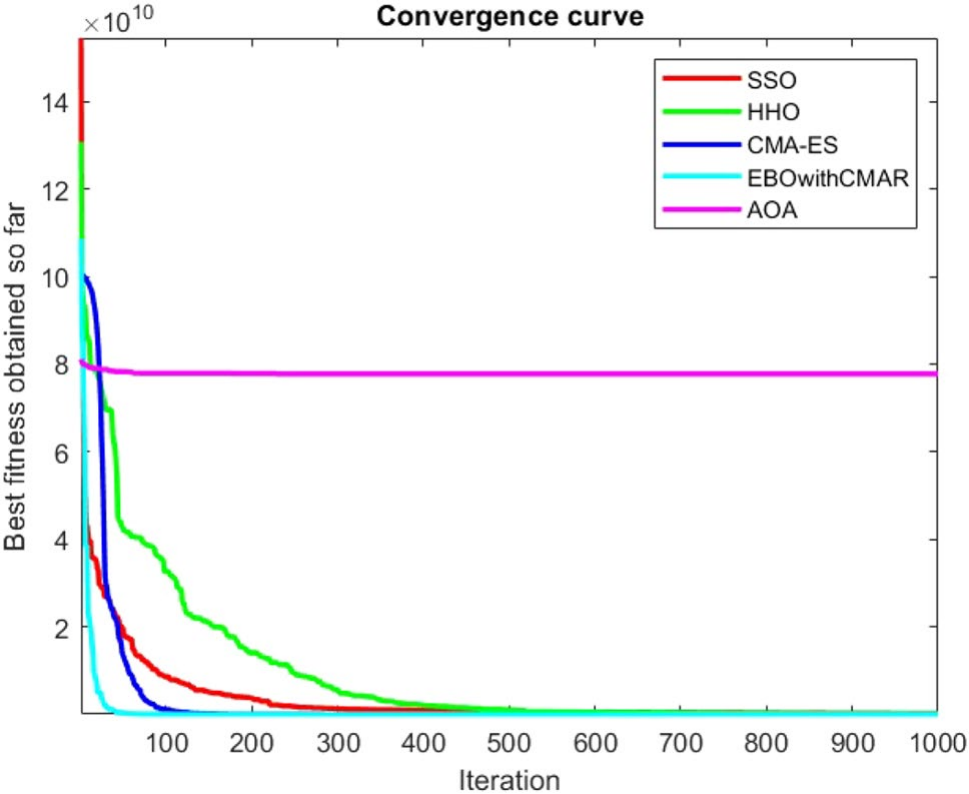
ComplexTest function 12 (Dimension = 50).

**Figure 94:**
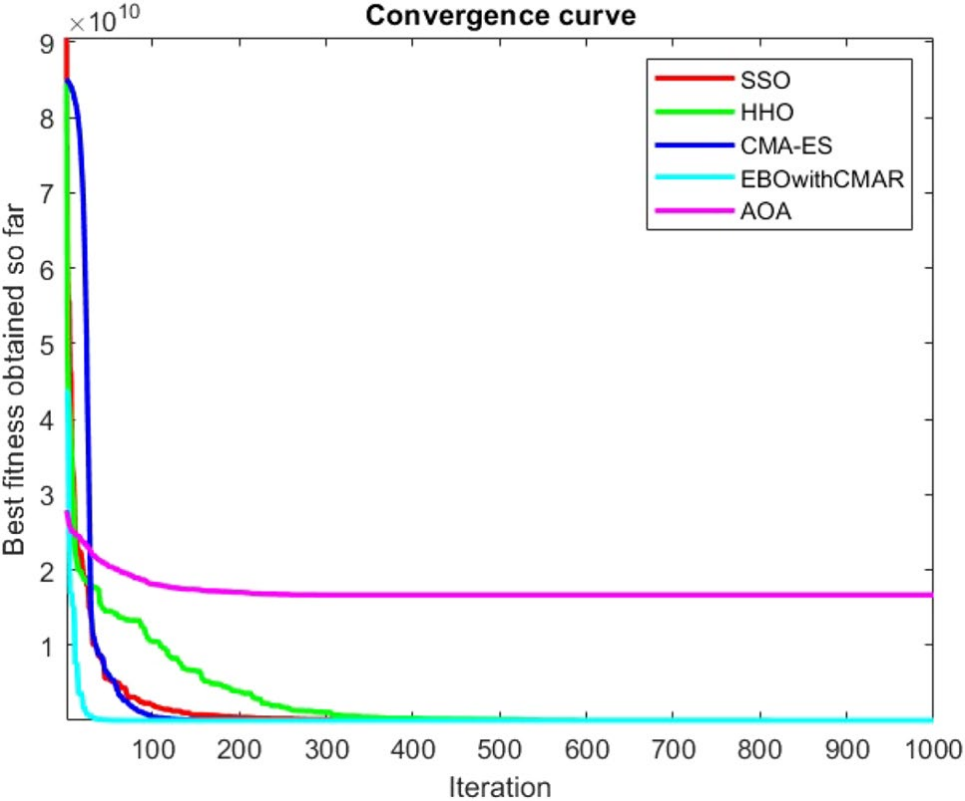
ComplexTest function 13 (Dimension = 50).

**Figure 95:**
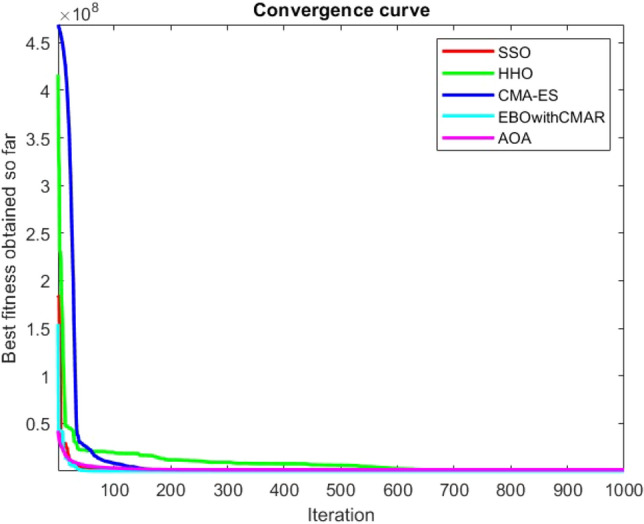
ComplexTest function 14 (Dimension = 50).

**Figure 96:**
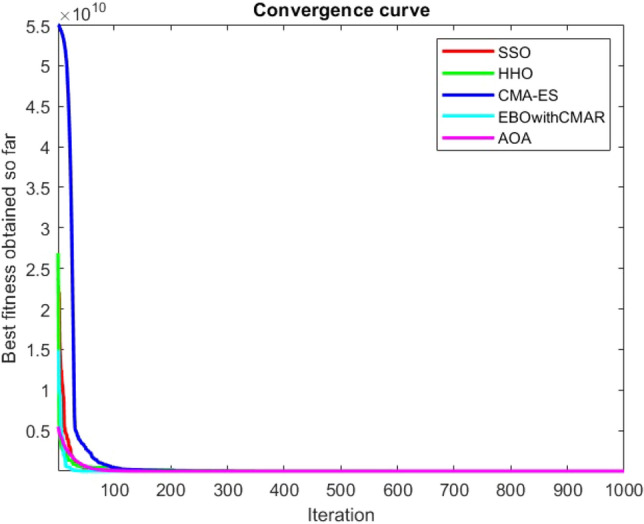
ComplexTest function 15 (Dimension = 50).

**Figure 97:**
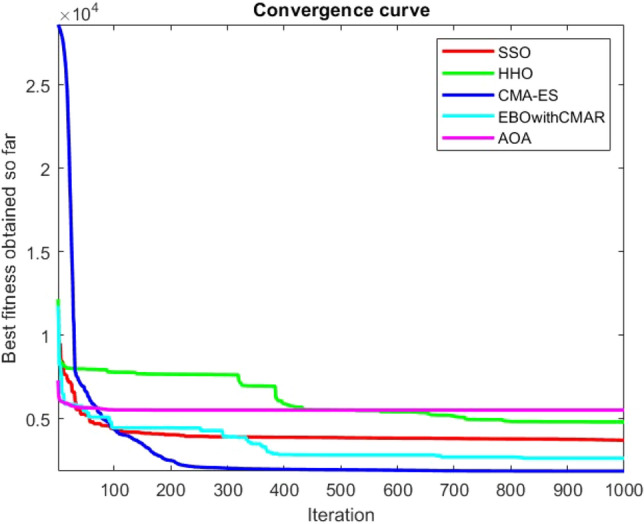
ComplexTest function 16 (Dimension = 50).

**Figure 98:**
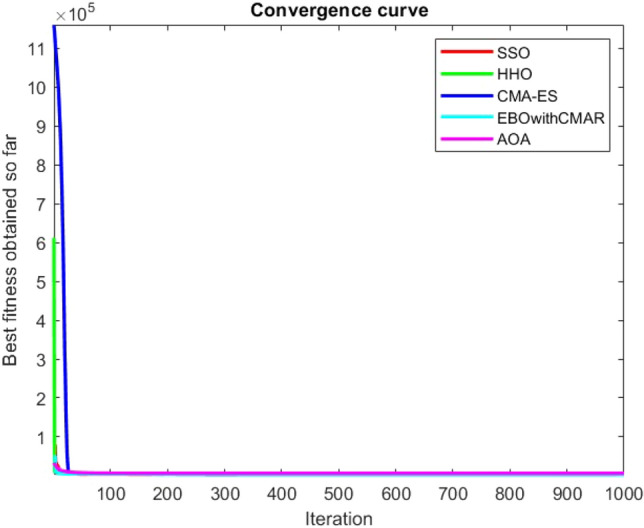
ComplexTest function 17 (Dimension = 50).

**Figure 99:**
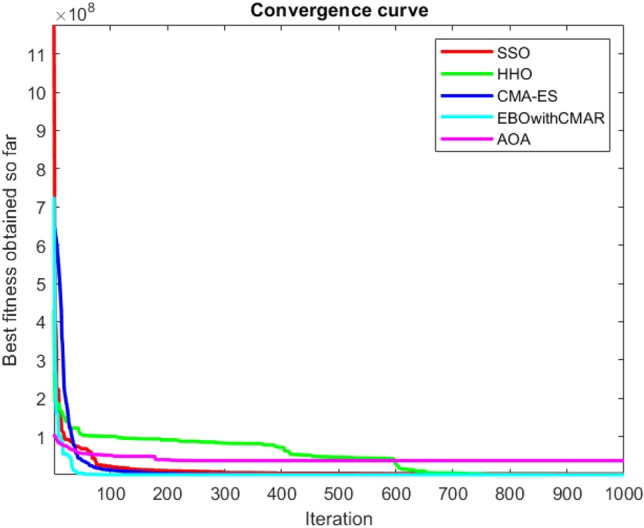
ComplexTest function 18 (Dimension = 50).

**Figure 100:**
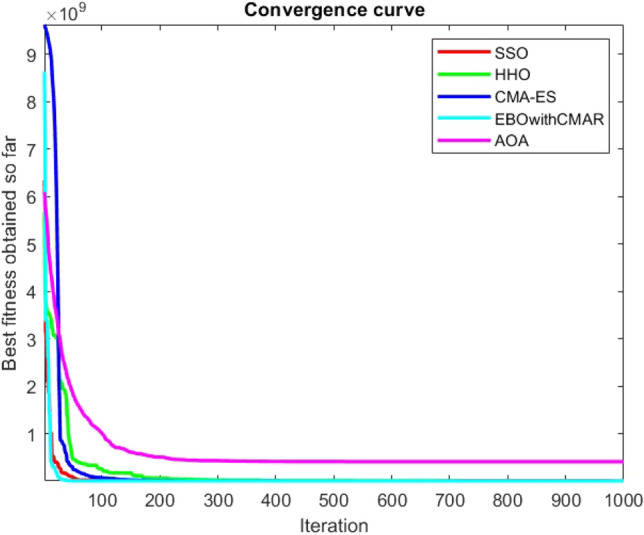
ComplexTest function 19 (Dimension = 50).

**Figure 101:**
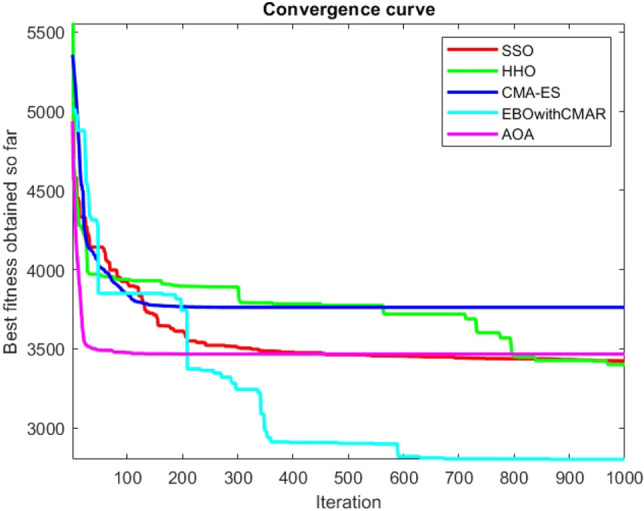
ComplexTest function 20 (Dimension = 50).

**Figure 102:**
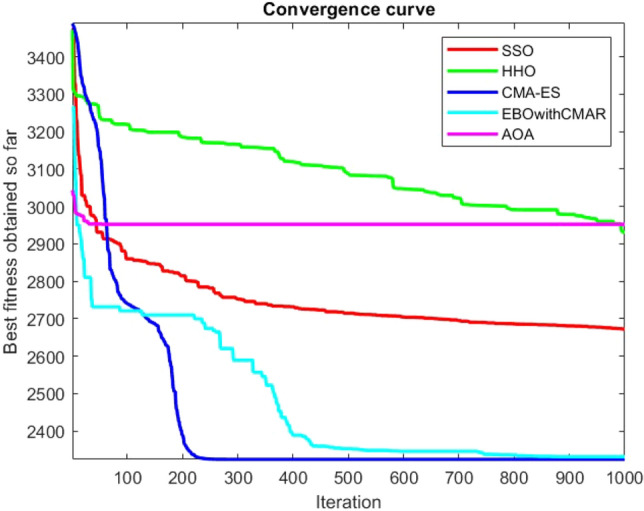
ComplexTest function 21 (Dimension = 50).

**Figure 103:**
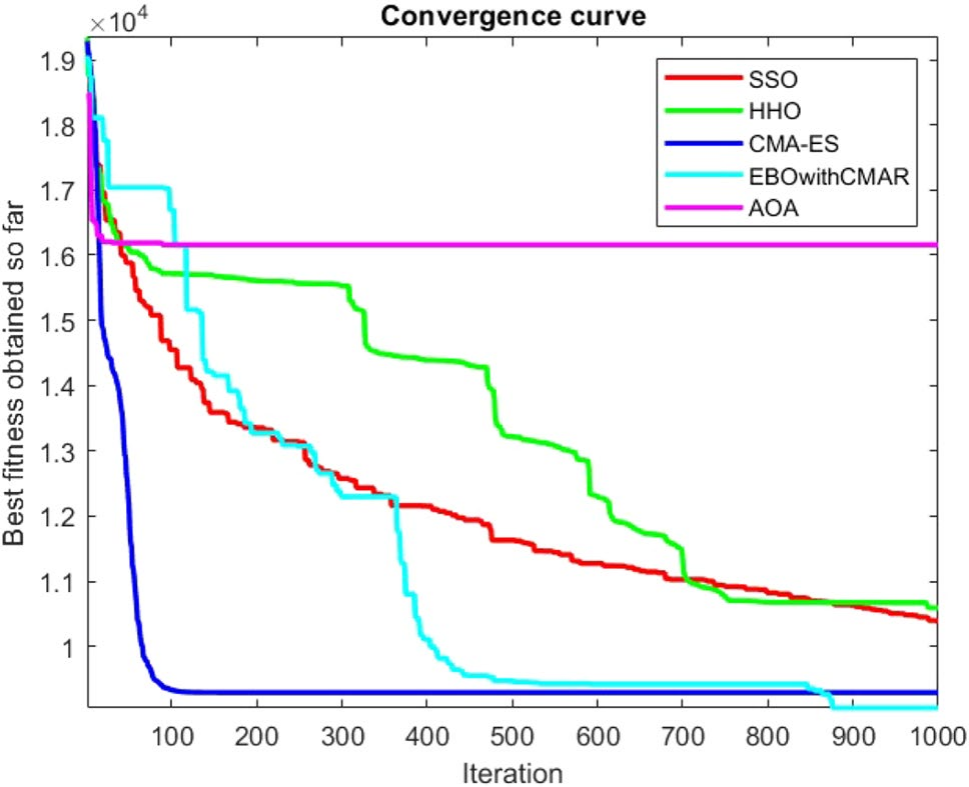
ComplexTest function 22 (Dimension = 50).

**Figure 104:**
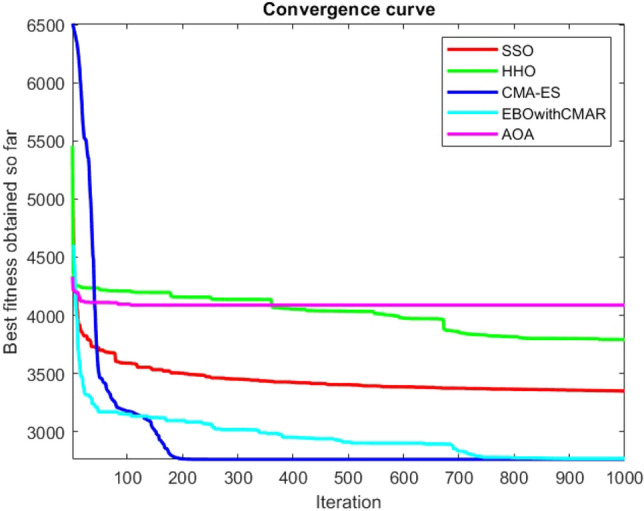
ComplexTest function 23 (Dimension = 50).

**Figure 105:**
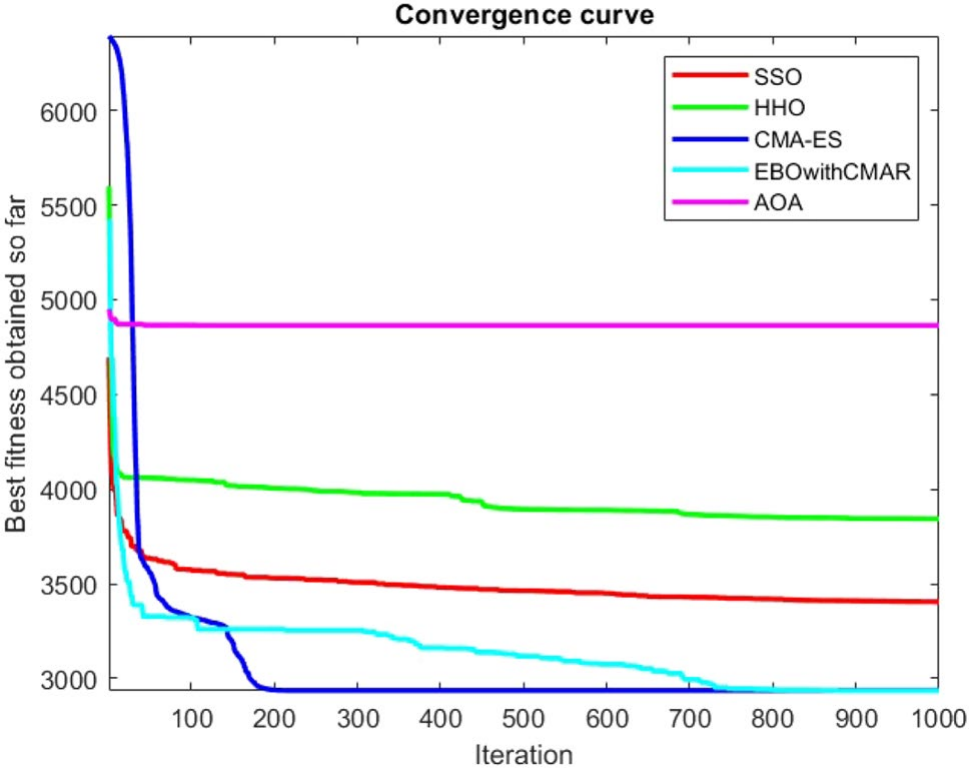
ComplexTest function 24 (Dimension = 50).

**Figure 106:**
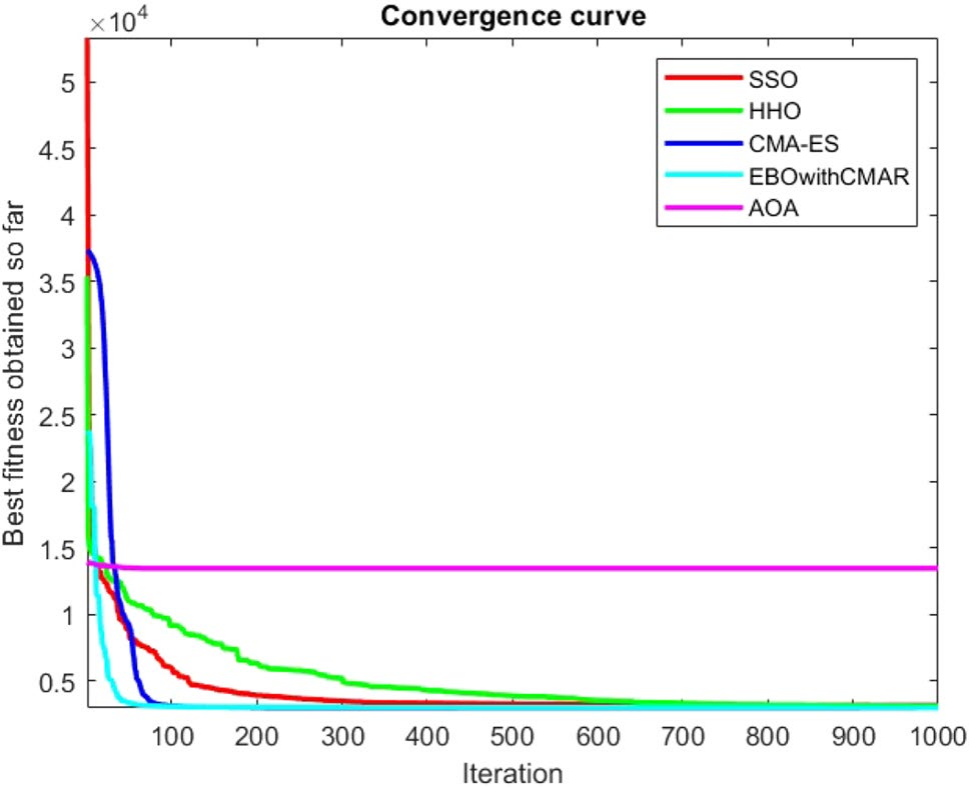
ComplexTest function 25 (Dimension = 50).

**Figure 107:**
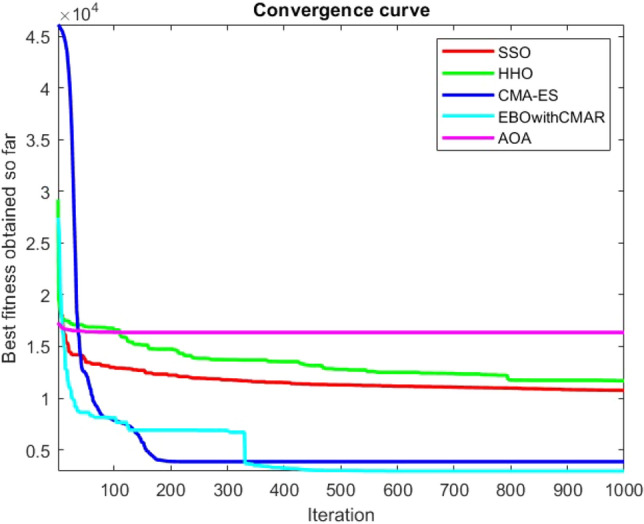
ComplexTest function 26 (Dimension = 50).

**Figure 108:**
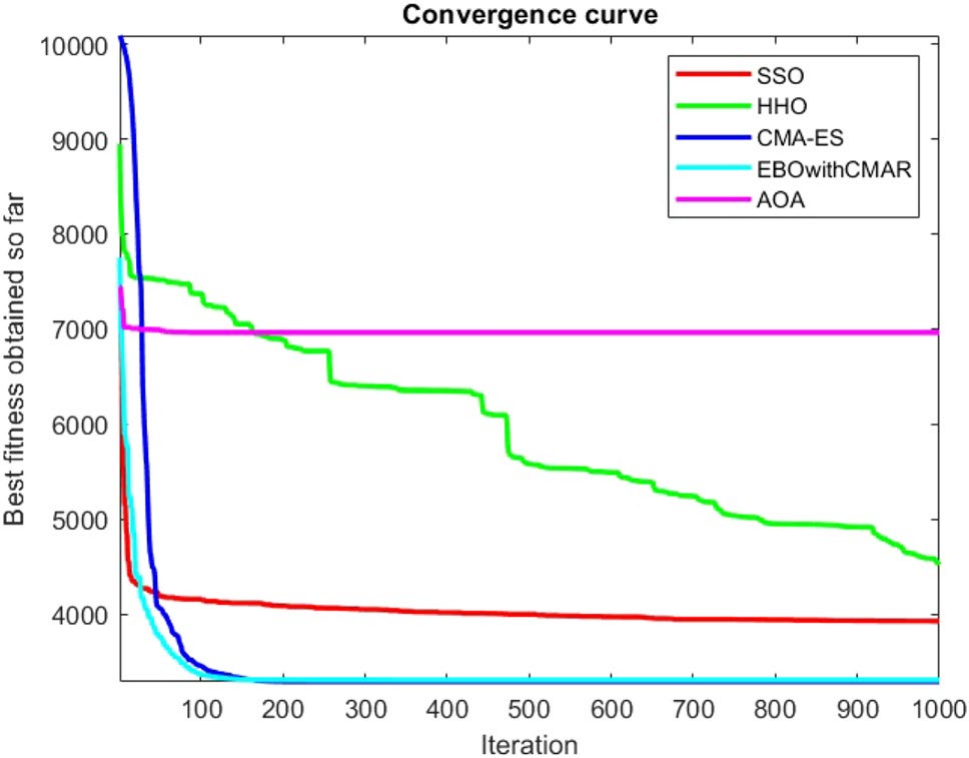
ComplexTest function 27 (Dimension = 50).

**Figure 109:**
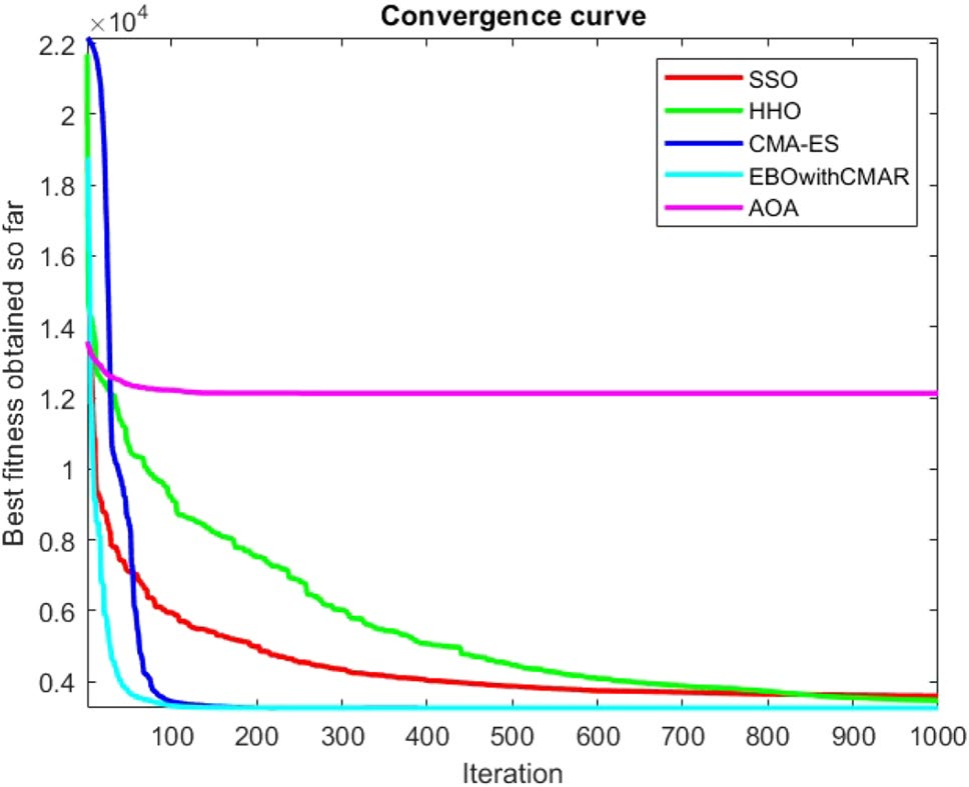
ComplexTest function 28 (Dimension = 50).

**Figure 110:**
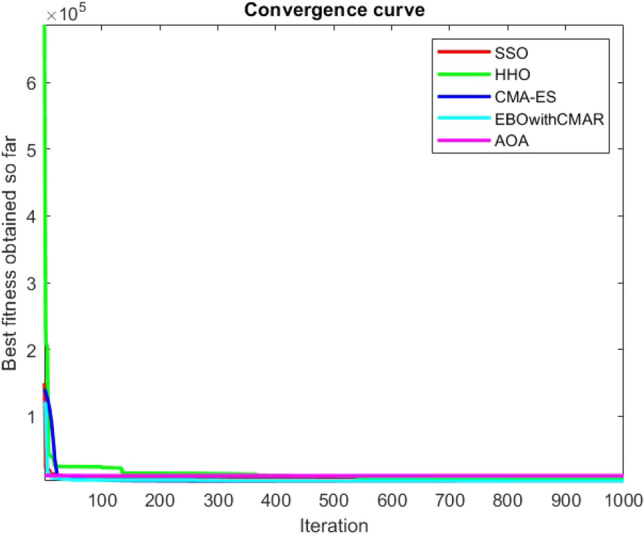
ComplexTest function 29 (Dimension = 50).

**Figure 111:**
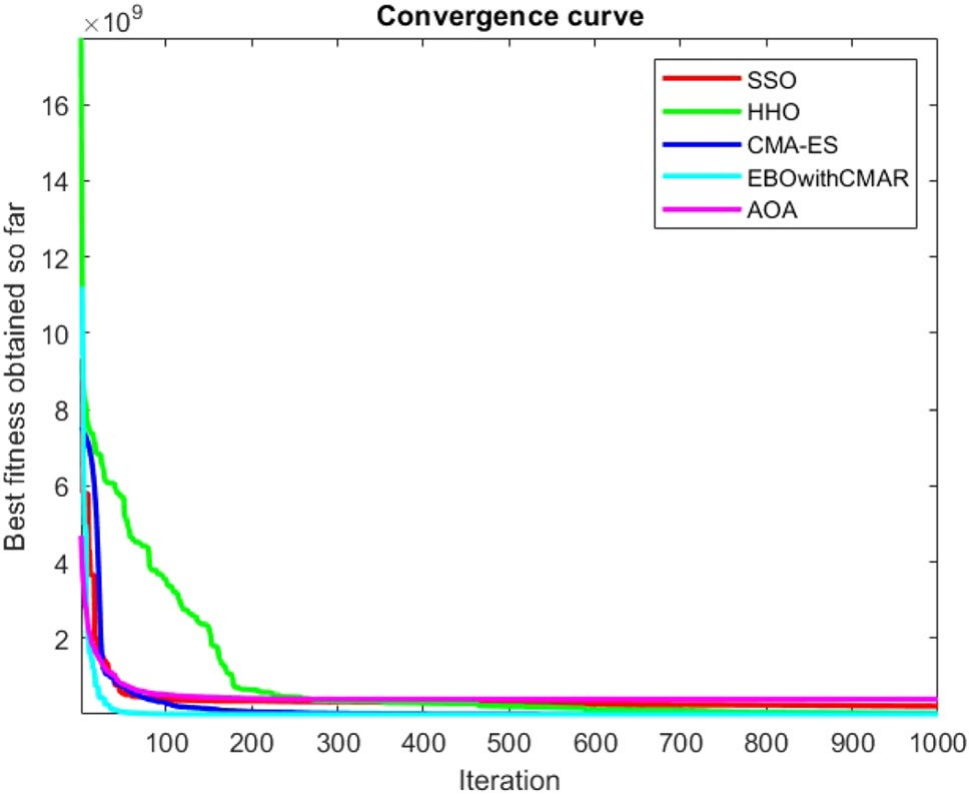
ComplexTest function 30 (Dimension = 50).

**Figure 112:**
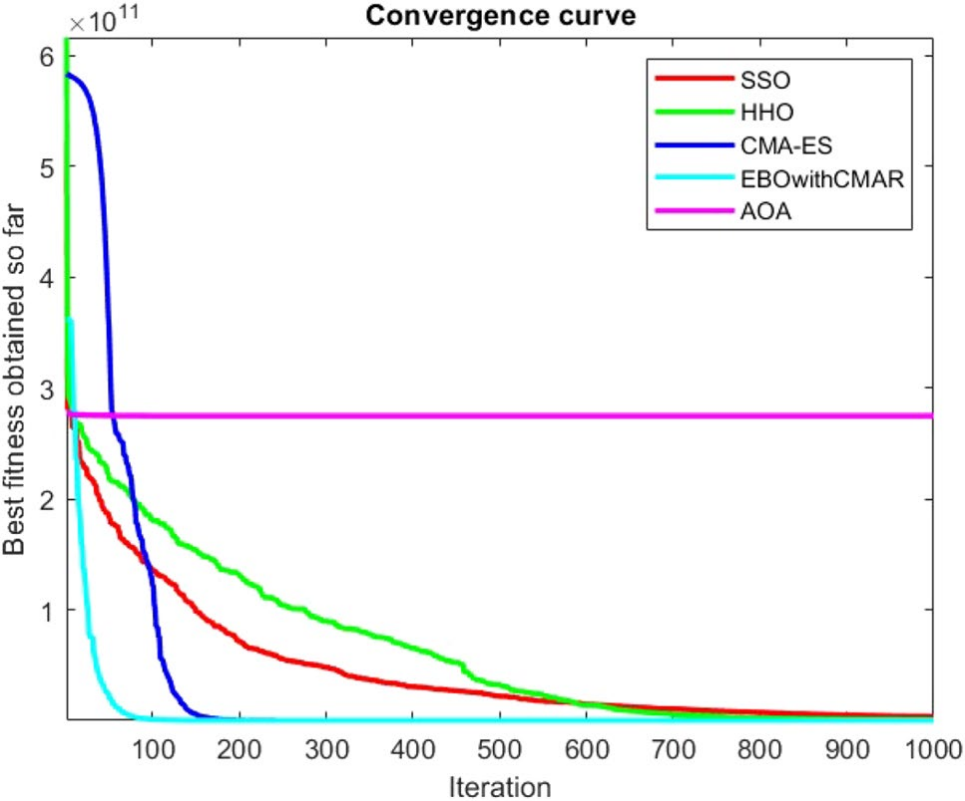
ComplexTest function 1 (Dimension = 100).

**Figure 113:**
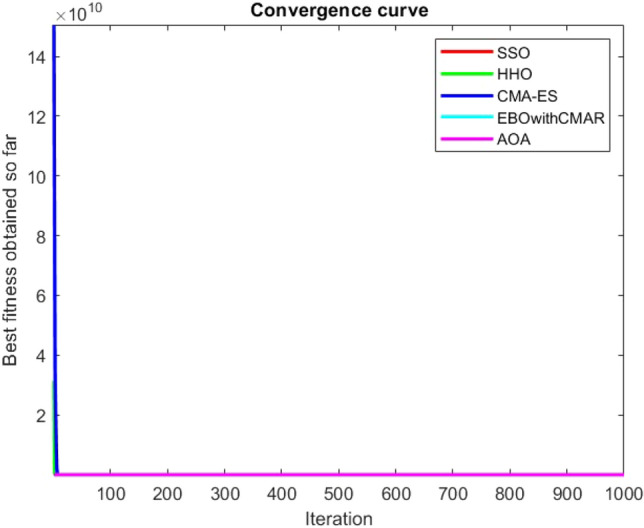
ComplexTest function 3 (Dimension = 100).

**Figure 114:**
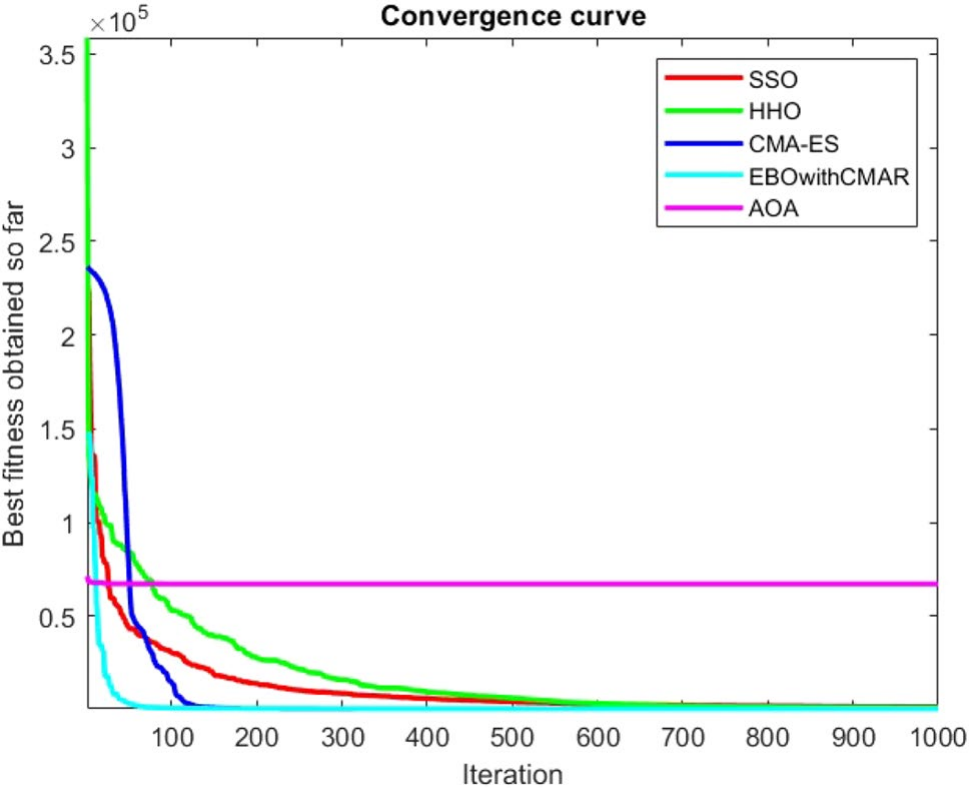
ComplexTest function 4 (Dimension = 100).

**Figure 115:**
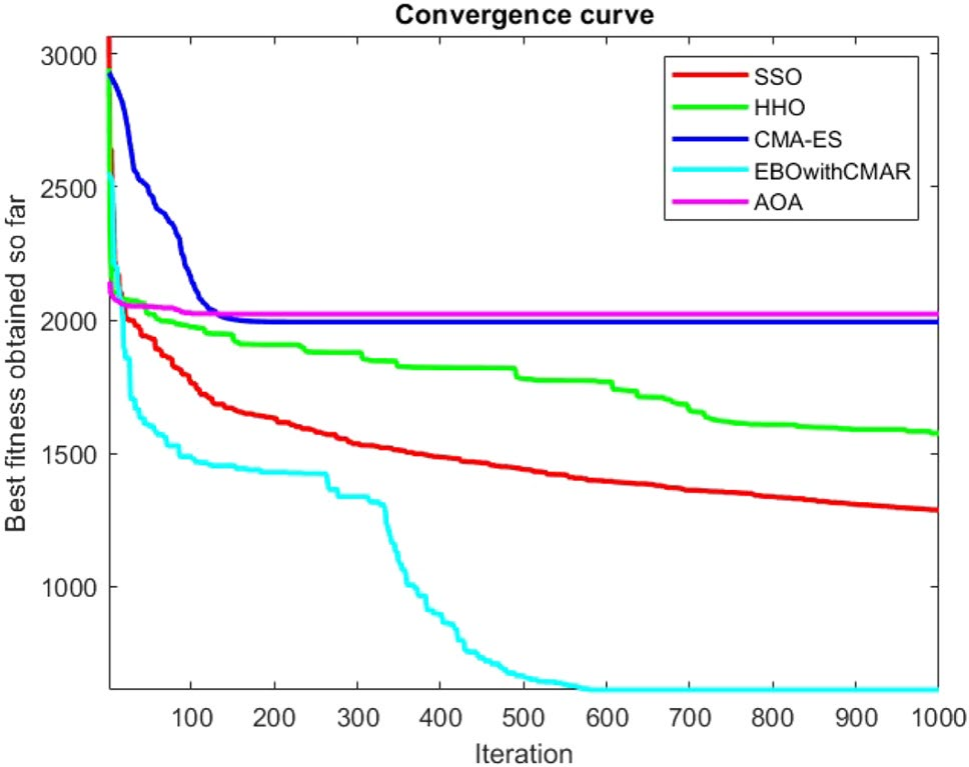
ComplexTest function 5 (Dimension = 100).

**Figure 116:**
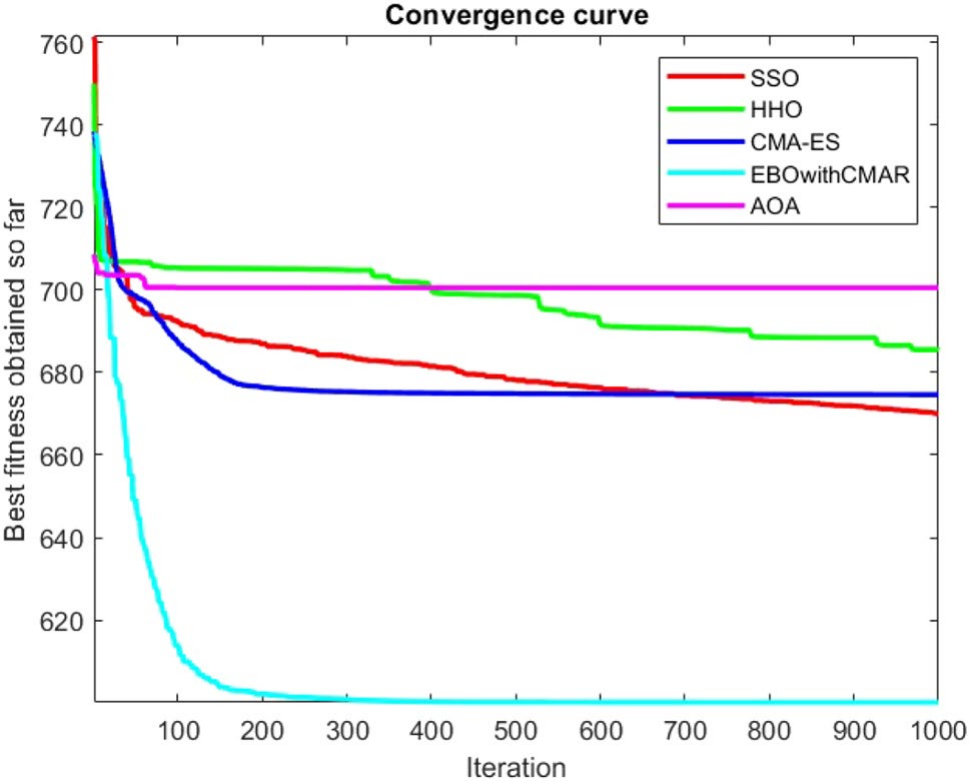
ComplexTest function 6 (Dimension = 100).

**Figure 117:**
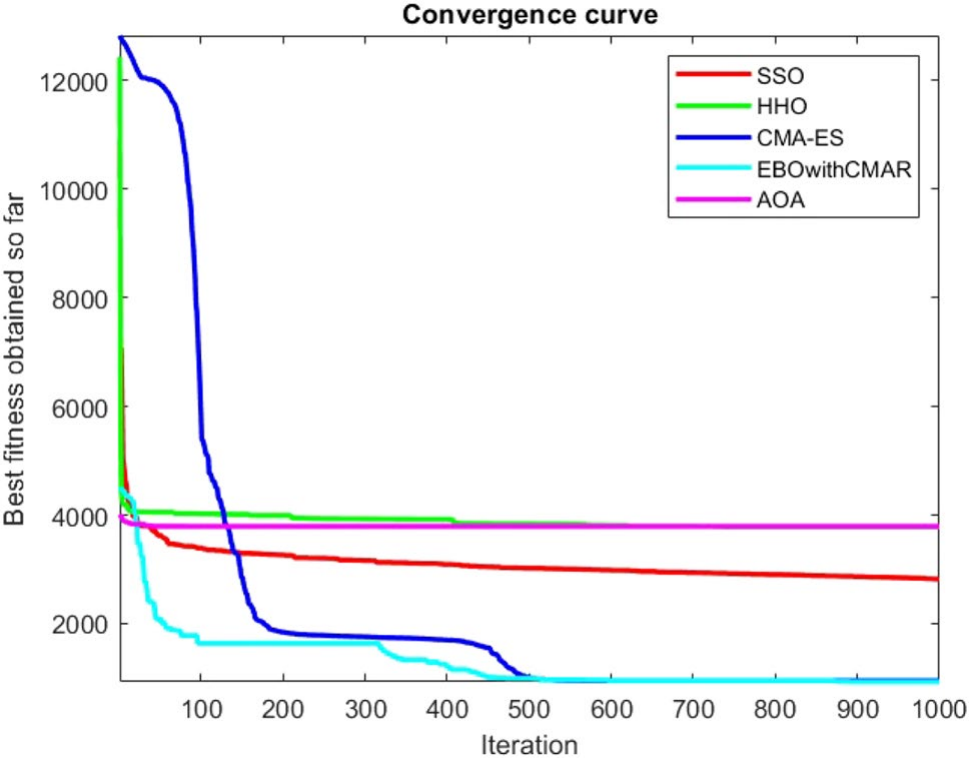
ComplexTest function 7 (Dimension = 100).

**Figure 118:**
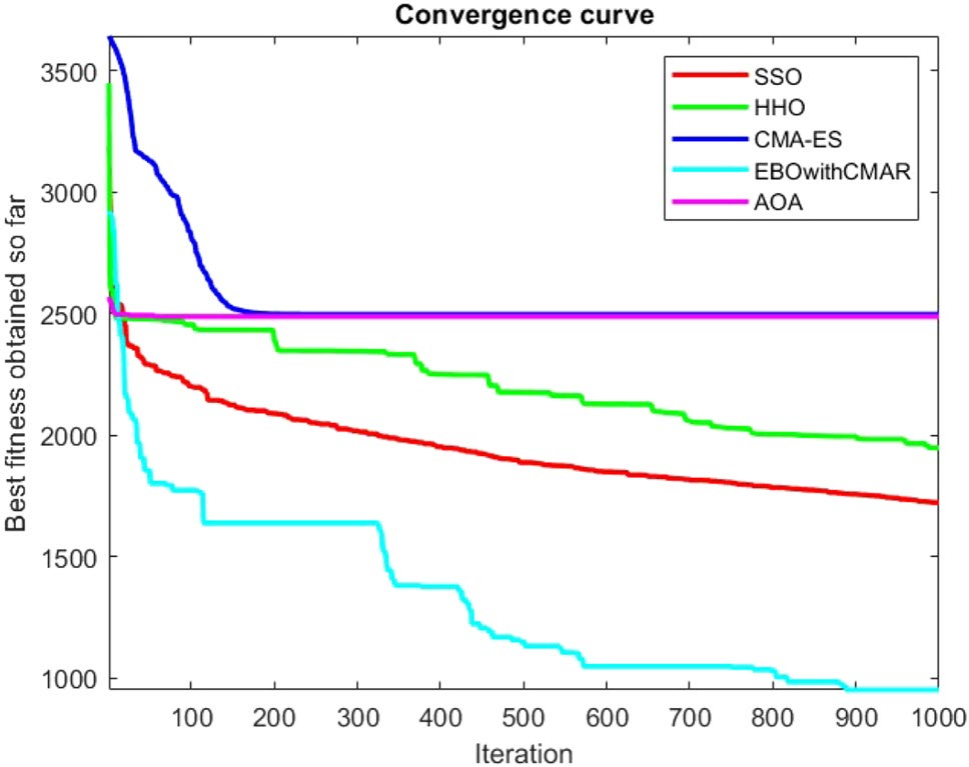
ComplexTest function 8 (Dimension = 100).

**Figure 119:**
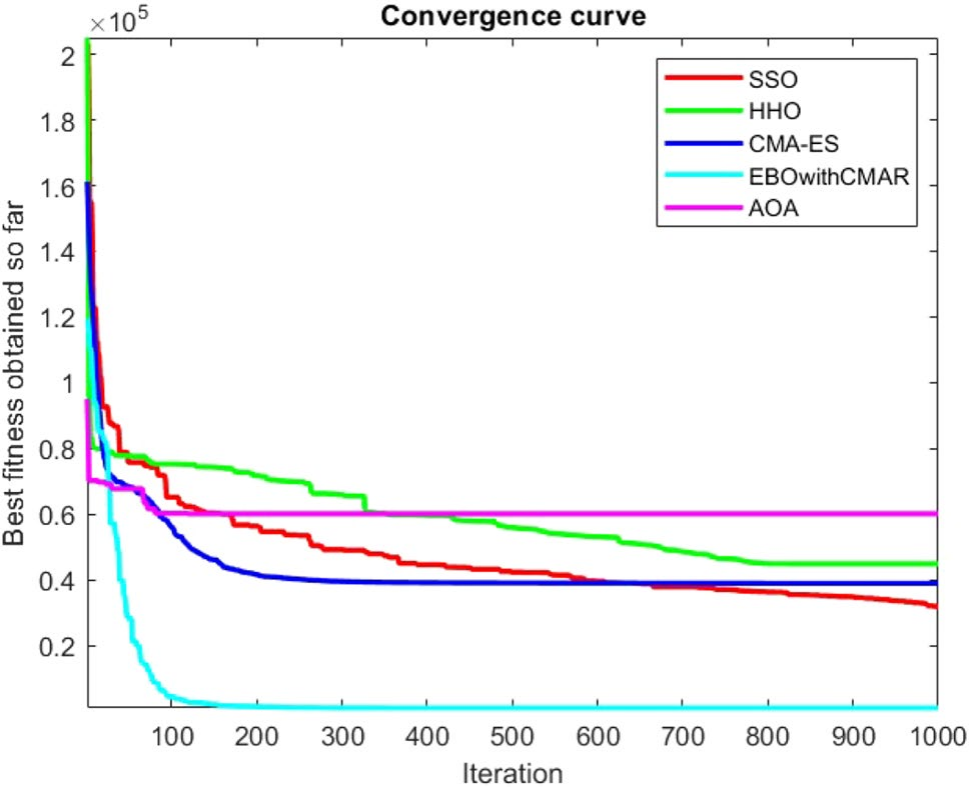
ComplexTest function 9 (Dimension = 100).

**Figure 120:**
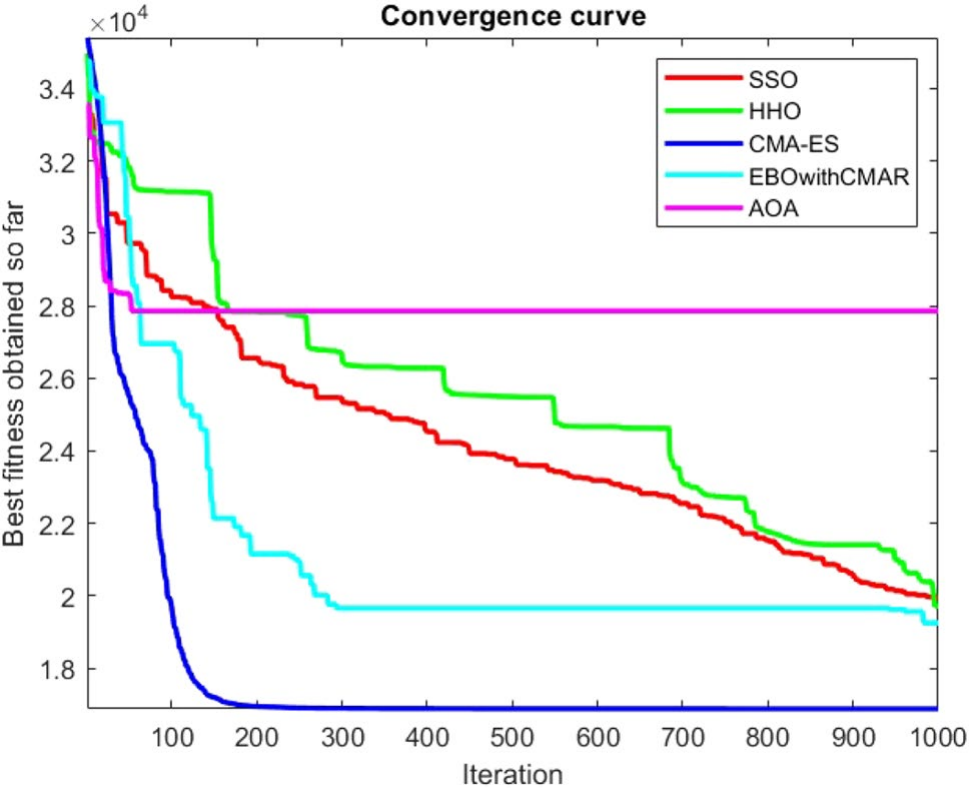
ComplexTest function 10 (Dimension = 100).

**Figure 121:**
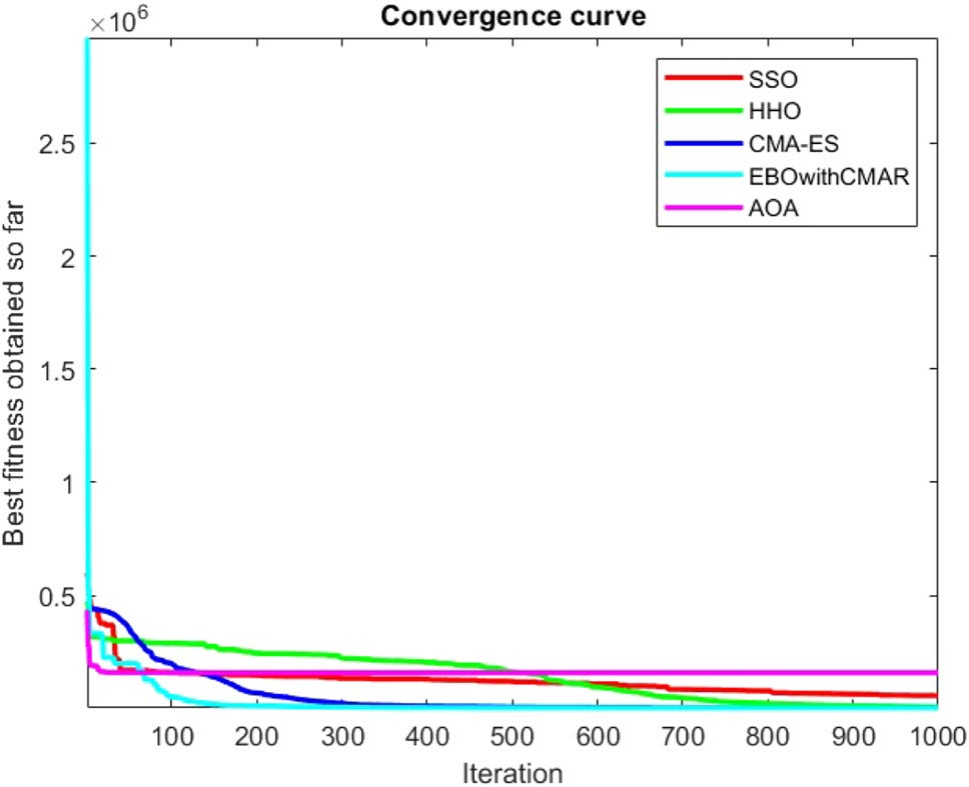
ComplexTest function 11 (Dimension = 100).

**Figure 122:**
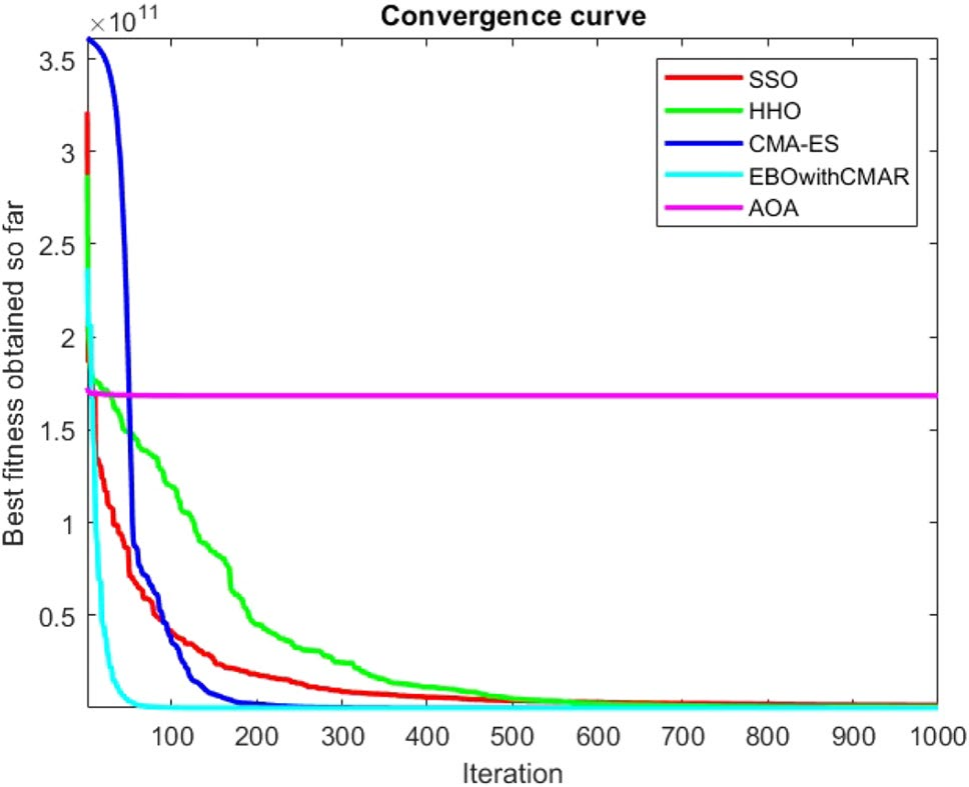
ComplexTest function 12 (Dimension = 100).

**Figure 123:**
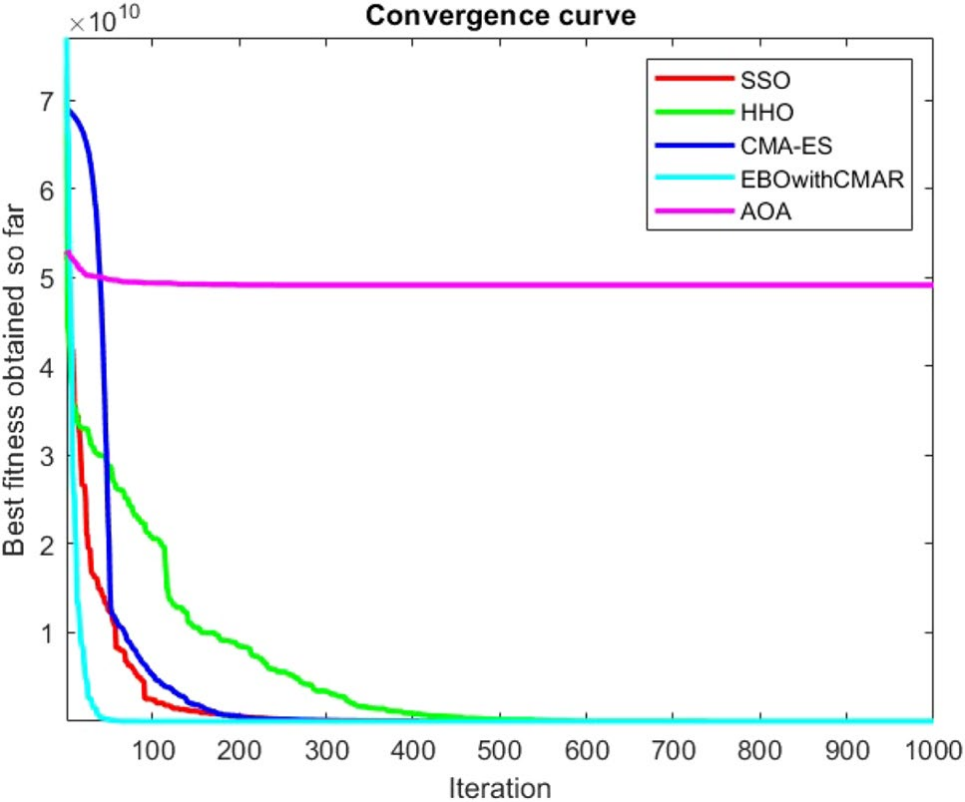
ComplexTest function 13 (Dimension = 100).

**Figure 124:**
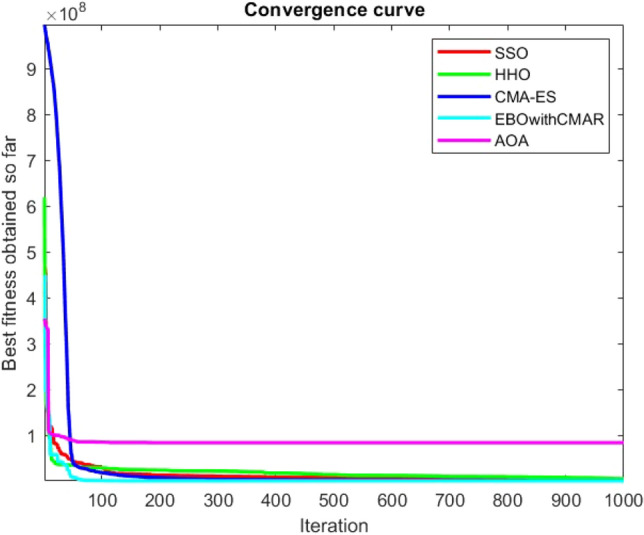
ComplexTest function 14 (Dimension = 100).

**Figure 125:**
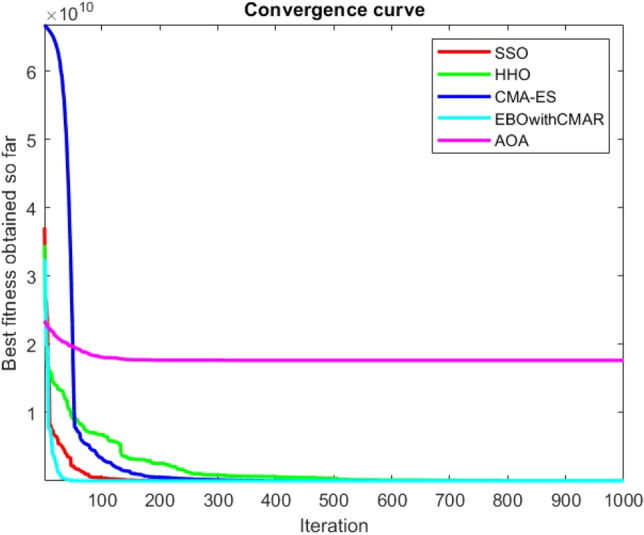
ComplexTest function 15 (Dimension = 100).

**Figure 126:**
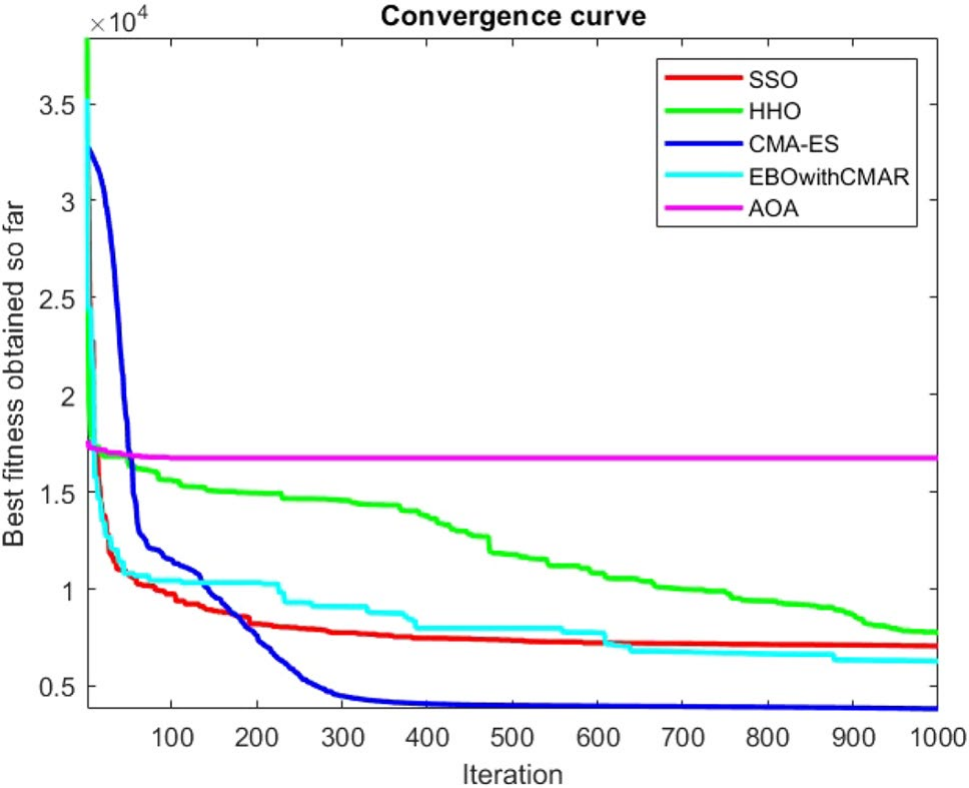
ComplexTest function 16 (Dimension = 100).

**Figure 127:**
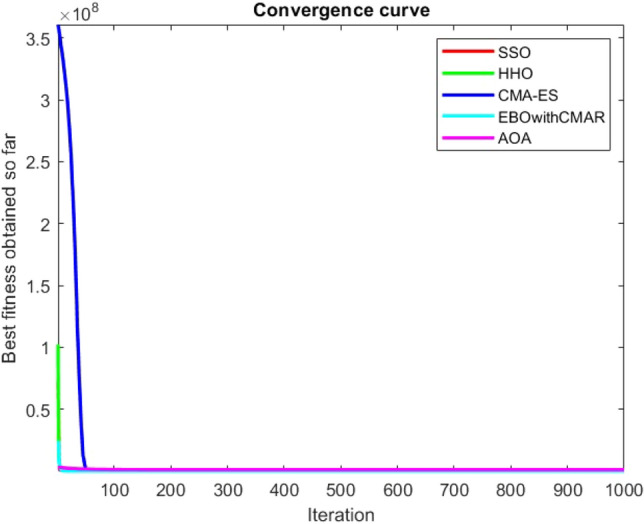
ComplexTest function 17 (Dimension = 100).

**Figure 128:**
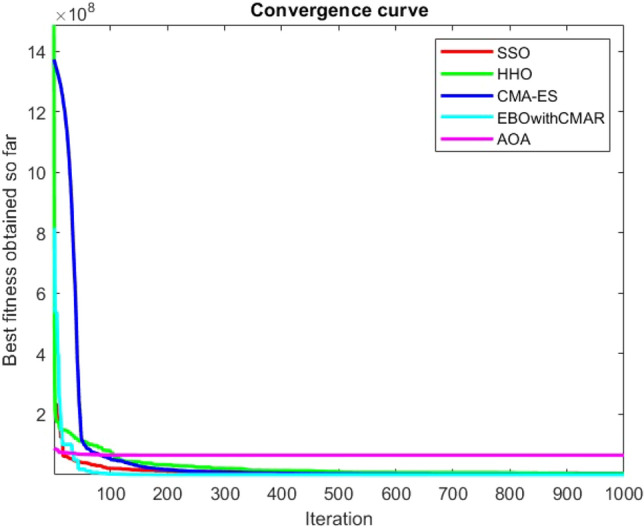
ComplexTest function 18 (Dimension = 100).

**Figure 129:**
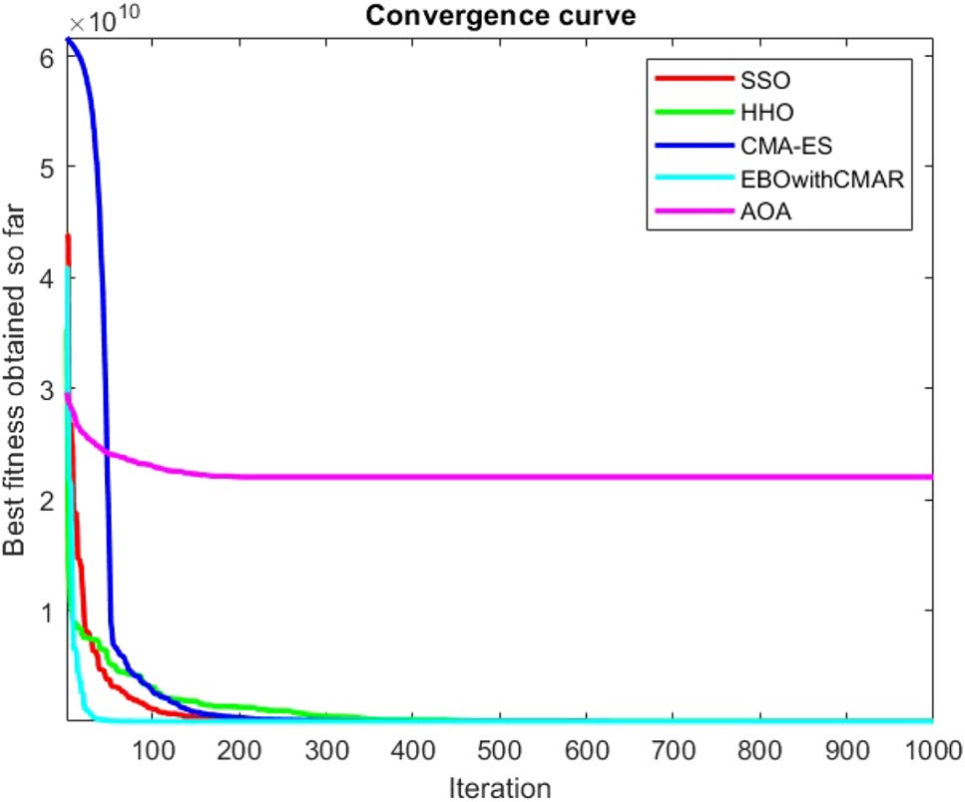
ComplexTest function 19 (Dimension = 100).

**Figure 130:**
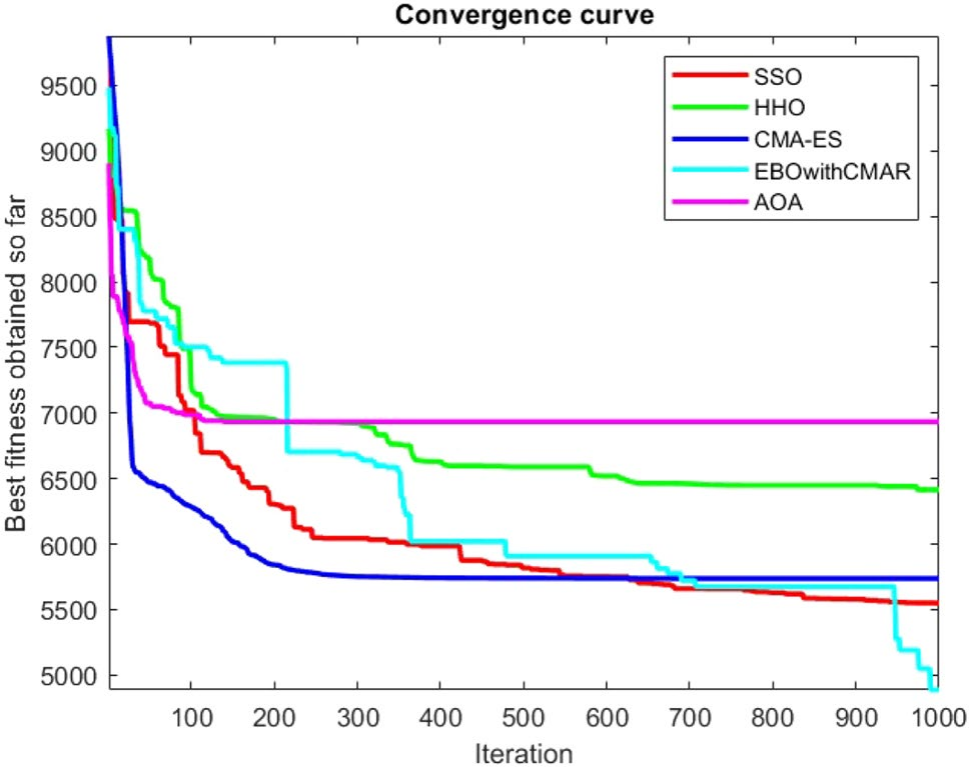
ComplexTest function 20 (Dimension = 100).

**Figure 131:**
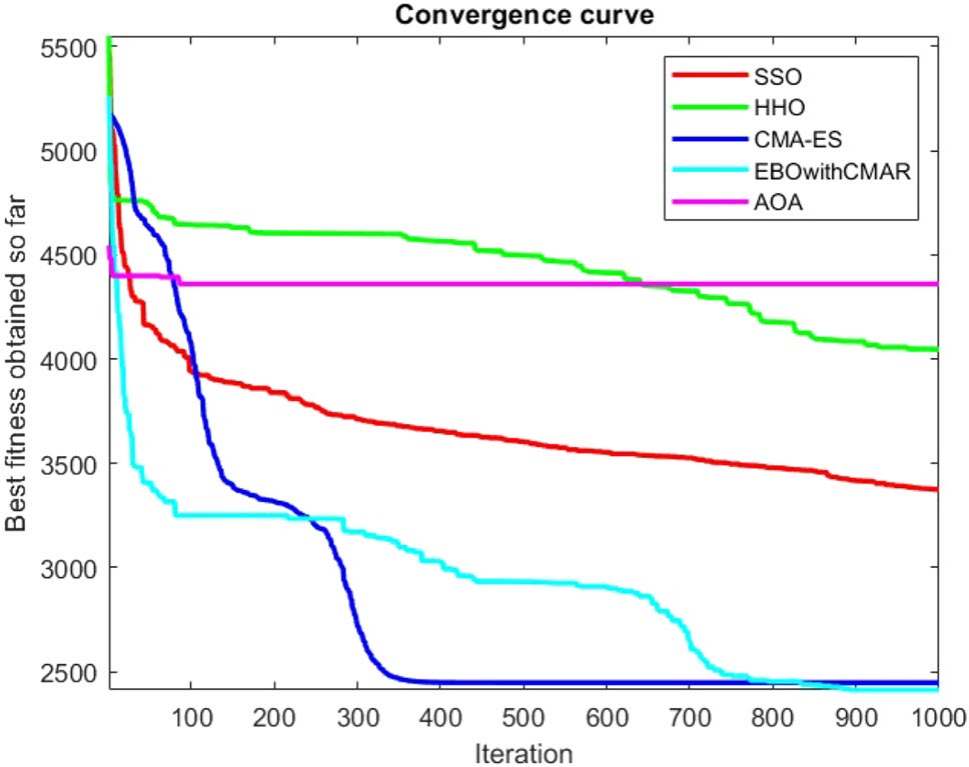
ComplexTest function 21 (Dimension = 100).

**Figure 132:**
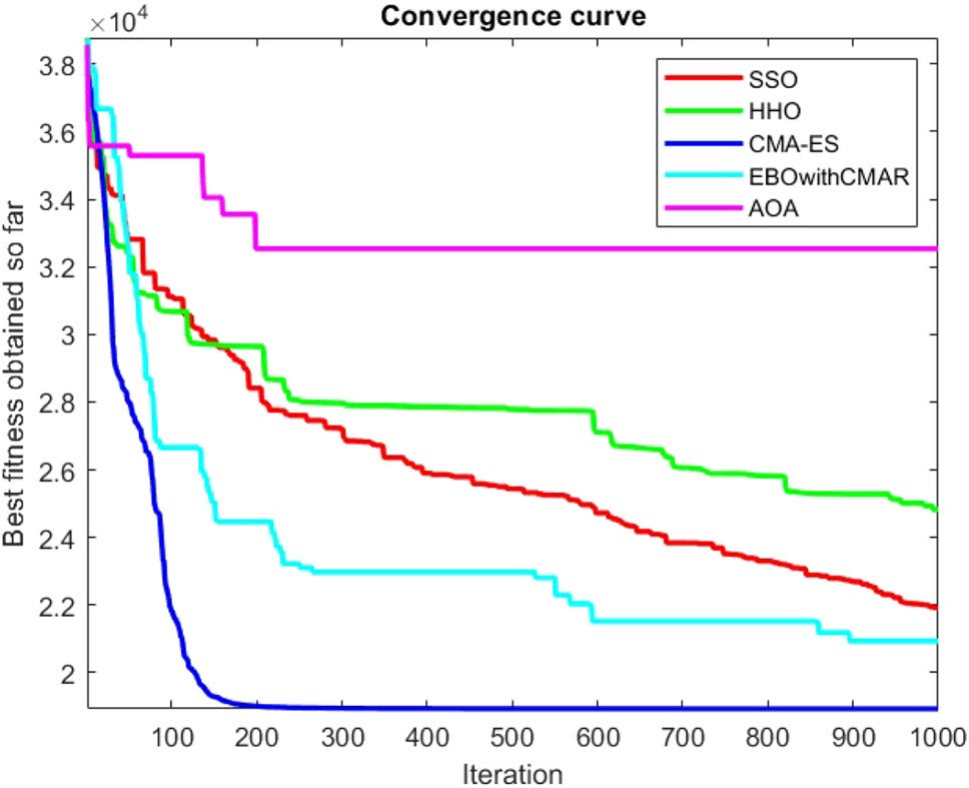
ComplexTest function 22 (Dimension = 100).

**Figure 133:**
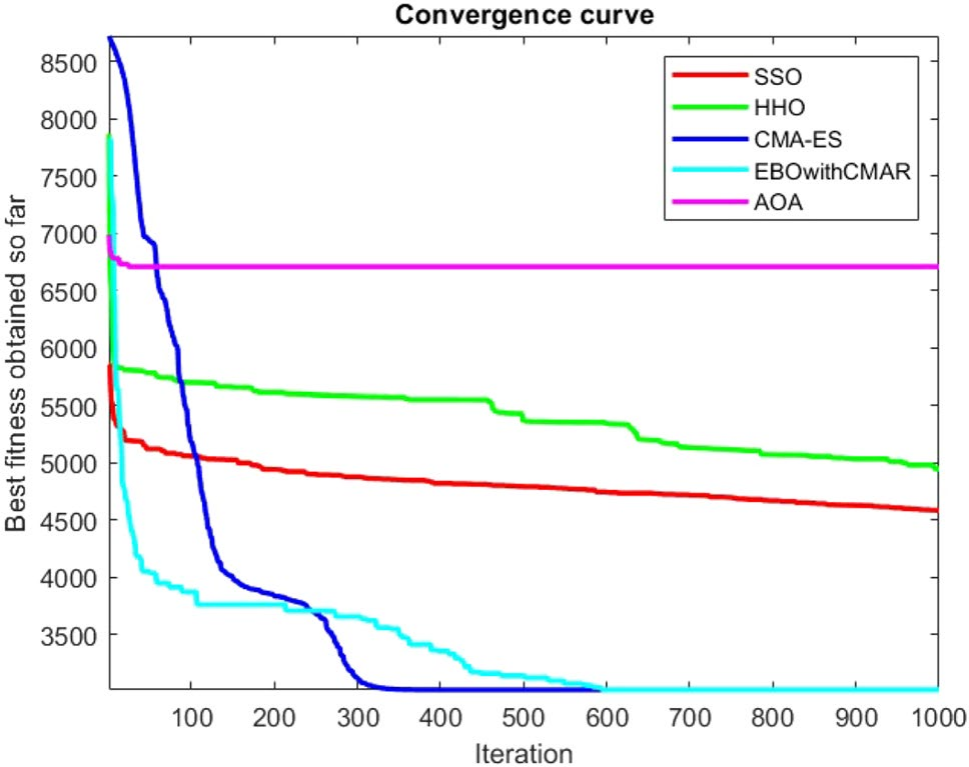
ComplexTest function 23 (Dimension = 100).

**Figure 134:**
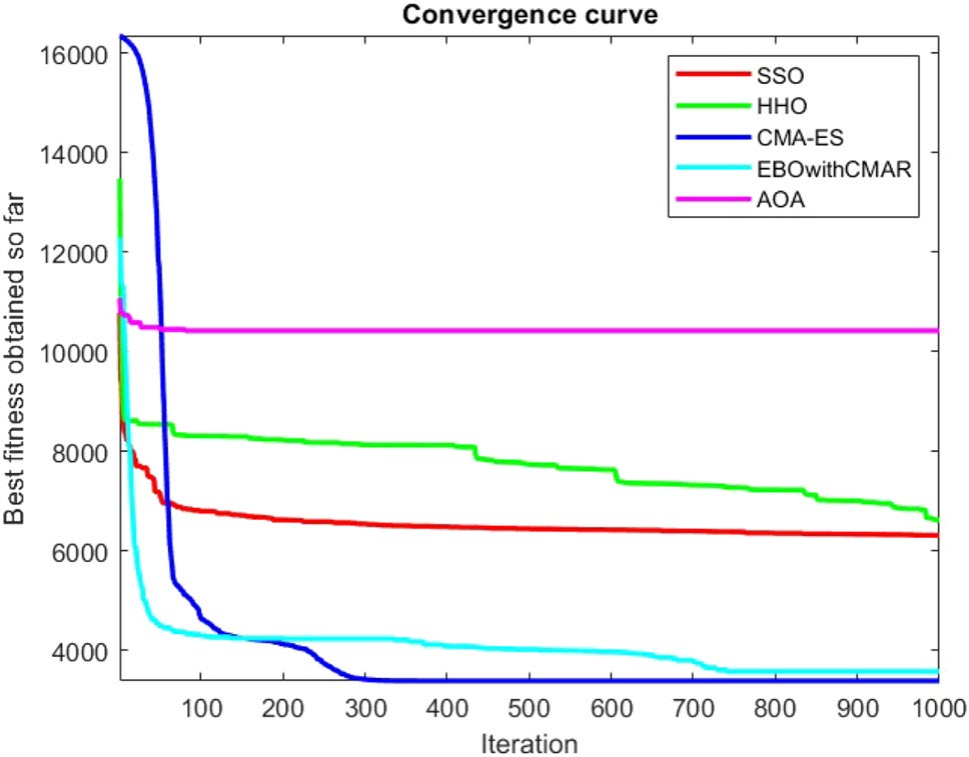
ComplexTest function 24 (Dimension = 100).

**Figure 135:**
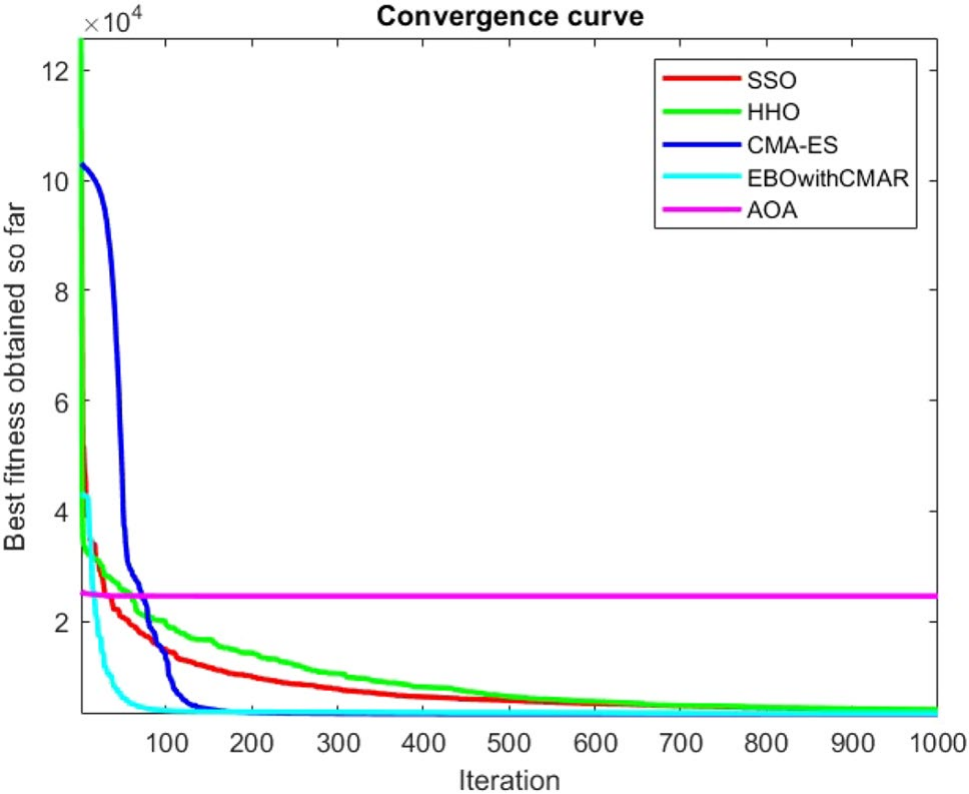
ComplexTest function 25 (Dimension = 100).

**Figure 136:**
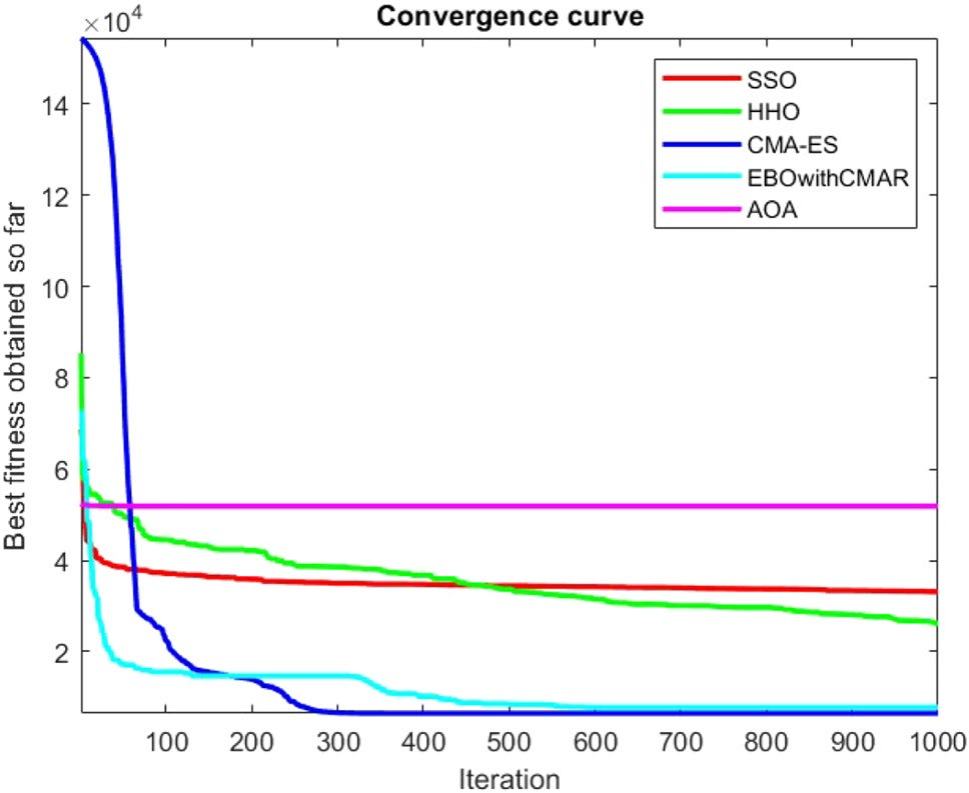
ComplexTest function 26 (Dimension = 100).

**Figure 137:**
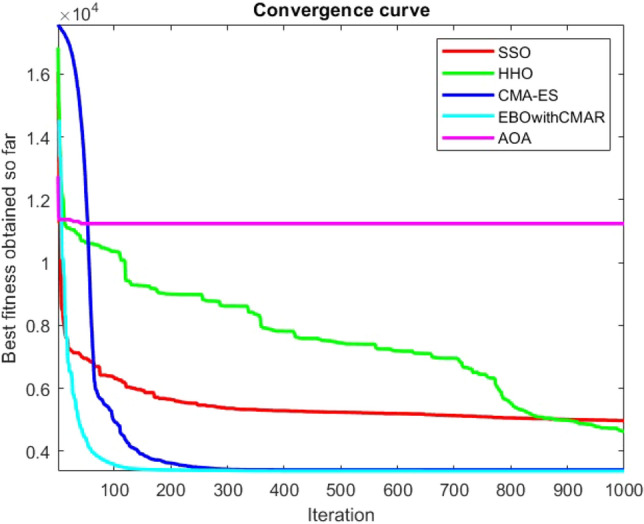
ComplexTest function 27 (Dimension = 100).

**Figure 138:**
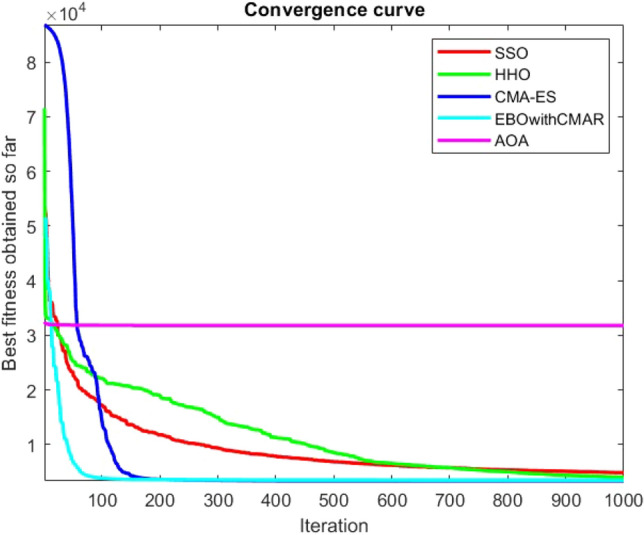
ComplexTest function 28 (Dimension = 100).

**Figure 139:**
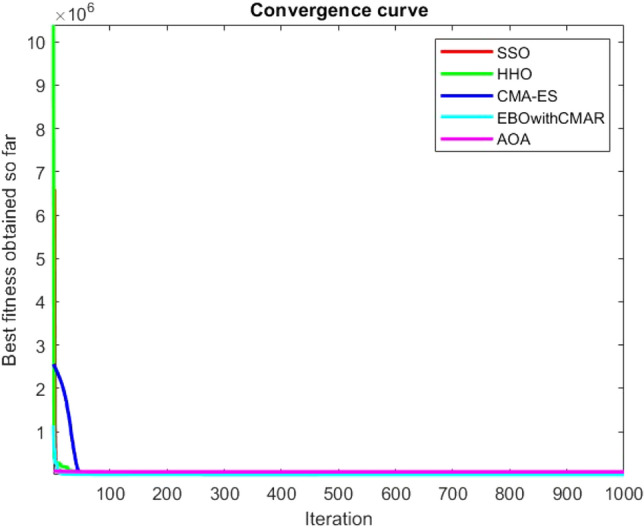
Complex test function 29 (Dimension = 100).

**Figure 140:**
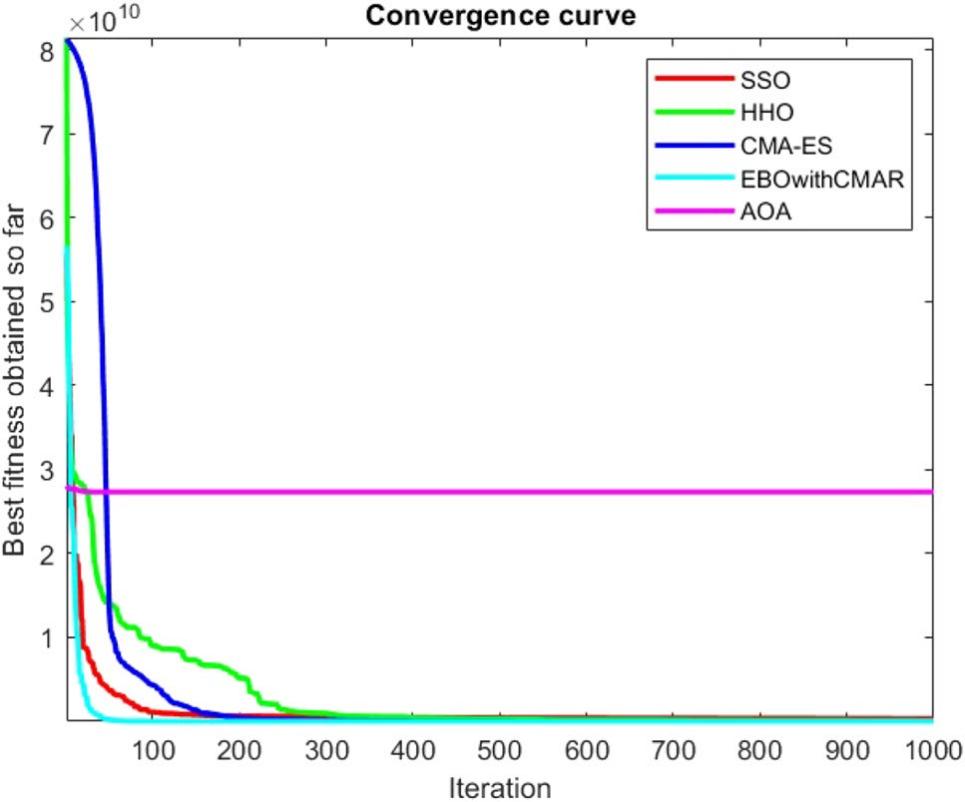
Complex Test function 30 (Dimension = 100).

## Statistical analysis

**Figure 141:**
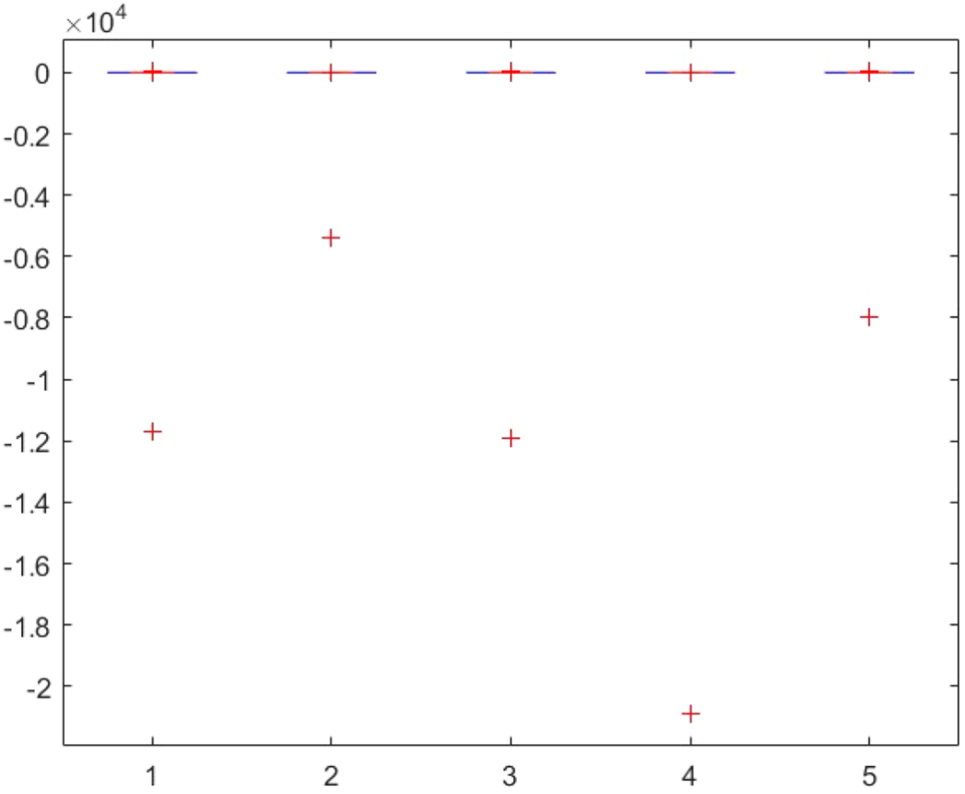
ANOVA test results of each algorithm for the mathematical test functions(mean values).

**Figure 142:**
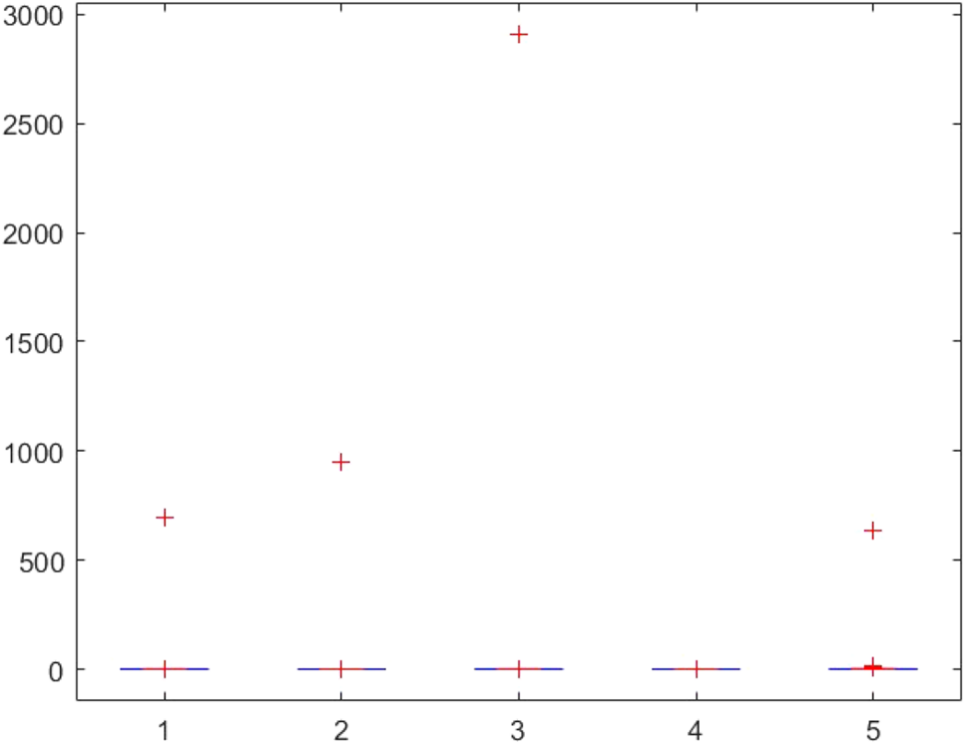
ANOVA test results of each algorithm for the mathematical test functions(standard deviation values).

**Figure 143:**
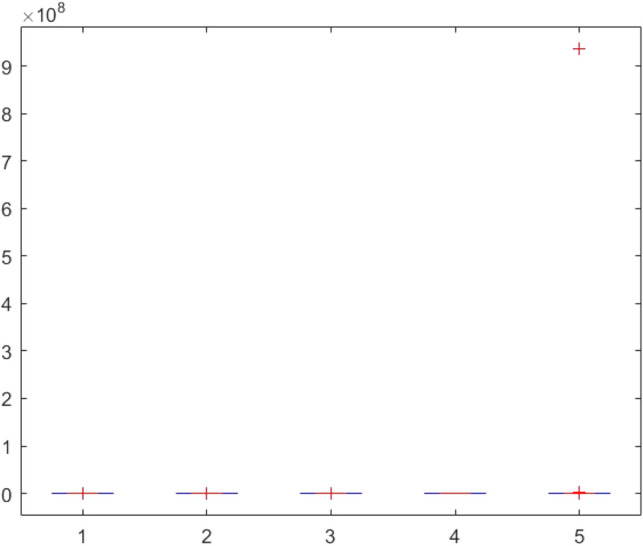
ANOVA test results of each algorithm for CEC-BC-2017(with dimensions = 10, mean values).

**Figure 144:**
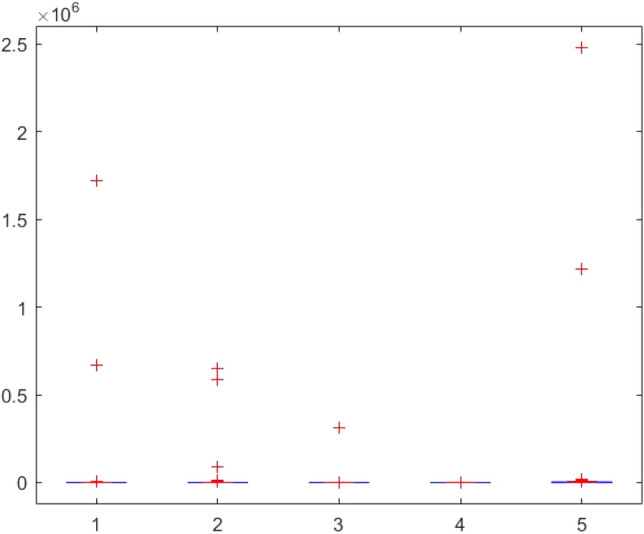
ANOVA test results of each algorithm for CEC-BC-2017(with dimensions = 10, standard devation values).

**Figure 145:**
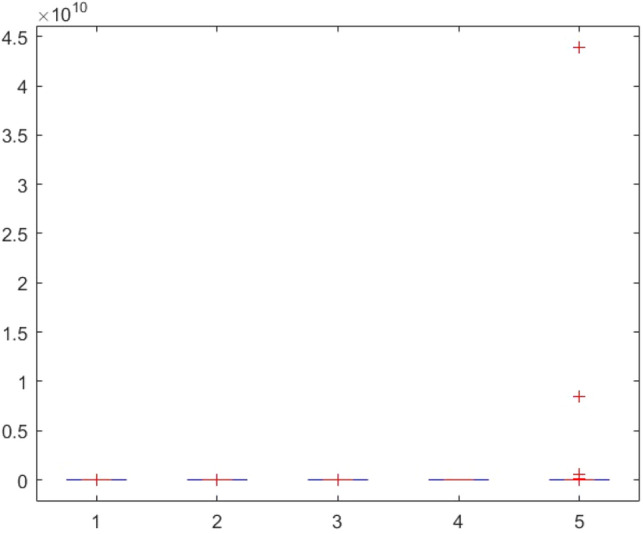
ANOVA test results of each algorithm for CEC-BC-2017(with dimensions = 30, mean values).

**Figure 146:**
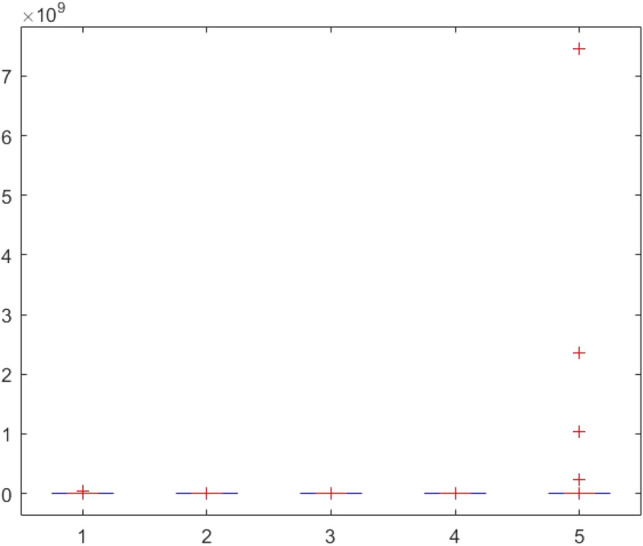
ANOVA test results of each algorithm for CEC-BC-2017(with dimensions = 30, standard deviation values).

**Figure 147:**
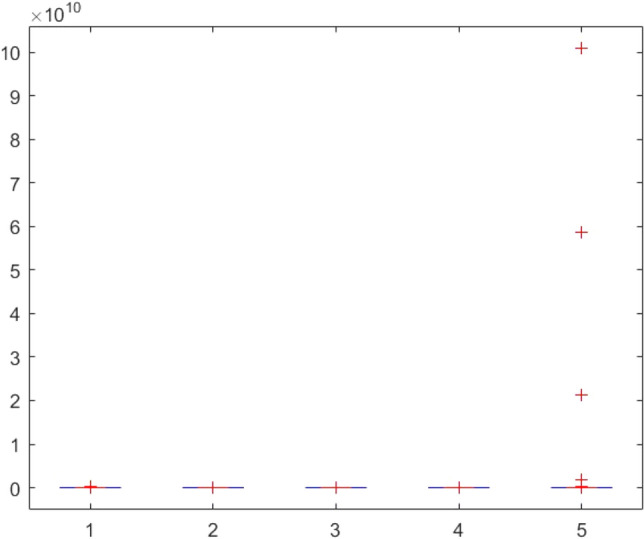
ANOVA test results of each algorithm for CEC-BC-2017(with dimensions = 50, mean values).

**Figure 148:**
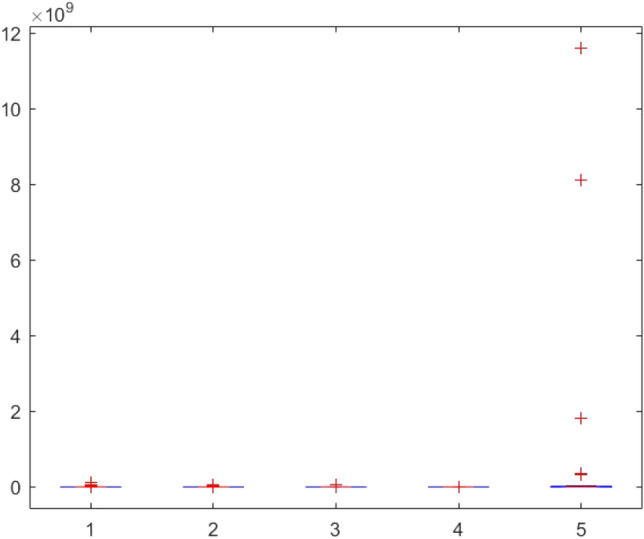
ANOVA test results of each algorithm for CEC-BC-2017(with dimensions = 50, standard deviation values).

**Figure 149:**
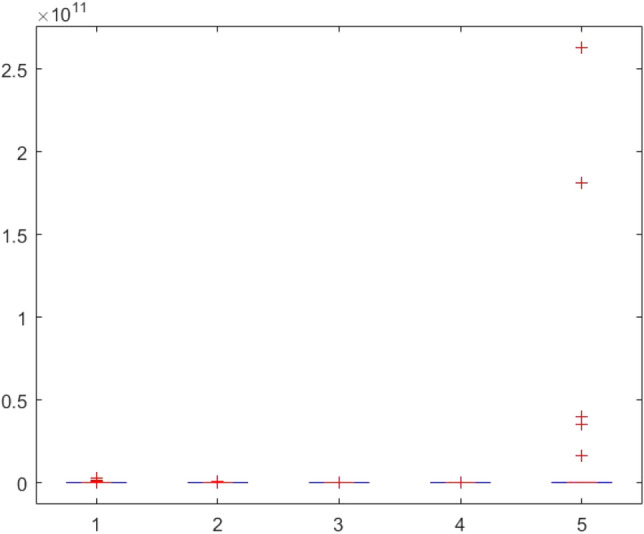
ANOVA test results of each algorithm for CEC-BC-2017(with dimensions = 100, mean values).

**Figure 150:**
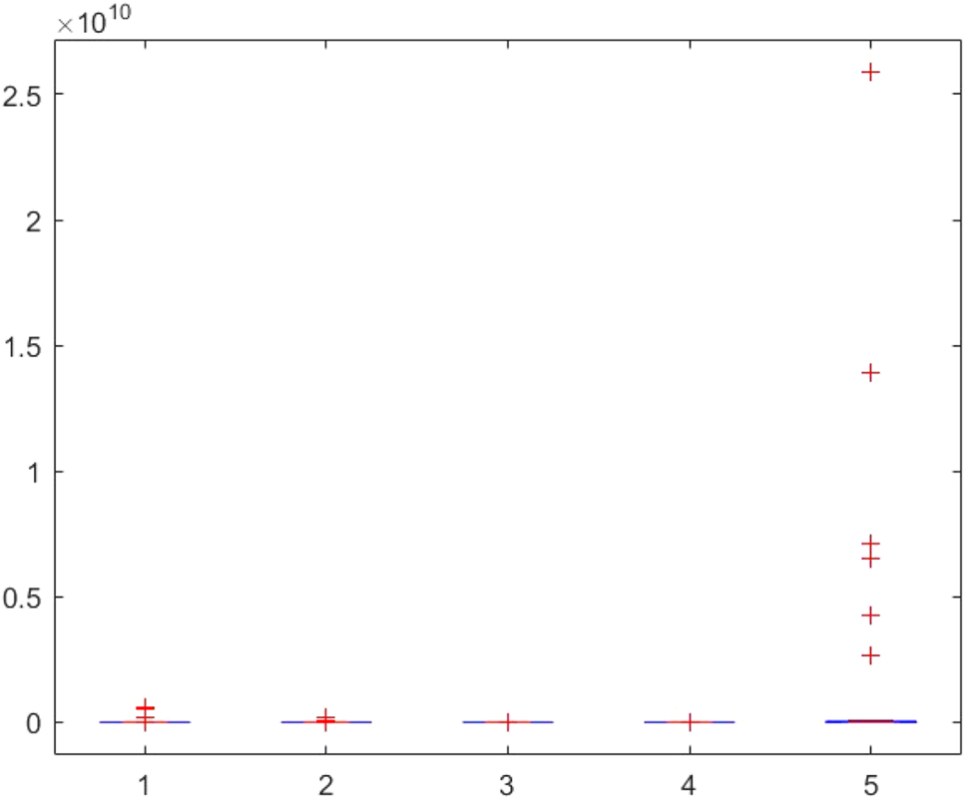
ANOVA test results of each algorithm for CEC-BC-2017(with dimensions = 100, standard deviation values).

This section provides two statistical tests conducted on the obtained results in order to specify the overall performance of the metaheuristics used in this paper among the competitors. The Kruskal–Wallis test, Ttest, Ftest and ANOVA test are considered for this purpose. The statistical analysis is conducted for both mathematical test functions and CEC-BC-2017 functions and presented in Tables 12, 13, 14, 15, 16, 17, 18, 19, 20, 21, 22, 23, 24, 25, 26, 27, 28, 29, 30, 31, 32, 33, 34, 35, 36, 37, 38, 39, 40, 41. Figures 141, 142, 143, 144, 145, 146, 147, 148, 149, 150 show the ANOVA test results of all algorithms.

**Table 12:** The KS test results (p values) for mathematical test functions (mean values).

Main algorithm	Alternative algorithms			
	GOA	TTA	HHO	AOA
SSO				
*p*-value	0.5930	1.0000	0.5930	0.5930

**Table 13:** The KS test results (p values) for mathematical test functions (standard deviation values).

Main algorithm	Alternative algorithms			
	GOA	TTA	HHO	AOA
SSO				
*p*-value	0.8420	0.9832	0.5930	0.1951

**Table 14:** The KS test results (p values) for CEC-BC-2017-Dimnesion = *10 functions (mean values).*

Main algorithm	Alternative algorithms			
	HHO	CMA-ES	EBOwithCMAR	AOA
SSO				
*p*-value	0.9961	0.7406	0.3213	0.5141

**Table 15:** The KS test results (p values) for CEC-BC-2017-Dimnesion = 10 functions (standard deviation values).

Main algorithm	Alternative algorithms			
	HHO	CMA-ES	EBOwithCMAR	AOA
SSO				
*p*-value	0.1844	0.5141	6.81e-07	0.0483

**Table 16:** The KS test results (p values) for CEC-BC-2017-Dimnesion = 30 functions (mean values).

Main algorithm	Alternative algorithms			
	HHO	CMA-ES	EBOwithCMAR	AOA
SSO				
*p*-value	0.7406	0.1844	0.0014	0.0980

**Table 17:** The KS test results (p values) for CEC-BC-2017-Dimnesion = 30 functions (standard deviation values).

Main algorithm	Alternative algorithms			
	HHO	CMA-ES	EBOwithCMAR	AOA
SSO				
*p*-value	0.3212	0.1844	1.15e-05	0.3213

**Table 18:** The KS test results (p values) for CEC-BC-2017-Dimnesion = 50 functions (mean values).

Main algorithm	Alternative algorithms			
	HHO	CMA-ES	EBOwithCMAR	AOA
SSO				
*p*-value	0.9270	0.1844	0.0014	0.0980

**Table 19:** The KS test results (p values) for CEC-BC-2017-Dimnesion = 50 functions (standard deviation values).

Main algorithm	Alternative algorithms			
	HHO	CMA-ES	EBOwithCMAR	AOA
SSO				
*p*-value	0.9961	0.5141	0.0037	0.3213

**Table 20:** The KS test results (p values) for CEC-BC-2017-Dimnesion = 100 functions (mean values).

Main algorithm	Alternative algorithms			
	HHO	CMA-ES	EBOwithCMAR	AOA
SSO				
*p*-value	0.9961	0.0980	4.24e-05	0.1844

**Table 21:** The KS test results (p values) for CEC-BC-2017-Dimnesion = 100 functions (standard deviation values).

Main algorithm	Alternative algorithms			
	HHO	CMA-ES	EBOwithCMAR	AOA
SSO				
*p*-value	0.9270	0.5141	0.0014	0.5141

**Table 22:** The Ttest results (p values) for mathematical test functions (mean values).

Main algorithm	Alternative algorithms			
	GOA	TTA	HHO	AOA
SSO				
*p*-value	0.3319	0.3421	0.3260	0.3260
tstat	-0.9922	0.9711	1.0046	1.0046
sd	1.31e + 03	45.9436	1.92e + 03	1.92e + 03

**Table 23:** The Ttest results (p values) for mathematical test functions (standard deviation values).

Main algorithm	Alternative algorithms			
	GOA	TTA	HHO	AOA
SSO				
*p*-value	0.3251	0.3284	0.3251	0.4550
tstat	1.0066	-0.9995	1.0066	0.7606
sd	144.7378	461.9563	144.7378	12.6974

**Table 24:** The Ttest results (p values) for CEC-BC-2017-Dimnesion = 10 functions (mean values).

Main algorithm	Alternative algorithms			
	HHO	CMA-ES	EBOwithCMAR	AOA
SSO				
*p*-value	0.3986	0.2198	0.1945	0.3251
tstat	0.8573	1.2553	1.3293	-1.0017
sd	1.67E + 05	2.28E + 05	2.36E + 05	1.73E + 08

**Table 25:** The Ttest results (p values) for CEC-BC-2017-Dimnesion = 10 functions (standard deviation values).

Main algorithm	Alternative algorithms			
	HHO	CMA-ES	EBOwithCMAR	AOA
SSO				
*p*-value	0.3615	0.2391	0.1968	0.4861
tstat	0.9278	1.2029	1.3223	-0.7059
sd	2.11E + 05	3.23E + 05	3.38E + 05	3.51E + 05

**Table 26:** The Ttest results (p values) for CEC-BC-2017-Dimnesion = 30 functions (mean values).

Main algorithm	Alternative algorithms			
	HHO	CMA-ES	EBOwithCMAR	AOA
SSO				
*p*-value	0.3403	0.1980	0.1977	0.2424
tstat	0.9701	1.3185	1.3196	-1.1942
sd	6.58E + 06	8.35E + 06	8.35E + 06	8.23E + 09

**Table 27:** The Ttest results (p values) for CEC-BC-2017-Dimnesion = 30 functions (standard deviation values).

Main algorithm	Alternative algorithms			
	HHO	CMA-ES	EBOwithCMAR	AOA
SSO				
*p*-value	0.2367	0.2052	0.2044	0.1655
tstat	1.2092	1.2970	1.2993	-1.4242
sd	5.28E + 06	6.57E + 06	6.57E + 06	1.43E + 09

**Table 28:** The Ttest results (p values) for CEC-BC-2017-Dimnesion = 50 functions (mean values*).*

Main algorithm	Alternative algorithms			
	HHO	CMA-ES	EBOwithCMAR	AOA
SSO				
*p*-value	0.1988	0.1251	0.1143	0.1250
tstat	1.3163	1.5812	1.6300	-1.5814
sd	3.11E + 07	4.60E + 07	4.61E + 07	2.14E + 10

**Table 29:** The Ttest results (p values) for CEC-BC-2017-Dimnesion = 50 functions (standard deviation values).

Main algorithm	Alternative algorithms			
	HHO	CMA-ES	EBOwithCMAR	AOA
SSO				
*p*-value	0.1605	0.2983	0.1621	0.0648
Tstat	1.4415	1.0597	1.4360	-1.9219
sd	1.36E + 07	2.68E + 07	2.53E + 07	3.25E + 09

**Table 30:** The Ttest results (p values) for CEC-BC-2017-Dimnesion = 100 functions (mean values).

Main algorithm	Alternative algorithms			
	HHO	CMA-ES	EBOwithCMAR	AOA
SSO				
*p*-value	0.1419	0.1355	0.1349	0.0891
tstat	1.5112	1.5370	1.5396	-1.7611
sd	4.37E + 08	5.79E + 08	5.80E + 08	5.76E + 10

**Table 31:** The Ttest results (p values) for CEC-BC-2017-Dimnesion = 100 functions (standard deviation values).

Main algorithm	Alternative algorithms			
	HHO	CMA-ES	EBOwithCMAR	AOA
SSO				
*p*-value	0.0979	0.1040	0.1037	0.0511
tstat	1.7124	1.6803	1.6819	-2.0379
sd	1.12E + 08	1.47E + 08	1.47E + 08	5.39E + 09

**Table 32:** The Ftest results (p values) for mathematical test functions (mean values).

Main algorithm	Alternative algorithms			
	GOA	TTA	HHO	AOA
SSO				
*p*-value	5.84E-04	0.9310	0.0086	0.0819
fstat	4.7160	0.9633	0.3124	2.1360

**Table 33:** The Ftest results (p values) for mathematical test functions (standard deviation values)

Main algorithm	Alternative algorithms			
	GOA	TTA	HHO	AOA
SSO				
*p*-value	0.1511	4.70E-09	6.83E-49	0.6790
fstat	0.5358	0.0569	8.13E + 04	1.1955

**Table 34:** The Ftest results (p values) for CEC-BC-2017-Dimnesion = 10 functions (mean values).

Main algorithm	Alternative algorithms			
	HHO	CMA-ES	EBOwithCMAR	AOA
SSO				
*p*-value	9.49E-06	2.47E-20	6.70E-60	2.08E-0.73
fstat	5.9872	85.9436	5.88E + 04	1.84E-06

**Table 35:** The Ftest results (p values) for CEC-BC-2017-Dimnesion = 10 functions (standard deviation values).

Main algorithm	Alternative algorithms			
	HHO	CMA-ES	EBOwithCMAR	AOA
SSO				
*p*-value	1.48E-04	5.00E-15	3.20E-108	0.0377
fstat	4.5263	34.8014	1.66E + 08	0.4481

**Table 36:** The Ftest results (p values) for CEC-BC-2017-Dimnesion = 30 functions (mean values).

Main algorithm	Alternative algorithms			
	HHO	CMA-ES	EBOwithCMAR	AOA
SSO				
*p*-value	1.16E-07	9.11E-83	4.75E-101	5.94E-77
fstat	8.9758	2.53E + 06	5.11E-07	1.02E-06

**Table 37:** The Ftest results (p values) for CEC-BC-2017-Dimnesion = 30 functions (standard deviation values).

Main algorithm	Alternative algorithms			
	HHO	CMA-ES	EBOwithCMAR	AOA
SSO				
*p*-value	1.24E-12	4.08E-69	1.61E-116	1.17E-58
fstat	22.8443	2.67E + 05	6.51E + 08	2.08E-05

**Table 38:** The Ftest results (p values) for CEC-BC-2017-Dimnesion = 50 functions (mean values).

Main algorithm	Alternative algorithms			
	HHO	CMA-ES	EBOwithCMAR	AOA
SSO				
*p*-value	4.86E-05	2.55E-31	8.34E-65	8.03E-68
fstat	5.0869	533.0105	1.31E + 05	4.61E-06

**Table 39:** The Ftest results (p values) for CEC-BC-2017-Dimnesion = 50 functions (standard deviation values).

Main algorithm	Alternative algorithms			
	HHO	CMA-ES	EBOwithCMAR	AOA
SSO				
*p*-value	3.89E-04	1.85E-08	1.37E-81	3.27E-52
fstat	4.0729	10.519	2.08E + 06	6.01E-05

**Table 40:** The Ftest results (p values) for CEC-BC-2017-Dimnesion = 100 functions (mean values).

Main algorithm	Alternative algorithms			
	HHO	CMA-ES	EBOwithCMAR	AOA
SSO				
*p*-value	3.31E-09	5.4E-65	1.82E-101	3.69E-49
fstat	14.6866	1.36E + 05	5.47E + 07	9.94E-05

**Table 41:** The Ftest results (p values) for CEC-BC-2017-Dimnesion = 100 functions (standard deviation values).

Main algorithm	Alternative algorithms			
	HHO	CMA-ES	EBOwithCMAR	AOA
SSO				
*p*-value	2.75E-10	1.32E-59	5.87E-95	3.53E-37
fstat	14.9085	5.60E + 04	1.87E + 07	7.14E-04

## Discussion and analysis of results

Mean results and standard deviation of the mathematical test functions are presented in Tables 2 and 3. Tables 4, 5, 6, 7, 8, 9, 10, 11 show the mean results and standard deviation results of the CEC-BC-2017 test functions. According to the provided results presented in Tables 4, 5, 6, 7, 8, 9, 10, 11, the performance of SSO algorithm in solving the specified optimization problems was investigated. From statistical point of view, SSO can provide similar results to those of the algorithms used for comparison of the results. For mathematical test functions. Different optimization problems have been used to examine the suggested SSO algorithm's capabilities, based on the obtained results. From a mathematical and statistical perspective, the algorithm that is being provided can yield highly satisfactory results because it can either approach global optimum solutions or very close values of optimum solutions in the test functions that are employed in this work. In the following section of this study, we will examine the SSO performance in more detail.

A variety of optimization problems have been used to examine the suggested SSO algorithm's capabilities in by providing of the conclusions presented in this section. From the perspective of mathematical functions, the SSO can produce very acceptable results, such as the mean and standard deviation.

The advantages and disadvantages of the strategy given are evident in the produced results as well as the convergence curves. Table 2 reveals that the suggested approach performs comparably and robustly with the state-of-the-art optimization techniques in 14 of the mathematical test functions. Following the No Free Lunch theorem, the SSO algorithm's performance on the remaining mathematical test functions—where it performs poorly—was expected.

While EBOwithCMAR outperforms both the proposed method and the other algorithms used to compare the results with the proposed method for CEC-BC-2017 functions with dimensions of ten, the SSO algorithm ranks second for the majority of the functions after EBOwithCMAR, which is an advantage of the SSO since the proposed method outperforms the well-known CMA-ES optimization algorithm.

One of the primary drawbacks of the suggested approach is that, as the CEC-BC-2017 functions' dimensions are increased, its performance declines. This is evident in Tables 6, 7, 8, 9, 10, 11, where the method yields comparatively weak results in 30, 50, and 100 dimensions than in 10. As a result, it may be said that the SSO algorithm is sensitive to the optimization problem's dimension expansion.

Some interesting results can be deduced from the convergence curves of the 23 mathematical test functions that were utilized to examine the effectiveness of the SSO algorithm The corresponding convergence curves for mathematical test functions 1–7 demonstrate that the suggested approach performs equally with the optimization techniques utilized to compare the acquired results with them. The SSO algorithm is the second method that finds the optimal solution in mathematical test function 8. Once more, the algorithms exhibit equal performance in the mathematical test functions 9–14. It is evident from the convergence curve of mathematical test function 15 that the suggested approach performs comparatively poorly when compared to the other approaches. Mathematical test functions 16–19 show an equal performance among the algorithms. Another weakness of the proposed method is evident in mathematical test function 20 in which a relatively weak performance was observed in converging towards the global optimum of this function according to its convergence curve. Convergence curves of the mathematical test Functions 21–23 indicate that the SSO algorithm is able to outperform its rivals. As the results of the SSO method are, for the most part, extremely close to the global optimum solutions of the test functions, it is noteworthy to state that Tables 2 and 3 demonstrate an acceptable performance of the algorithm.

It is possible to conclude that the suggested algorithm performs on the same level with the best state-of-the-art methods used for comparison by examining Figs. 25, 26, 27, which depict the convergence curves of the CEC-BC-2017 functions numbers 1, 3, 4, in 10 dimensions. This is because the proposed algorithm converges to an optimal solution that is comparable to the other methods. Figures 28, 31 and 33 indicate an excellent performance of the SSO algorithm in finding optimum value for CEC-BC-2017 functions number 5,8 and 10 with 10 dimensions which can be considered as an advantage of this algorithm in dealing with the mentioned test functions. As previously mentioned, one of the main issues with the proposed method is that optimization performance of the proposed method on CEC-BC-2017 functions decreases as their dimensions are raised. Therefore, one may say that the SSO algorithm is sensitive to the dimension expansion of the optimization problem. However according to the results of the mathematical and CEC-BC-2017 test functions provided in tables and figures, it can be concluded that the SSO algorithm has an acceptable performance on optimizing the examined test functions.

## Conclusion

The Stadium Spectators Optimization (SSO) algorithm was presented as a novel metaheuristic algorithm which is inspired by the actions of stadium spectators affecting behavior of players during a match. It was used to optimize some of the well-known mathematical test functions and also CEC-BC-2017 functions. The results were compared to those of other powerful and well-recognized metaheuristics such as HHO, CMA-ES, EBOwithCMAR and AOA. The following conclusions can be drawn from the presented paper:

- SSO was observed to be an efficient method in optimization of the test functions used in this paper, since it provides close results to the other algorithms used in the paper for the purpose of comparison. This can be an indication that the SSO algorithm can be a potential algorithm for solving other optimization problems such as constrained optimum design problems for instance.
- It was observed that by increasing the dimensions of the CEC-BC-2017 test functions, the performance of the SSO algorithm was decreased. It can be considered a sensitivity of dimension expansion of optimization problems. More investigations are needed to analyze this issue by testing the proposed method on some other high dimensional optimization problems to validate this assumption.
- Parametric statistical tests were conducted to analyze the performance of the algorithm since the KS test showed that in most of the cases the *p-*values were greater than 0.05.
- The SSO algorithm can execute exploitation in a rather acceptable manner and extremely close to the performance of the comparable algorithms.
- The SSO demonstrates that it is a reasonably effective approach for handling the test functions for examining its own exploration capabilities and yields results that enable it to compete favorably with superior high performance optimization algorithms.

For future directions, using the presented method as an optimizer in engineering optimum design problems can be suggested to test its ability in different optimization problems. Also new configurations of the SSO algorithm can be considered as the methodology presented in the paper may have some other different formulations in the opinion of other researchers. Improving the sensitivity to dimension expansion which exists in the current version of the SSO algorithm can be beneficial. Perhaps providing a better search operator for the current SSO algorithm can fix this issue. Changing the mechanism which controls the balance between exploration and exploitation of the proposed method may help to search areas of search space which the current version of the proposed optimization algorithm is ignoring. Another suggestion could be to use extra parameters to control the movement of search agents in this algorithm and enhancing its searching ability.

## Data Availability

The datasets used and/or analysed during the current study available from the corresponding author on reasonable request.
